# Heterologous Expression of the Formicamycin Biosynthetic Gene Cluster Unveils Glycosylated Fasamycin Congeners

**DOI:** 10.1021/acs.jnatprod.3c00052

**Published:** 2023-06-16

**Authors:** Hannah P. McDonald, Abigail Alford, Rebecca Devine, Edward S. Hems, Sergey A. Nepogodiev, Corinne J. Arnold, Martin Rejzek, Anna Stanley-Smith, Neil A. Holmes, Matthew I. Hutchings, Barrie Wilkinson

**Affiliations:** † Department of Molecular Microbiology, John Innes Centre, Norwich Research Park, Norwich, NR4 7UH, U.K.; ‡ NMR Platform, John Innes Centre, Norwich Research Park, Norwich NR4 7UH, U.K.; § Chemistry Platform, John Innes Centre, Norwich Research Park, Norwich NR4 7UH, U.K.; ∥ Isomerase, Chesterford Research Park, Cambridge CB10 1XL, U.K.

## Abstract

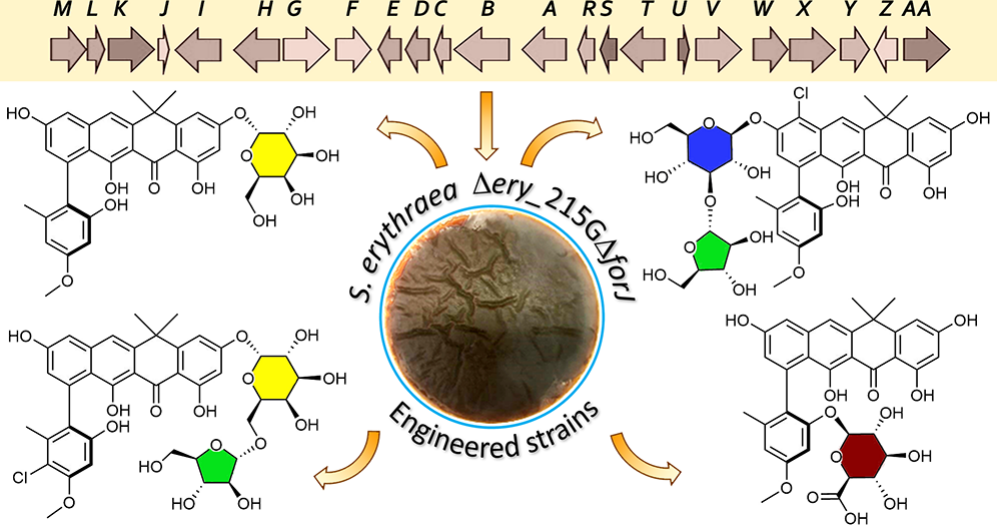

Formicamycins and their biosynthetic intermediates the fasamycins are polyketide antibiotics produced by *Streptomyces formicae* KY5 from a pathway encoded by the *for* biosynthetic gene cluster. In this work the ability of *Streptomyces coelicolor* M1146 and the ability of *Saccharopolyspora erythraea* Δ*ery* to heterologously express the *for* biosynthetic gene cluster were assessed. This led to the identification of eight new glycosylated fasamycins modified at different phenolic groups with either a monosaccharide (glucose, galactose, or glucuronic acid) or a disaccharide comprised of a proximal hexose (either glucose or galactose), with a terminal pentose (arabinose) moiety. In contrast to the respective aglycones, minimal inhibitory screening assays showed these glycosylated congeners lacked antibacterial activity.

Formicamycins, and their biosynthetic intermediates the fasamycins (Figure 1), are antibacterial natural products produced by *Streptomyces formicae* KY5 which was isolated from the domatia (nests) of *Tetraponera penzigi* plant ants indigenous to Kenya.^1,2^ Fasamycin congeners had already been reported following the heterologous expression of environmental DNA isolated from soil and were named as such after they were shown to be inhibitors of the bacterial fatty acid synthase (FAS) condensing enzyme FabF.^3^ Fasamycin congeners have also been reported as the products of several other actinomycete strains under the names streptovertimycins, naphthacemycins, and accramycins.^4−6^

**Figure 1:**
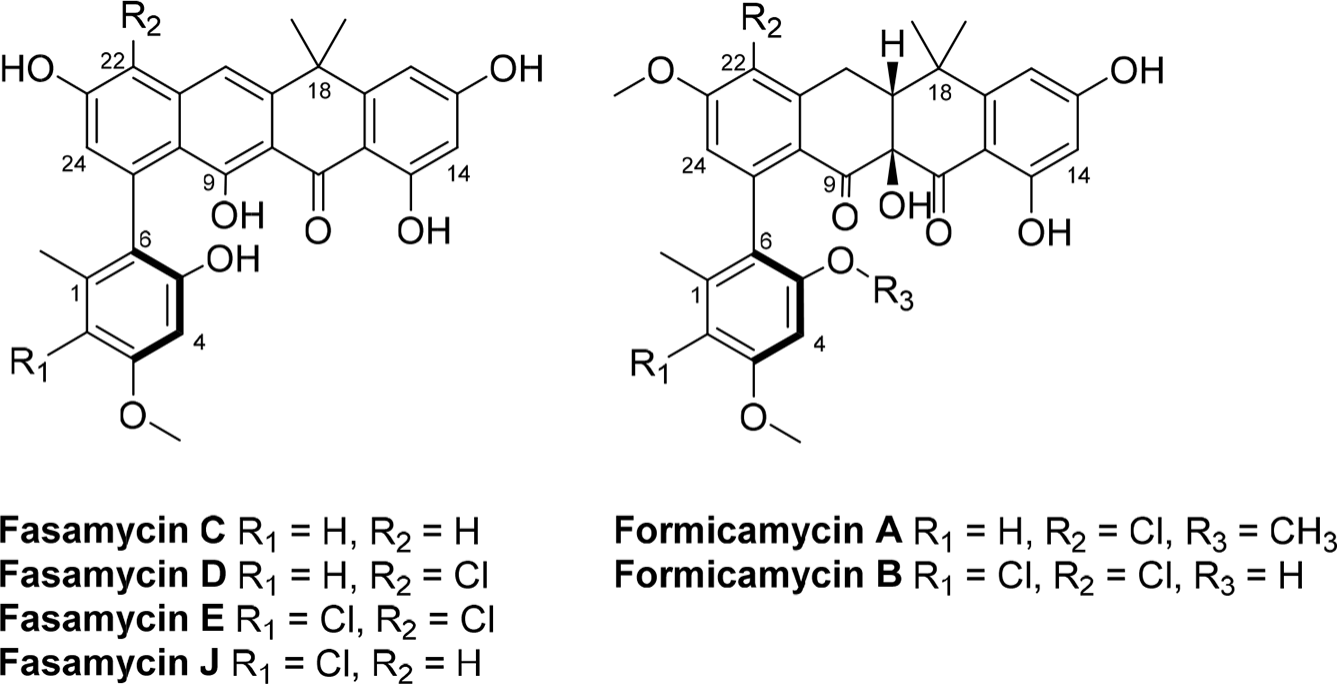
Structures of fasamycin congeners C, D, E, and J, and formicamycin congeners A and B.

Fasamycins and formicamycins display potent antimicrobial activities against clinically relevant Gram-positive bacteria including methicillin-resistant *Staphylococcus aureus* (MRSA) and vancomycin-resistant enterococci (VRE). Both groups of compounds are produced from the formicamycin (*for*) biosynthetic gene cluster (BGC) in *S. formicae* KY5, which encodes a type II polyketide synthase.^1^ The products of this pathway are all chlorinated except for fasamycin C, and recent work has shown that 24 genes, encoded on nine transcripts, are required for formicamycin biosynthesis.^7^ Regulation of the *for* BGC is heavily dependent on the MarR-family regulator ForJ which represses expression of seven of the nine transcripts encoding the pathway, and we have shown that derepression via deletion of *forJ* increases products of the *for* pathway 6.7-fold in the host producer. When combined with the overexpression of the cluster specific regulator genes *forGF* (in the Δ*forJ* background), this leads to a more than 10-fold increase in titer.^7^

With this knowledge in hand, we investigated the potential of using a derepressed *for* expression construct to produce high yielding heterologous expression strains. To achieve this, we introduced a phage-derived artificial chromosome (PAC) containing the *for* BGC (called pESAC-13_215G (abbreviated to 215G)) in which the *forJ* gene had been deleted (215GΔ*forJ*), into the heterologous host strains *Streptomyces coelicolor* M1146 and *Saccharopolyspora erythraea* Δ*ery* with the aim of overproducing fasamycins and formicamycins. We report that *S. coelicolor* M1146_215GΔ*forJ* produces formicamycins at moderate levels in comparison to wild-type (WT) *S. formicae*, while *Sacch. erythraea* Δ*ery*_215GΔ*forJ* produces a previously identified fasamycin congener as well as several novel glycosylated fasamycin congeners. The structures of these glycosylated molecules are reported here.

## Results and Discussion

### Deletion of *forJ* Is Required to Produce Fasamycins and Formicamycins in Heterologous Hosts

**Figure 2:**
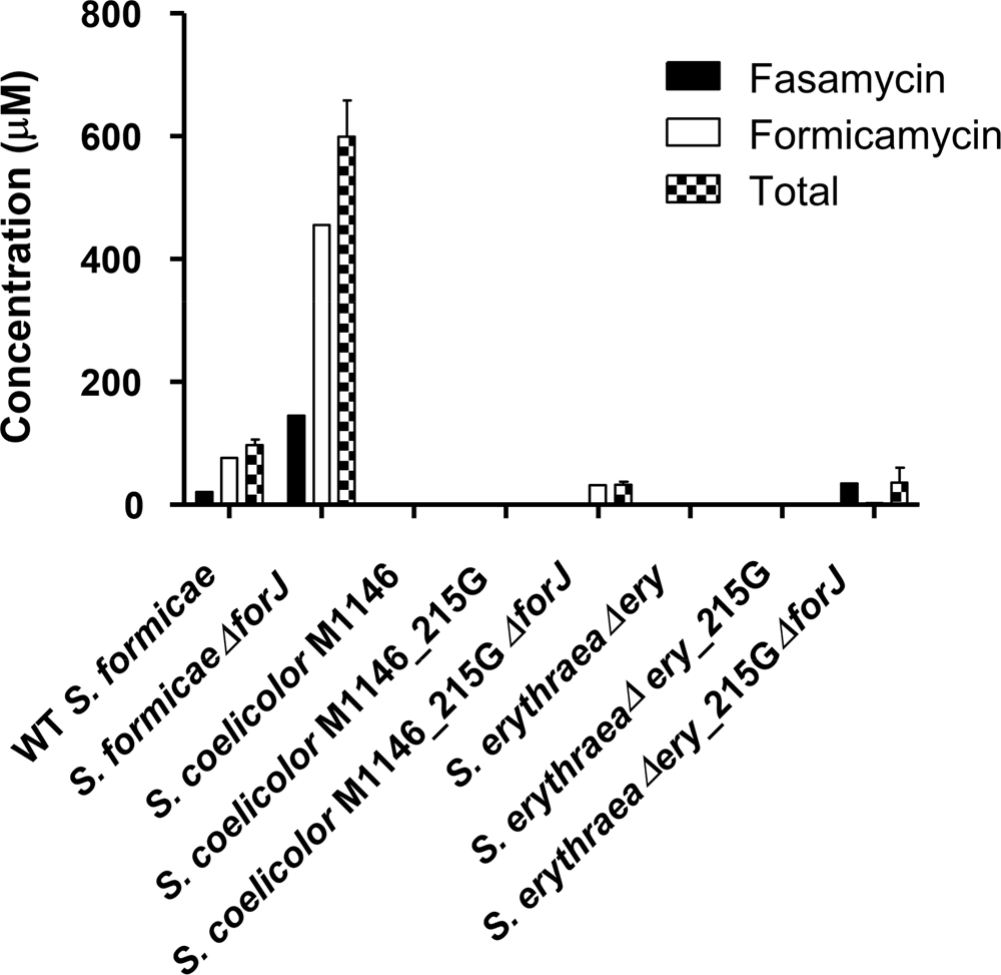
Titers of total fasamycin and formicamycin congeners produced on solid SFM medium from *S. formicae*, *S. coelicolor*, and *Sacch. erythraea* strains used in this study. Error bars represent standard deviations.

The heterologous expression strains *S. coelicolor* M1146_215G and *Sacch. erythraea* Δ*ery*_215G were constructed via successful genomic integration of the previously reported PAC pESAC-13_215G into the φC31 phage-1 integration site.^8^ The insert cloned into pESAC-13_215G spans the whole of the *for* BGC plus 40–80 kb of additional DNA on either side. The pESAC-13_215G was moved into the heterologous host strains via triparental mating into *Escherichia coli* ET12567 using the pR9604 transfer plasmid, followed by conjugal transfer to the actinomycete host.^9^ The resulting strains thus contain the full *for* BGC, including the *forJ* coding region, and were grown on solid soya flour mannitol (SFM) agar as described previously.^1,7^ As anticipated, analysis by LCMS of ethyl acetate extracts generated from these plates after 7 days of incubation at 30 °C did not indicate any fasamycins or formicamycin congeners under the growth conditions tested (Figure 2 and Table S1, Supporting Information). In contrast, the control strains *S. formicae* (WT) and *S. formicae* Δ*forJ* produced fasamycin and formicamycin congeners as reported previously.^7^

Given that deletion of *forJ* in *S. formicae* both increases production and enables the production in liquid culture,^7^ we replaced *forJ* in pESAC-13_215G with an apramycin resistance gene using a PCR targeting approach to yield pESAC-13_215GΔ*forJ*.^10^ pEASC13_215GΔ*forJ* was then transferred into both hosts by conjugal transfer and the resulting strains were grown on SFM agar. Ethyl acetate extracts were again analyzed for fasamycin and formicamycin production by LCMS. *S. coelicolor* M1146_215GΔ*forJ* produced several previously identified fasamycin and formicamycin congeners, whereas *Sacch. erythraea* Δ*ery*_215GΔ*forJ* produced the nonchlorinated fasamycin C in addition to the known formicamycins A and B, alongside a series of new compounds identified as fasamycin congeners based on inspection of their UV spectra and MS data (*vide infra*). As *S. formicae* does not produce products of the *for* BGC in liquid culture, unless the BGC has been derepressed by deleting *forJ,*(7) we checked whether this is the case for *S. coelicolor* M1146 and *Sacch. erythraea* Δ*ery* strains containing both the wild-type PAC (215G) and derepressed PAC (215GΔ*forJ)*, but no production of any congeners was observed in either case.

Quantitative analyses of the metabolites produced by each strain grown on SFM agar were then undertaken to investigate their potential to yield improved titers. Total fasamycin and formicamycin production from the WT *S. formicae* was determined to be 96.1 ± 9.6 μM with fasamycin congeners accounting for 21% of this. Analysis of fasamycin production by *S. coelicolor* M1146_215GΔ*forJ* showed a significant reduction in total fasamycin production in comparison to WT *S. formicae* (0.4 ± 0.3 vs 20.6 ± 6.1 μM), and formicamycin titers were approximately half of those determined for WT *S. formicae* (31.2 ± 5.1 vs 75.5 ± 3.5 μM). Moreover, the total combined production of fasamycin/formicamycins production by *S. coelicolor* M1146_215GΔ*forJ* fell short of that by the derepressed strain *S. formicae* Δ*forJ* by ∼20-fold (Table S1, Supporting Information).

*Sacch. erythraea* Δ*ery*_215GΔ*forJ* was found to only produce trace quantities of two formicamycin congeners (A and B). However, the total production of fasamycins was approximately equal to that of WT *S. formicae* but significantly reduced in comparison to that of *S. formicae* Δ*forJ* (Figure 2 and Table S1, Supporting Information). As heterologous expression did not lead to improved production, this led us to the conclusion that heterologous expression (at least in these two strains) is not a suitable route for the enhanced production of these compounds.

### Heterologous Expression of the *for* BGC Yields Glycosylated Fasamycin Congeners

**Figure 3:**
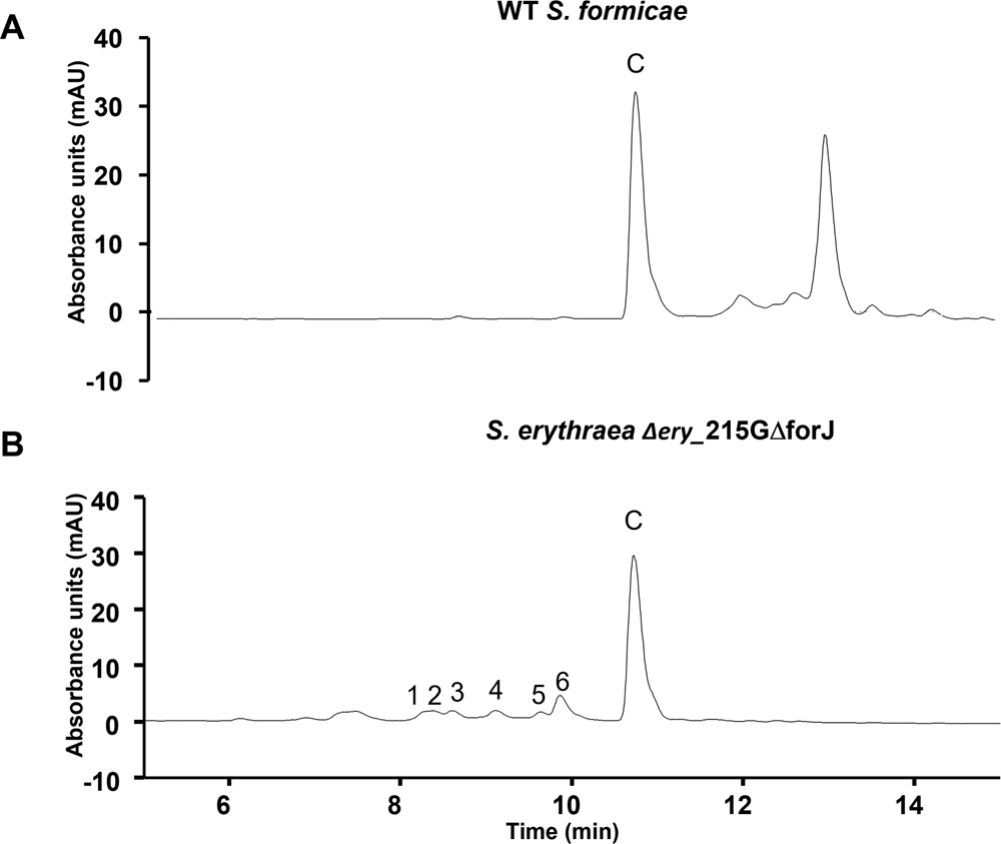
*Sacch. erythraea* Δ*ery*_215GΔ*forJ* produces several new fasamycin congeners. HPLC chromatograms were recorded at 418 nm. (A) *S. formicae* WT (control) showing a major fasamycin C peak. (B) *Sacch. erythraea* Δ*ery*_215GΔ*forJ* extracts show six peaks (1–6) comprising new compounds and fasamycin C.

As noted above, despite the lack of improved titers after heterologous expression of the *for* BGC, careful inspection of the chromatograms for the extracts from analytical fermentation of *Sacch. erythraea* Δ*ery*_215GΔ*forJ* grown on SFM agar revealed the presence of six peaks consistent with new fasamycin congeners (Figure 3B). These peaks displayed the characteristic fasamycin UV–vis spectra (Figure S1, Supporting Information) and were notable due to their early elution times on reversed-phase C_18_ chromatography columns indicating they were more polar than fasamycin C and other known congeners. Together these new congeners accounted for ∼15% of the total fasamycins produced by *Sacch. erythraea* Δ*ery*_215GΔ*forJ*, with fasamycin C accounting for 75% as determined by titer analysis. Upon reinspection of earlier acquired HPLC and LCMS data, we found that several of these new congeners were also produced by *S. coelicolor* M1146_215GΔ*forJ*, but at trace levels.

**Figure 4:**
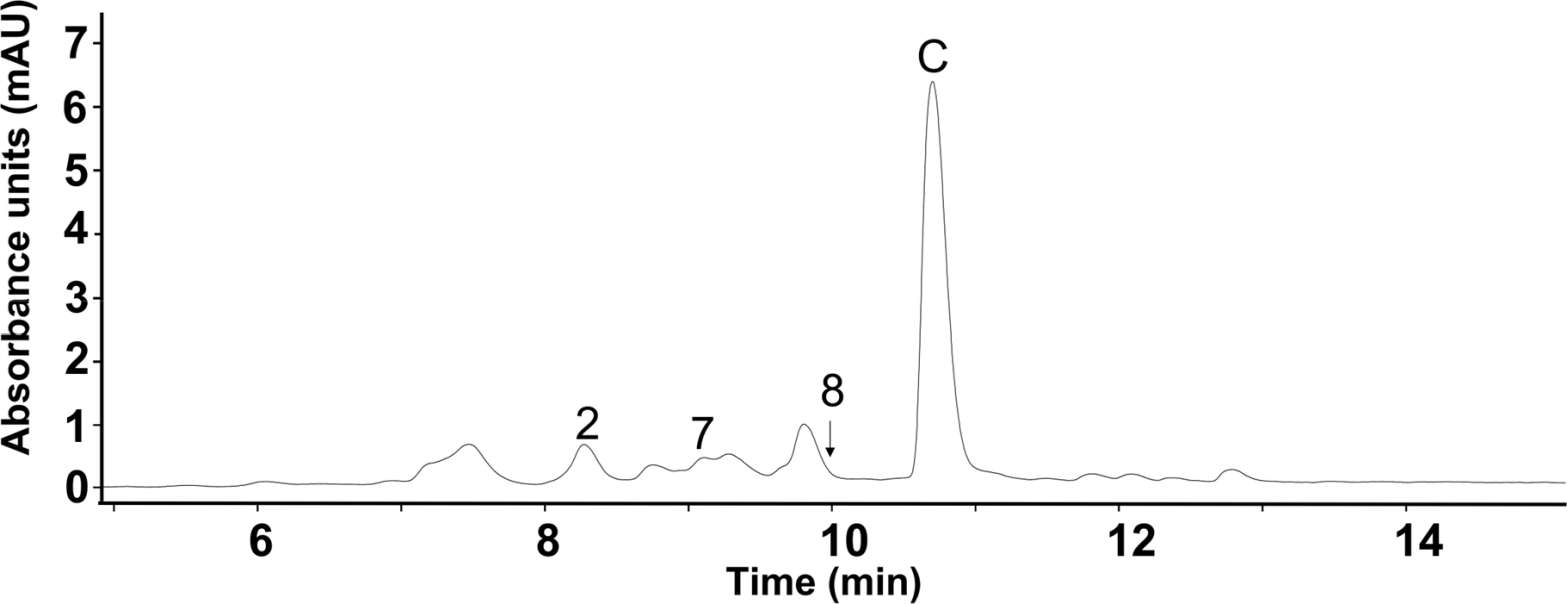
First upscaled growth of *Sacch. erythraea* Δ*ery*_215GΔ*forJ* produces two new peaks, 7 and 8. HPLC chromatogram recorded at 418 nm. The major peak “C” is fasamycin C.

Analysis of LCMS/MS data revealed *m*/*z* values for peaks 1–6 that suggested the corresponding compounds were glycosides consisting of fasamycin C or a monochlorinated fasamycin (fasamycin D or J) with either a single hexose unit (monosaccharide) or disaccharides comprising a hexose and a pentose.^11^ However, these data were complicated by the fact that peaks 1 and 5 (Figure 3B) contain parent ions for three and two coeluting congeners of similar structures, respectively, which we showed by a combination of HPLC and extracted ion chromatograms (EICs) (Figures S2 and S8, Supporting Information). To complicate matters further, when we upscaled growth of *Sacch. erythraea* Δ*ery*_215GΔ*forJ* on SFM agar for metabolite isolation, our first attempt showed the production of two new peaks, 7 and 8 (Figure 4), together with the previously observed peak 2, while the compounds associated with peaks 1, 3, and 4–6 were produced only in trace quantities; only peaks 2, 7, and 8 could be purified in high enough quantities for structural elucidation. However, upon repeating the upscaled growth, we obtained an extract for which the HPLC and LCMS chromatograms were consistent with the initial analytical scale growths.

**Figure 5:**
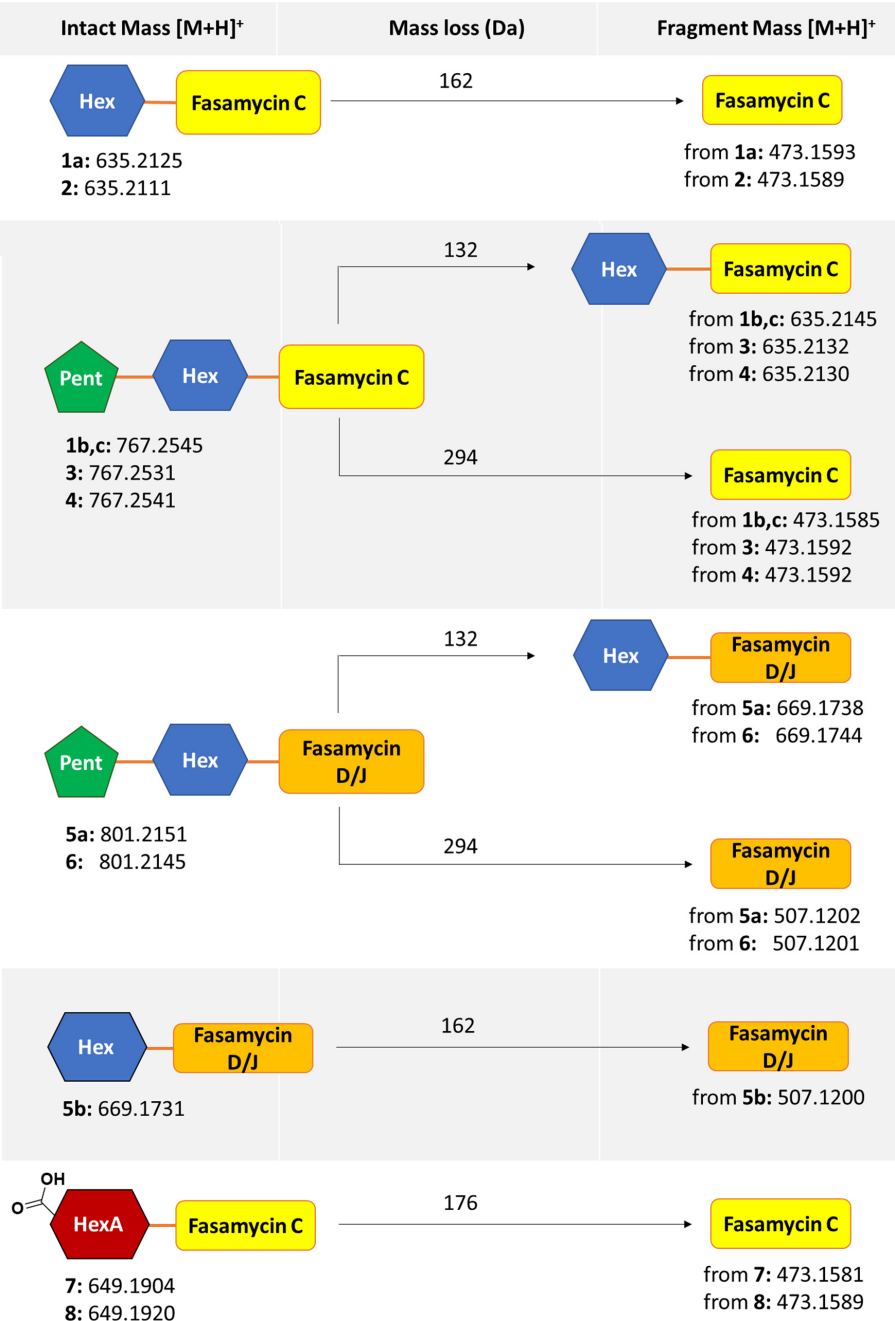
MS/MS fragmentation analysis of compounds present in samples 1–6 and in-source fragmentation of compounds present in samples 7 and 8 isolated from *Sacch. erythraea* Δ*ery*_215GΔ*forJ* grown on SFM agar indicating the presence of glycosylated fasamycins. The details of the analyses and LCMS/MS spectra are shown in Figures S2–S13 (Supporting Information).

The first upscaled extract comprising peaks 7 and 8 was analyzed by LCMS, and it was consistent with the loss of a single hexuronic acid moiety from the fasamycin C aglycone. A summary of the LCMS analysis of the compounds present in peaks 1–8 is given in diagrammatic form in Figure 5. Because not all peaks led to a sample containing a single pure compound, we have compiled Table 1 to summarize the relationship between the compounds present in the analytical HPLC peaks and those identified in the purified samples isolated after silica gel chromatography and subsequent preparative HPLC (*vide infra*).

**Table 1:** Relationship between Analytical HPLC Chromatogram Peak, Sample Number, and the Compounds in Samples Which Are the Basis of Further Discussion in This Paper

HPLC peak	sample no.^a^	compds present in sample
Figure 3B, peak 1	1	**1a**, **1b**, **1c**
Figure 3B, peak 2	2	**2**
Figure 3B, peak 3	3	**3**
Figure 3B, peak 4	4	**4**
Figure 3B, peak 5	5	**5a**, **5b**
Figure 3B, peak 6	6	**6**
Figure 4, peak 7	7	**7**
Figure 4, peak 8	8	**8**

a Isolated by preparative HPLC.

### Isolation and Structural Characterization of New Glycosylated Fasamycin Congeners

To provide sufficient material for purification and structure elucidation, we twice scaled up (6 L) fermentation of *Sacch. erythraea* Δ*ery*_215GΔ*forJ* on SFM agar. In each case, after 10 days of growth the agar was chopped up and extracted by soaking in ethyl acetate. The resulting crude extracts were fractionated first by flash chromatography over silica gel and then by preparative HPLC over C_18_ reverse-phase silica gel, and the results were analyzed by LCMS. This led to eight samples corresponding to peaks 1–8 (Table 1) for which LCMS/MS data were obtained. For all compounds the UV spectra were consistent with that of a fasamycin chromophore (λ_max_ at 247, 289, 352, and 422 nm (Figure S1, Supporting Information)).

With the isolated samples 1–8 in hand, a full suite of 1D (^1^H and ^13^C) and 2D (COSY, NOESY, HSQC-edited, HSQC-coupled, HMBC, and ROESY) NMR spectra were recorded for each sample, and we retained a small amount of each sample for subsequent antibacterial assays. We then used high performance anion exchange chromatography with pulsed amperometric detection (HPAEC-PAD) analysis to identify the constituent saccharide units from the remaining material by comparison with authentic standards to aid interpretation of the NMR spectra and elucidation of chemical structures.^12^ This was done after the NMR spectra were acquired because sample hydrolysis is required to liberate the carbohydrates for HPAEC-PAD. Samples were hydrolyzed by heating in 1.0 M aqueous trifluoroacetic acid, and the resulting polar monosaccharides were separated from the lipophilic fasamycin aglycone with the use of a C_18_ SPE cartridge. The identity of the fasamycin aglycone for samples 1–4 was confirmed by LCMS in comparison with an authentic fasamycin C standard. The identity of the fasamycin aglycone for sample 5 was confirmed by NMR analysis, although there was insufficient material from sample 6 for NMR. Aglycone analysis was not performed after hydrolysis of samples 7 and 8.

**Figure 6:**
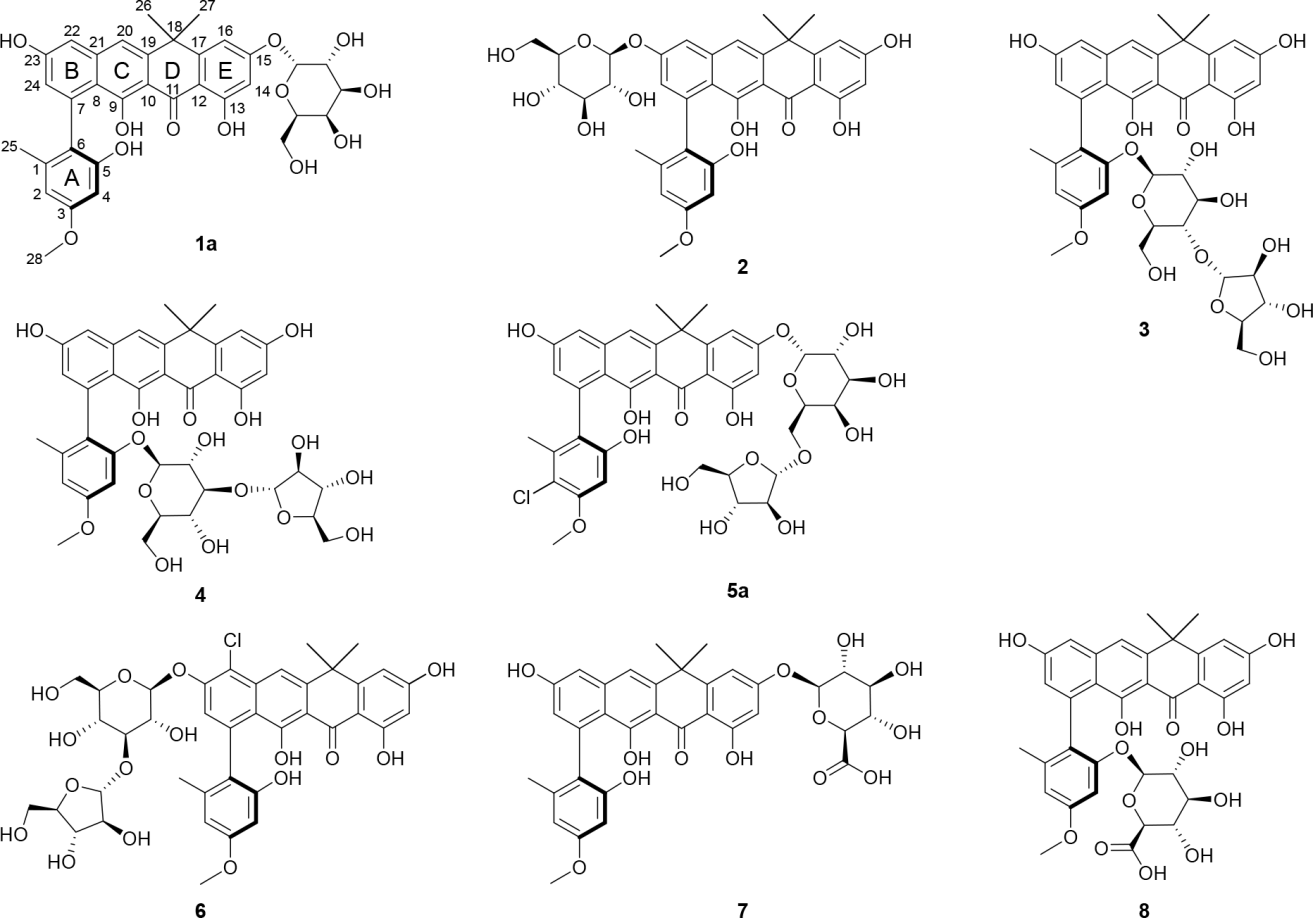
Chemical structures of the new fasamycin congeners identified in this study, deduced by NMR, LCMS, and HPAEC-PAD.

Analysis of chemical shifts and the coupling constants of the fasamycin aglycone in the ^1^H NMR spectra of samples 1–8 revealed close similarities to the ^1^H NMR spectra of the reported fasamycin C or the monochlorinated fasamycins D and J.^1,11^ However, as a result of glycosylation, some chemical shifts displayed noticeable changes. Those changes, together with ROESY and HMBC (when available) data, were used to elucidate the regiochemistry of *O*-glycosylation; fasamycin aglycones have five hydroxy groups potentially available to form *O*-glycosidic linkages. Furthermore, the ring size and anomeric configuration of the saccharide units were established using characteristic carbohydrate *J* couplings and the chemical shifts of anomeric signals in the ^1^H and ^13^C NMR spectra.^13^ While relative configurations of glycosyl residues on compounds in samples 1–8 were determined with great confidence, the absolute configuration was not determined. As such we have chosen to represent the carbohydrate units with the configurations most commonly found in bacterial natural products (here d-Glc, d-Gal, d-GluA, and d-Ara) (Figure 6).^14^

Compounds **1a**, **1b** and **1c** were obtained as a mixture, named sample 1, which was a yellow amorphous solid. LCMS/MS initially suggested this to be a mixture of two compounds (Figure S2, Supporting Information), but it was subsequently shown to be mixture of three species, **1a**–**1c**, that could not be further separated chromatographically (*vide infra*). High resolution electrospray ionization mass spectrometric (HRESIMS) analysis indicated the presence of species with molecular formulas C_34_H_34_O_12_ (**1a**: *m*/*z* 635.2125, [M + H]^+^) and C_39_H_42_O_16_ (**1b**, **1c**: *m*/*z* 767.2545, [M + H]^+^). MS/MS analysis of the ion *m*/*z* 767.2545 revealed two fragments corresponding to the loss of 132 and 294 Da from the intact mass (see Figure 5 for schematic representation and Figure S4 in the Supporting Information for MS spectral data). These losses were attributed to the cleavage of glycosidic residues, in this case a single terminal pentose (−132 Da) or a hexose and one pentose (−294 Da) leading to fragments *m*/*z* 635.2145 and 473.1585, respectively. The character of MS/MS fragmentation for ion *m*/*z* 767.2545, which showed no losses of hexose alone, was consistent with an aglycone modified with a disaccharide having a terminal pentose and a proximal hexose. MS/MS fragmentation of the molecular ion with *m*/*z* 635.2125 showed a main fragment with *m*/*z* 473.1593 resulting from the loss of 162 Da corresponding to a single hexose unit (Figure S3, Supporting Information). The fragment ion with *m*/*z* 473.16 observed for all of **1a**–**1c** correlates with the *m*/*z* of a fasamycin C ([M + H]^+^) adduct. Taken together, these analyses suggested that these compounds are *O*-glycosylated fasamycin C derivatives with **1b** and **1c** glycosylated by a disaccharide (a proximal hexose and a terminal pentose) and **1a** glycosylated by a monosaccharide (a hexose). HPAEC-PAD analysis of hydrolyzed sample 1 (Figure S14, Supporting Information) showed three major carbohydrate peaks which corresponded to glucose (Glc), galactose (Gal), and arabinose (Ara) standards. The aglycone liberated by acid hydrolysis was confirmed to be fasamcyin C by LCMS comparison with an authentic standard (Figure S22, Supporting Information).

The major component **1a** of sample 1 yielded a ^1^H NMR spectrum with resonances for the aglycone component consistent with those for fasamycin C (Figures S25–S31, Table S3, Supporting Information).^1^ The glycosidic region of this spectrum contained seven signals consistent with the hexose deduced by MS/MS. These signals were assigned using COSY spectroscopy and correlated with ^13^C chemical shifts using HSQC spectroscopy. The chemical shift of the ^13^C anomeric signal (99.2 ppm) and the small ^3^*J*_1Hex_,_2Hex_ coupling constant (3.3 Hz) suggested a pyranose with a 1,2-*cis*-configuration. The latter was confirmed by the ^1^*J*_C1Hex_,_H1Hex_ coupling constant of 173 Hz, a value typical for 1,2-*cis*-glycopyranosides.^15^ Comparison of the ^1^H and ^13^C NMR spectra for **1a** with the literature data of various glycopyranosides allowed us to define the glycosidic component as 1,2-*cis*-galactopyranoside, consistent with the HPAEC-PAD analysis. ROESY and HMBC spectra were then used to determine the regiochemistry of glycosylation; the signal for H1Gal of the galactose residue (5.66 ppm) in the ROESY spectrum had cross-peaks with H14 and H16 indicating glycosylation at O15. Further evidence for this was obtained from a HMBC cross-peak between H1Gal and C15 of the aglycone. Taken together, compound **1a** was determined to be 15-*O*-α-galactopyranosyl-fasamycin C (Figure 6).

Close examination of the anomeric region (5.7–5.2 ppm) of the ^1^H NMR spectra of sample 1 revealed low intensity peaks which originate from the pentosyl-hexoside species identified by LCMS/MS. Thus, a singlet at 5.39 ppm was tentatively assigned to a 1,2-*trans*-arabinofuranosyl residue while doublets at 5.12 and 5.06 ppm, with characteristic ^3^*J*_1Hex,2Hex_ = 7.8 Hz couplings, belong to 1,2-*trans*-hexopyranosyl residues. However, low intensities and signal overlap prevented identification and assignment of all carbohydrate signals of **1b** and **1c**. Thus, the exact nature of the disaccharide units in **1b** and **1c** and the mode of their attachment to fasamycin C remain to be determined. On the basis of the MS/MS and ^1^H NMR data, in conjunction with the HPAEC-PAD analysis, we propose the structures as a fasamycin C aglycone with a proximal hexose moiety that is either glucose or galactose and a terminal arabinose moiety.

Compound **2** was obtained as an amorphous yellow solid. HRESIMS indicated the presence of a species with the molecular formula C_34_H_34_O_12_ (*m*/*z* 635.2111, [M + H]^+^). MS/MS analysis (Figure 5 and Figure S5, Supporting Information) showed fragments with a loss of 162 Da (for a hexose) to give an aglycone fragment with an *m*/*z* of 473.1589 ([M + H]^+^), consistent with fasamycin C. HPAEC-PAD analysis of the hydrolyzed sample showed only one carbohydrate peak, which corresponded to glucose (Figure S15, Supporting Information), and the aglycone produced by acid hydrolysis was confirmed to be fasamycin C by LCMS comparison with an authentic standard (Figure S22, Supporting Information).

Inspection of the ^1^H NMR spectra of **2** (Figures S32–S37, Table S4, Supporting Information) showed proton chemical shifts with high similarities to those for fasamycin C with the exception of the H22 and H24 signals.^1^ Similarly, most of the ^13^C NMR chemical shifts of the fasamycin C backbone of **2** were within 0.3 ppm from the corresponding signals in the ^13^C NMR spectra of fasamycin C with the largest difference (0.7–1.4 ppm) being observed for the resonances of ring B. The structure of the hexose moiety (determined as glucose by HPAEC-PAD analysis) was assigned as a 1,2-*trans-*glucopyranose based on a ^13^C chemical shift of 101.4 ppm for C1Glc in conjunction with a ^3^*J*_H1Glc,H2Glc_ value of 7.4 Hz. The regiochemistry of glycosylation was assigned as O23 from an HMBC cross-peak between the anomeric proton of the glucose moiety (H1Glc) and C23 of the aglycone in the HMBC spectra, in conjunction with a through-space interaction between H1Glc and H22 and H24 of the aglycone in the ROESY spectra. Taken together, compound **2** was determined to be 23-*O*-β-glucopyranosyl-fasamycin C (Figure 6).

Compound **3** was obtained as a yellow amorphous solid. HRESIMS indicated a molecular formula of C_39_H_42_O_16_ (*m*/*z* 767.2531, [M + H]^+^). MS/MS analysis of **3** (Figure 5 and Figure S6, Supporting Information) revealed two diagnostic fragments with an *m*/*z* of 635.2132 corresponding to a loss of 132 Da suggesting the loss of a pentose and an *m*/*z* of 473.1592 corresponding to a loss of 294 Da suggesting the loss of a disaccharide (pentose plus hexose). We did not observe an ion for the loss of a hexose without a loss of the pentose by MS/MS, and together this data suggested a proximal hexose with a terminal pentose. HPAEC-PAD analysis of the hydrolyzed sample showed two major carbohydrate peaks which corresponded to glucose and arabinose in a ca. 1:1 ratio (Figure S16, Supporting Information). The aglycone produced by acid hydrolysis was confirmed to be fasamycin C by comparison with an authentic standard by LCMS, consistent with the MS/MS data (Figure S22, Supporting Information).

Analysis of COSY and HSQC NMR spectra (Figures S38–S42, Table S5, Supporting Information) established the disaccharide consisted of a terminal 1,2-*trans*-arabinofuranose residue (a doublet for H1Ara at 5.40 ppm with *J*_1Ara,2Ara_ = 1.8 Hz) attached to O4 of a proximal 1,2-*trans*-glucopyranose (H1 at 4.97 ppm with *J*_1Glc,2Glc_ = 7.9 Hz). The 1,4-interglycosidic linkage was supported by the ROESY spectra in which correlations between H1Ara of the arabinofuranose and H4Glc of the glucopyranose were observed, in addition to an HMBC cross-peak between H1Ara and C4Glc. The regiochemistry of glycosylation of fasamcyin C by the disaccharide was assigned by an HMBC cross-peak between H1Glc and C5, as well as a through-space interaction between H1Glc and H4 in the ROESY spectra. In addition, inspection of the ^1^H NMR spectra of **3** revealed all characteristic resonances of a fasamycin C aglycone with minimal deviation (Δδ < 0.04 ppm) from those of fasamycin C except for H2 and H4 which showed ∼0.35 ppm downfield shifts suggesting glycosylation of O5. Taken together, compound **3** was determined to be 5-*O*-(4-*O*-(α-arabinofuranosyl)-β-glucopyranosyl)-fasamycin C (Figure 6).

Compound **4** was obtained as a yellow amorphous solid. HRESIMS indicated a molecular formula of C_39_H_42_O_16_ (*m*/*z* 767.2541, [M + H]^+^). MS/MS analysis of **4** (Figure 5 and Figure S7, Supporting Information) revealed two diagnostic fragments with an *m*/*z* of 635.2130 corresponding to a loss of 132 Da suggesting a pentose and an *m*/*z* of 473.1592 corresponding to a loss of 294 Da suggesting a disaccharide (pentose plus hexose). We did not observe an ion for the loss of a hexose without the loss of the pentose by MS/MS, and together these data suggested a proximal hexose with a terminal pentose. HPAEC-PAD analysis of the hydrolyzed sample showed two major carbohydrate peaks which corresponded to glucose and arabinose in a ca. 1:1 ratio (Figure S17, Supporting Information). The aglycone produced by acid hydrolysis was found to be fasamycin C by comparison with an authentic standard by LCMS (Figure S22, Supporting Information). In a similar fashion to **3**, the presence of a broad singlet at 5.41 ppm (H1Ara) and a doublet with ^3^*J*_1Glc,2Glc_ = 7.8 Hz at 5.04 ppm (H1Glc) meant the disaccharide consisted of a terminal 1,2-*trans*-arabinofuranose moiety with a proximal 1,2-*trans*-glucopyranose (Figures S43–S48, Table S6, Supporting Information). The connectivity in the disaccharide was determined by a cross-peak between the anomeric proton of arabinose (H1Ara) and C3 of the glucose (C3Glc) moiety in the HMBC spectra. This was confirmed by the low field shift of C3Glc that arises due to O3 glycosylation (cf. δ C3 82.4 in **4** and δ C3 78.04 in **3** in ^13^C NMR spectra), and by through-space interactions between H1Ara and H3Glc observed in the ROESY spectra. From the ROESY spectra it was apparent that the glucosyl residue is attached to O5 of the aglycone based on a clear cross-peak between H1Glc and H5 of the aglycone, and this was further supported by an HMBC cross-peak between H1Glc and C5. Taken together, compound **4** was determined to be 5-*O*-(3-*O*-(α-arabinofuranosyl)-β-glucopyranosyl)-fasamycin C (Figure 6).

Compounds **5a** and **5b** were obtained as a mixture, named sample 5, which was a yellow amorphous solid. By utilizing extracted ion chromatograms, we showed by LCMS that sample 5 contained two essentially coeluting species (Figure S8, Supporting Information). HRESIMS of the marginally earlier eluting species **5a** indicated a molecular formula of C_39_H_41_ClO_16_ (*m*/*z* 801.2151, [M + H]^+^) and an isotope pattern characteristic of a singly chlorinated species. MS/MS analysis of **5a** (Figure 5 and Figure S9, Supporting Information) revealed two diagnostic fragments with an *m*/*z* of 669.1738 corresponding to a loss of 132 Da suggesting the loss of a pentose and an *m*/*z* of 507.1202 corresponding to loss of 294 Da suggesting the loss of a disaccharide (pentose plus hexose). We did not observe an ion for the loss of a hexose without the loss of the pentose by MS/MS, and together this data suggested a proximal hexose with a terminal pentose.

HRESIMS of the later eluting species **5b** indicated a molecular formula of C_34_H_33_ClO_12_ (*m*/*z* 669.1731, [M + H]^+^) and displayed an isotope pattern characteristic of a singly chlorinated species. MS/MS analysis of **5b** (Figure 5 and Figure S10, Supporting Information) revealed a diagnostic fragment with an *m*/*z* of 507.1200 corresponding to a loss of 162 Da suggesting the loss of a hexose. These observations suggested that sample 5 contains two compounds that both comprise a monochlorinated fasamycin aglycone (therefore either fasamycin D or J), one which is glycosylated with a disaccharide composed of a proximal hexose and a terminal pentose and the other a single hexose. HPAEC-PAD analysis of hydrolyzed sample 5 showed three carbohydrate peaks which corresponded to galactose, glucose, and arabinose in equal ratios (Figure S18, Supporting Information). Low resolution mass spectrometry (LRMS) of the aglycone produced by acid hydrolysis of sample 5 showed an *m*/*z* of 507 ([M + H]^+^) and an isotope pattern which corresponded to a monochlorinated species, indicative of either fasamycin D or J.^1,11^ As we did not have authentic standards of fasamycin D or J to hand, we recorded the ^1^H NMR of this fasamycin, which matched that of the published spectra for fasamycin J (Table S2, Supporting Information).^11^

The aromatic region of the ^1^H NMR spectra for sample 5 was dominated by one set of signals which, in combination with the data above, led us to propose that the site of glycosylation is the same for both **5a** and **5b** (Figures S49–S53, Table S7, Supporting Information). The anomeric region of the ^1^H NMR spectra showed two major resonances and a third minor resonance. The first major anomeric signal at 5.65 ppm displayed a small coupling constant (^3^*J*_1Hex,2Hex_ = 3.3 Hz) and was correlated with a ^13^C signal at 98.8 ppm in the HSQC spectra. Consequently, this peak can be attributed to an anomeric proton of a 1,2-*cis*-galactopyranosyl residue. This signal also displayed clear cross-peaks with H14 and H16 in the ROESY spectra, indicating glycosylation at O15 of the aglycone. The second main anomeric signal was a singlet resonating at 5.40 ppm that was correlated with a signal at 109.1 ppm in the HSQC spectra. These chemical shifts are characteristic for furanosides and can be assigned to a 1,2-*trans*-arabinofuranosyl residue. The signal at 5.40 ppm has no detectable cross-peaks with any protons of fasamycin in the ROESY spectra but correlated with a resonance at 3.76 ppm which, based on the HSQC-edited spectra, can be assigned to a methylene group in the carbohydrate region of the ^1^H NMR, and they are therefore most likely to be the H6Gal protons of the galactopyranose residue. Taking all the observations together, we determined the structure of **5a**, the main component of sample 5, to be 15-*O*-(6-*O*-(α-arabinofuranosyl)-α-galactopyranosyl)-fasamycin J.

The third low intensity peak found in the anomeric region of ^1^H NMR spectra of sample 5 had a resonance at 5.12 ppm and coupling of *J*_1Hex,2Hex_ = 7.9 Hz. This was connected to a carbon signal resonating at 100.7 ppm as determined from the HSQC spectra. These chemical shifts are consistent with the structure of a 1,2-*trans*-*O*-glucopyranoside, the third sugar observed in the HPAEC-PAD analysis. In the ROESY spectra the resonance assigned to H1Glc of the 1,2-*trans*-*O*-glucopyranoside showed a through-space correlation with a pair of weak signals for aromatic protons at 6.54 and 6.94 ppm, and these signals were assigned to the minor component of sample 5 (**5b**). When combined with the HMBC and HSQC data, and observations noted above, we assign the aglycone of **5b** as the same as **5a** (cf. fasamycin J). We were unable to fully assign the NMR data for **5b**. However, when combining the NMR data with that from MS/MS and HPAEC-PAD analysis, we suggest a tentative structure of **5b** as 15-*O*-(6-*O*-(α-arabinofuranosyl)-β-glucopyranosyl)-fasamycin J.

Compound **6** was obtained as a yellow amorphous solid, and HRESIMS indicated a molecular formula of C_39_H_41_ClO_16_ (*m*/*z* 801.2145, [M + H]^+^) and an isotope pattern characteristic of a singly chlorinated species. MS/MS analysis of **6** (Figure 5, Figure S11, Supporting Information) revealed two diagnostic fragments with an *m*/*z* of 669.1744, corresponding to a loss of 132 Da suggesting a pentose, and an *m*/*z* of 507.1201 corresponding to loss of 294 Da suggesting a disaccharide (pentose plus hexose). We did not observe an ion for the loss of a hexose without the loss of the pentose by MS/MS, and together this data suggested a proximal hexose with a terminal pentose. HPAEC-PAD analysis of the hydrolyzed sample showed two major carbohydrate peaks which corresponded to glucose and arabinose in a ca. 1:1 ratio (Figure S19, Supporting Information). The aglycone produced by acid hydrolysis of sample **6** was analyzed by LRMS and had an *m*/*z* of 507 ([M + H]^+^) and an isotope pattern consistent with a singly chlorinated species. Together with the UV absorbance spectrum, this suggested the aglycone was monochlorinated fasamycin D or J. The aglycone peak from sample 6 had a different retention time subtly different from that from sample 5 (Figure S23, Supporting Information), indicating it was not fasamycin J. Unfortunately, the quantity of aglycone recovered after hydrolysis of **6** was insufficient to obtain ^1^H NMR spectra. Careful analysis of the HMBC and ROESY NMR spectra of **6** showed no correlations which can be attributed to interactions with H22 as observed in fasamycin C derivatives, indicating that this proton is absent in the structure of **6**. Further comparison with the published NMR for fasamycin D suggested chlorination at C22 and allowed us to assign the aglycone as fasamycin D.^1^

The anomeric region of the ^1^H NMR showed two distinct signals (Figures S54–S58, Table S8, Supporting Information). Analysis of COSY and HSQC spectra established a disaccharide consisting of a terminal 1,2-*trans*-arabinofuranose residue (a singlet for H1Ara at 5.37 ppm) attached to O3 of a proximal 1,2-*trans*-glucopyaranose (H1Glc at 5.27 ppm with ^3^*J*_1Glc,2Glc_ = 7.8 Hz). The 1,3-glycosidic linkage was supported by cross-peaks in the ROESY spectra between H1Ara of the arabinofuranose and H3Glc of the glucopyranose. The regiochemistry of fasamycin glycosylation was determined by a correlation between H1Glc and H24 of ring A in the ROESY spectra. Taken together with the MS/MS and HPAEC-PAD data, the structure of **6** was determined as 23-*O*-(3-*O*-α-arabinofuranosyl)-β-glucopyranosyl)-fasamycin D.

Compound **7** was obtained as a yellow amorphous solid, and HRESIMS showed a parent ion with *m*/*z* 649.1904 indicating a molecular formula of C_34_H_32_O_13_. In-source fragmentation of an infused sample (Figure 5, Figure S12, Supporting Information) gave a major fragment with an *m*/*z* of 473.1581 ([M + H]^+^), indicating an aglycone formula of C_28_H_24_O_7_ which is consistent with fasamycin C. The loss of 176 Da resulting from that fragmentation is indicative of a hexauronic acid, and the HPAEC-PAD analysis of the hydrolyzed sample gave a single carbohydrate peak which was identified as glucuronic acid (Figure S20, Supporting Information).

NMR spectra of **7** were consistent with the presence in its structure of an aglycone moiety of fasamycin C since proton and carbon resonances reported for fasamycin C^1^ can be identified in ^1^H and ^13^C NMR spectra of **7** (Figures S59–S64, Table S9, Supporting Information). The only noticeable difference (ca. 0.3 ppm downfield shift) between the ^1^H NMR spectra of **7** and fasamycin C was in the position of H14 and H16 that suggested the glycosylation of O15 in ring E of fasamycin C. Two-dimensional HSQC and HMBC NMR spectroscopies confirmed the assignment of the ^1^H and ^13^C NMR spectra. The carbohydrate region of the ^13^C spectra showed five resonances consistent with the glucopyranosiduronic acid (GlcA) moiety; the resonance of the carboxyl group was not detected but a H5GlcA–C6GlcA correlation was observed in the HMBC spectra. The large ^3^*J*_1GlcA,2GlcA_ coupling constant (7.5 Hz) was indicative of a 1,2-*trans*-glucuronide. The position of glycosylation was assigned as O15 based on a HMBC correlation between H1GlcA and C15 of the aglycone and the observation of NOESY correlations between H1GlcA and the H14 and H16 protons of the aglycone. On the basis of the combined data, the structure of **7** was determined to be 15-*O*-(β-glucopyranosyluronic acid)-fasamycin C.

Compound **8** was obtained as a yellow amorphous solid, and HRESIMS gave a parent ion with *m*/*z* 649.1920, indicating a molecular formula of C_34_H_32_O_13_ and suggesting a structural isomer of **7**. In-source fragmentation (Figure 5, Figure S13, Supporting Information) of an infused sample gave a major fragment with an *m*/*z* of 473.1589 ([M + H]^+^) consistent with a fasamycin C aglycone. Again, as for **7**, the loss of 176 Da is indicative of a hexuronic acid and HPAEC-PAD analysis of the hydrolyzed sample showed a single carbohydrate peak which was identified as glucuronic acid (Figure S21, Supporting Information).

There were 11 proton resonances in the ^1^H and COSY NMR consistent with NMR data for the fasamycin C aglycone (Figures S65–S70, Table S10, Supporting Information).^1^ The structure of the aglycone was confirmed by analysis of the HSQC and HMBC spectra which also allowed the assignment of all ^1^H and ^13^C NMR resonances corresponding to fasamycin C. We did note however that the chemical shift of H4 (δ 6.69) was shifted 0.4 ppm downfield compared to unmodified fasamycin C, suggesting glycosylation may be at O5 of ring A. The carbohydrate region of the ^1^H NMR spectra of **8** showed resonances that were (like **7**) consistent with positions 1–5 of a glucopyranosiduronic acid moiety, and the anomeric configuration was assigned as 1,2-*trans* based on the ^3^*J*_1GlcA,2GlcA_ coupling constant of 7.3 Hz. The six carbon resonances of this glucuronic acid moiety, including the C6 carboxyl at 176.6 ppm, were assigned using HSQC and HMBC data. Correlation between H1GlcA and C5 of the aglycone observed in the HMBC spectra provided support for O5 as the site of glycosylation. That was confirmed by a correlation between H1GlcA and H4 of the aglycone in the ROESY spectra. On the basis of the combined data the structure of **8** was determined to be 5-*O*-(β-glucopyranosyluronic acid)-fasamcyin C.

### Bioactivity of Glycosylated Fasamycin Congeners

During preparation of this paper two related glycosylated naphthacemycins (D_1_ and D_2_) were isolated from *Streptomyces* sp. N12W1565, and both compounds were reported to exhibit moderate antibacterial activity against MRSA, *Bacillus subtilis*, *E. coli*, and *Pseudomonas aeruginosa*.^16^ On this basis we tested samples 1–6 against a panel of indicator strains including *B. subtilis*, *Staph. aureus*, and *E. coli* using spot-on-lawn assays. Surprisingly, none of samples 1–6 exhibited any antibacterial activity up to 120 μg/mL against the strains tested, whereas different positive controls did (Figure S71, Supporting Information). We hypothesize that either glycosylation of the fasamycin C, D, or J backbone abolishes their previously demonstrated bioactivity^1,11^ or that the concentrations tested were not sufficient to demonstrate activity. Samples 7 and 8 were not tested as all the material was consumed running the HPAEC-PAD analysis.

## Conclusions

We set out to engineer formicamycin and fasamycin production by expression of the *for* BGC in two heterologous hosts, *S. coelicolor* M1146 and *Sacch. erythraea* Δ*ery*. *S. coelicolor* M1146 has been widely used as a heterologous expression host due to its excellent growth characteristics, its genetic tractability, and the wide range of plasmids that can be used for its modification.^17,18^ This strain has also been engineered to remove its capacity to biosynthesize four of its endogenous specialized metabolites, meaning carbon flux can be diverted for use by heterologously expressed pathways, and that the fermentation profile is simplified for the chemical analysis of extracts and the isolation of new molecules. Although not as fast growing or amenable to genetic modification as *S. coelicolor* M1146, *Sacch. erythraea* Δ*ery* has been similarly engineered through specific deletion of the entire erythromycin BGC (other than the immunity gene *ermE*) and lacks the ability to make its endogenous antibiotic erythromycin. It has been used widely to express gene cassettes for deoxysugar biosynthesis and the subsequent biotransformation of exogenously added aglycones to produce new glycosylated products.^19,20^ Desired outcomes of this work were to understand if heterologous expression of the *for* BGC could lead to elevated titers of fasamycins and formicamycins for scale-up and isolation and to lay the foundation for biosynthetic engineering studies to generate new analogues of these exciting antibiotics.

As described herein, heterologous expression of the *for* BGC cloned on a phage-derived artificial chromosome (named pESAC-13_215G) did not lead to the production of either fasamycins or formicamycins unless the gene *forJ*, which encodes for a MarR-type repressor, was deleted (leading to pESAC-13_215GΔ*forJ*). Deletion of *forJ* in the native producer *S. formicae* has been shown to both increase titers and enable production in liquid culture.^7^ Unfortunately, while the heterologous expression of pESAC13_215GΔ*forJ* in both heterologous hosts did indeed lead to the production of fasamycins and formicamycins, the titers of these compounds were much lower relative to the native producer in which *forJ* was deleted (*S. formicae* Δ*forJ*). It is well-documented that the heterologous expression of BGCs often fails or leads to low levels of compound production, and while the reasons for this are not clear, likely possibilities include a lack of correspondence between regulatory networks, insufficient precursor supply, and the requirement for chaperones or immunity factors that are present only in the native genome.

LCMS/MS analysis indicated that *Sacch. erythraea* Δ*ery*_215GΔ*forJ* produced low levels of new congeners which appeared to be novel glycosides of known fasamycin aglycones. To access these compounds, we performed upscaled fermentation and, following solvent extraction and multiple rounds of chromatography, obtained purified samples containing these molecules. Through a combination of careful LCMS/MS, HPAEC-PAD, and NMR analysis, we showed that these new compounds are fasamycin congeners modified at different phenolic groups with either a monosaccharide (glucose, galactose, or glucuronic acid) or a disaccharide comprised of a proximal hexose (either glucose or galactose) with a terminal arabinose moiety. In two cases the sample contained two or three closely related glycosylated variants that could not be resolved chromatographically and which were available in extremely low levels. These compounds did not show any antibacterial activity when tested against a panel of lab indicator strains (both Gram-positive and Gram-negative); this contrasted with the recently reported glycosylated naphthacemycins D_1_ and D_2_ isolated from *Streptomyces* sp. N12W1565 which were reported to inhibit the growth of both Gram-positive and Gram-negative bioindicator strains.^16^

The glycosyltransferase NatY, reportedly responsible for the biosynthesis of naphthacemycins D_1_ and D_2_, has been identified from *Streptomyces* sp. N12W1565, and its activity has been investigated.^16^ We thus carried out bioinformatics analysis and identified a gene encoding a homologue of NatY (with 48% identity) in the genome of *Sacch. erythraea* NRRL2338,^21^ the putative gene product of which (Accession No. PFG99483.1) belongs to the Yjic superfamily of flavonoid glycosyltransferases. Two homologues of NatY (with 55 and 49% identities, Accession Nos. QKN67391.1 and QKN6790.1, respectively) are also encoded in the *S. coelicolor* genome and belong to the same Yjic superfamily. The lack of a significantly similar homologue in the *S. formicae* KY5 genome perhaps explains why no glycosylated fasamycin and/or formicamycin congeners could be identified from the native producer. The identification of these enzymes, alongside NatY, provides the basis for future glycodiversification of the fasamycin and formicamycin skeletons and associated structure–activity relationship investigations, including activity against cancer cell lines.

## Experimental Section

### General Experimental Procedures

Solvents used for extractions and HPLC analysis were bought from Fisher Scientific. Reagents and chemicals were purchased from Alfa-Aesar and Sigma-Aldrich (Merck). NMR spectra (1D and 2D) were recorded in CD_3_OD at 298 K on a Bruker Neo 600 MHz spectrometer equipped with 5 mm TCI CryoProbe. Two-dimensional ^1^H–^1^H-COSY, ^1^H–^13^C-HSQCed, HMBC, and ROESY experiments were performed using standard pulse sequences from the Bruker Topspin library. Data were processed using Topspin 4.1.4 and MestReNova 14.2.3 software, and spectra were calibrated to the residual solvent signals (δ_H/C_ 3.31/49.00 ppm). For samples 3, 5, and 6, ^13^C NMR chemical shifts were determined from HMBC and HSQC spectra. *E. coli* strains were maintained on solid LB agar with appropriate selection at 37 °C. The strains and plasmids used in this study are described in Tables 2 and 3, respectively.

**Table 2:** Strains Used in This Study

strain	description/genotype	plasmid	resistance	ref
*E. coli* TOP10	F^–^*mcrA* Δ(*mrr-hsd*RMS*mcrBC*) Φ80*lacZ*Δ*M15* Δ*lacX74 recA1 araD139* Δ(*ara leu*)7697 *galU galK rpsL* (StrR) *endA1 nupG*			Invitrogen
*E. coli* DH5α	Δ(*argF-lac*)*169*, φ*80dlacZ58*(*M15*), Δp*hoA8*, *gln X44*(*AS*), *deoR481*, *rfbC1*, *gyrA96*(*NalR*), *recA1*, *endA1*, *thiE1andhsdR17*			Invitrogen
*E. coli* DH10B	F^–^*mcrA* Δ(*mrr-hsdRMS-mcrBC*) φ80*lacZ*ΔM15 Δ*lacX74 recA1 endA1 araD139* Δ(*ara-leu*)7697 *galU galK* λ^–^*rpsL*(Str^R^) *nupG*			Invitrogen
*E. coli* BW25113	λ^–^, Δ(*araD-araB*)567, Δ*lacZ4787*(*::rrnB-4*), *lacIp4000*(*lacIQ*), *rpoS369*(Am), *rph-1*, Δ(*rhaD-rhaB*)568, *hsdR514*	pIJ790	Cml	(22)
*E. coli* ET12567	*E. coli* ET12567 is methylation deficient (Δ*dcm* Δ*dam*)		Cml	(23)
*S. coelicolor* M1146	host strain [Δ*act* Δ*red* Δ*cpk* Δ*cda*]			(18)
*S. coelicolor* M1146_215G	M1146 + *for* BGC	pESAC-13_215G		this work
*S. coelicolor* M1146_215GΔ*forJ*	M1146 + *for* BGC Δ*forJ* (derepressed)	pESAC-13_215GΔ*forJ*		this work
*Sacch. erythraea* Δ*ery*	Δ*ery*			Isomerase Therapeutics (Cambridge, U.K.)
*Sacch. erythraea* Δ*ery*_215G	+ *for* BGC	pESAC-13_215G		this work
*Sacch. erythraea* Δ*ery*_215GΔ*forJ*	+ *for* BGC Δ*forJ* (derepressed)	pESAC-13_215GΔ*forJ*		this work

**Table 3:** Details of Plasmids Used in This Study

plasmid	description	resistance	ref
pR9604	pUB307 derivative	Carb	(24)
pIJ773	– *aac(3)IV oriT bla*	Apr	(10)
pUZ8002	RK2 derivative with a mutation in *oriT*	Kan	(25)
pIJ790	λ-RED (*gam, bet, exo*), *cat*, *araC*, *rep101*^ts^	Cml	(10)
pESAC-13_215G	*aphII*, *tsr*	Kan/Tsr	BioS&T and (1, 7)
pESAC-13_215G Δ*forJ*	*aphII*, *tsr*, *forJ* replaced with apramycin resistance gene amplified from pIJ773	Kan/Tsr/Apr	this work

### Generation of Heterologous Expression Strains

To generate PAC 215GΔ*forJ*, *E. coli* ReDirect PCR targeting was used to replace the *forJ* coding region in pESAC-13_215G with an apramycin resistance gene in *E. coli* using Lambda RED.^10^ The apramycin resistance gene was PCR amplified from pIJ773 with flanks complementary to the 3′ and 5′ ends of the *forJ* coding region (forward: CGG TCT CGA AGC ACG TCA CAG CAG AGG TGA GCG AAC ATG GCT CAC GGT AAC TGA TGC CG; reverse: GCG GAC CGT GCC TAG GCC CCG CCG GGA ACG ACC GCG TCA TGT AGG CTG GAG CTG CTT C) and purified using the Qiaquick PCR purification kit. The resulting PCR fragment was electroporated into *E. coli* BW25113/pIJ790 containing pESAC-13_215G. The expression of Lambda *red* genes was induced by the addition of l-arabinose (10 mM) to the LB growth medium (10 g/L tryptone, 5 g/L yeast extract, 10 g/L NaCl) to induce recombination between the introduced PCR fragment and pESAC-13_215G. The edited PAC was isolated by resuspension of the cell pellet from a 1 mL overnight culture of *E. coli* BW25113 + pESAC-13_215GΔ*forJ* in 100 μL of solution 1 (50 mM Tris/HCl, pH 8; 10 mM EDTA) and the addition of 200 μL of solution 2 (200 mM NaOH; 1% SDS) followed by 150 μL of solution 3 (3 M potassium acetate, pH 5.5) and mixed by inversion. After centrifugation at 20784*g* for 5 min, the supernatant was extracted in 400 μL of 1:1 phenol/chloroform, vortexed for 2 min, and centrifuged again. The upper phase was transferred to a tube containing 600 μL of 2-propanol and left on ice for 10 min to precipitate the DNA before being centrifuged again. The pellet was then washed in 200 μL of 70% ethanol, left to dry for 5 min at room temperature, and then resuspended in sterile dH_2_O. The edited PAC was electroporated into *E. coli* Top10 and isolated by the same method as above. The desired edit was confirmed using a restriction digest with *Xho*l.

The target PACs (pESAC-13_215G and the edited version pESAC-13_215GΔ*forJ*) were moved into the conjugation strain *E. coli* ET12567 by triparental mating. A 20 μL volume of each cell type (*E. coli* DH10B_pESAC13_215G or *E. coli* Top10_pESAC-13_215GΔ*forJ*, *E. coli* TOP10_pR9604 and *E. coli* ET12567 for conjugation) was spotted on top of each other in the center of an LB agar plate and incubated overnight at 37 °C. The resulting cell spot was streaked for single colonies on LB agar plates containing appropriate antibiotics to select for *E. coli* ET12567 strains containing both the cosmid and the transfer plasmid pR9604. The resulting strains (*E. coli* ET12567/pR9604/215G and *E. coli* ET12567/pR9604/215GΔ*forJ*) were grown in liquid culture overnight for conjugation into *S. coelicolor* M1146 and *Sacch. erythraea* Δ*ery* using previously described methods.^10^

### Metabolite Analysis

Metabolite analysis was performed as previously described.^7^ In brief, strains (*n* = 3) were grown on soya flour mannitol (SFM) agar (20 g/L soy flour, 20 g/L mannitol, 20 g/L agar in tap water) at 30 °C for 10 days. Agar plugs (1 cm^3^) were excised and shaken with ethyl acetate (1 mL) for 1 h before being centrifuged at 20784*g*, and 200 μL was taken for analysis. The ethyl acetate was removed under reduced pressure, and the residue was dissolved in methanol (1 mL) before being analyzed by HPLC (Agilent 1290 UHPLC). Chromatography was achieved using the following method: Phenomenex Gemini NX C18 column (150 × 4.6 mm); mobile phase A: water and 0.1% formic acid; mobile phase B: methanol. Elution gradient: *T* = 0 min, 50% B; *T* = 2 min, 50% B; *T* = 16 min, 100% B; *T* = 18 min, 100% B; *T* = 18.1 min, 50% B; *T* = 20 min, 50% B; injection volume 10 μL with UV absorbance monitoring from 190 to 600 nm.

Titers of fasamycin and formicamycins were determined by comparing peak areas from the above metabolite analysis (Agilent 1290 UHPLC) to those of standard calibration curves and correcting for the change in concentration that occurred during the extraction process. Peak area integration was conducted using LC OpenLab software, and manual integration was conducted on peaks not picked up by software due to their small sizes. Calibration curves were determined using standard solutions of fasamycin E (10, 20, 50, 80, and 200 μM) and formicamycin I (10, 20, 50, 100, 200, and 400 μM) by UV–vis absorption at 418 and 285 nm, respectively. The UV–vis absorption for each standard solution was measured three times.^7^

### Scale-Up Fermentation of *Sacch. erythraea* Δ*ery*_215GΔ*forJ*

Spores of *Sacch. erythraea* Δ*ery*_215GΔ*forJ* were spread onto SFM agar (6 L) and grown at 30 °C for 10 days. Agar was sliced into small pieces and soaked in ethyl acetate (6 L) twice over two concurrent nights. The agar was removed by filtration, and the ethyl acetate was combined and evaporated under reduced pressure to yield a crude extract. A sample of the resulting extract was resuspended in methanol and analyzed by LCMS using the metabolite analysis HPLC method to confirm that new peaks were present. The extract was fractionated using a Biotage Isolera on a SNAP Ultra 25 g silica cartridge using gradient elution and UV monitoring at 280 and 418 nm. Mobile phase A: chloroform; mobile phase B: methanol; flow rate 75 mL/min; elution started from 0% B for 1 column volume (CV), then gradient to 10% B over 12 CV, then gradient to 30% B over 1.2 CV, and then holding at 30% B for 3.1 CV This resulted in fractions comprising two major peaks at 418 nm: the first contained predominantly fasamycin C and the latter eluting fraction contained compounds **2**, **7**, and **8** (Figure 4).

A second analogous upscaled fermentation of *Sacch. erythraea* Δ*ery*_215GΔ*forJ* was performed on 6 L of SFM agar. This was extracted in the same way but using 6 L of ethyl acetate, and the crude extract was fractionated in the same way using a Biotage Isolera system. The later eluting fraction corresponding to a UV peak at 418 nm contained compounds **1**–**6**.

### Preparative HPLC Method for the Isolation of Samples **1**–**8**

Both glycosylated fasamycins containing fractions arising from the Biotage chromatography of the scale-up fermentations of *Sacch. erythraea* Δ*ery*_215GΔ*forJ* were subjected to preparative HPLC using a Thermo Scientific Dionex Ultimate 3000 HPLC system fitted with a Phenomenex Gemini-NX reversed-phase column (C_18_, 110 Å, 150 × 21.2 mm). The following conditions were applied: mobile phase A: water with 0.1% formic acid; mobile phase B: methanol; flow rate 20 mL/min; injection volume 500 μL; elution gradient: *T* = 0 min, 5% B; *T* = 2 min, 5% B; *T* = 2.5 min, 20% B; *T* = 17 min, 70% B; *T* = 19.5 min, 95% B; *T* = 21.5 min, 95% B; *T* = 23.5 min, 5% B; UV absorbance monitoring at 418 nm. Fractions were collected and analyzed by LCMS.

#### Compound **1a**

Yellow solid; UV (DAD) λ_max_ 247, 289, 352, and 422 nm; ^1^H and ^13^C NMR data in CD_3_OD, Table S3; HRESIMS *m*/*z* 635.2125 [M + H]^+^ (calcd for C_34_H_35_O_12_^+^ 635.2123, Δ = 0.3 ppm).

#### Compound **2**

Yellow solid; UV (DAD) λ_max_ 248, 283, 348, and 415 nm; ^1^H and ^13^C NMR data in CD_3_OD, Table S4; HRESIMS *m*/*z* 635.2111 [M + H]^+^ (calcd for C_34_H_35_O_12_^+^ 635.2123, Δ = −1.9 ppm).

#### Compound **3**

Yellow solid; UV (DAD) λ_max_ 247, 286, 353, and 418 nm; ^1^H and ^13^C NMR data in CD_3_OD, Table S5; HRESIMS *m*/*z* 767.2531 [M + H]^+^ (calcd for C_39_H_43_O_16_^+^ 767.2546, Δ = −2.0 ppm).

#### Compound **4**

Yellow solid; UV (DAD) λ_max_ 249, 288, 352, and 416 nm; ^1^H and ^13^C NMR data in CD_3_OD, Table S6; HRESIMS *m*/*z* 767.2541 [M + H]^+^ (calcd for C_39_H_43_O_16_^+^ 767.2546, Δ = −0.7 ppm).

#### Compound **5a**

Yellow solid; UV (DAD) λ_max_ 247, 290, 355, and 420 nm; ^1^H and ^13^C NMR data in CD_3_OD, Table S7; HRESIMS *m*/*z* 801.2151 [M + H]^+^ (calcd for C_39_H_42_ClO_16_^+^ 801.2156, Δ = −0.6 ppm).

#### Compound **6**

Yellow solid; UV (DAD) λ_max_ 247, 290, 350, and 418 nm; ^1^H and ^13^C NMR data in CD_3_OD, Table S8; HRESIMS *m*/*z* 801.2145 [M + H]^+^ (calcd for C_39_H_42_ClO_16_^+^ 801.2156, Δ = −1.4 ppm).

#### Compound **7**

Yellow solid; UV (DAD) λ_max_ 247, 288, 353, and 422 nm; ^1^H and ^13^C NMR data in CD_3_OD, Table S9; HRESIMS *m*/*z* 649.1904 [M + H]^+^ (calcd for C_34_H_33_O_13_^+^ 649.1916, Δ = −1.8 ppm).

#### Compound **8**

Yellow solid; UV (DAD) λ_max_ 248, 286, 353, and 418 nm; ^1^H and ^13^C NMR data in CD_3_OD, Table S10; HRESIMS *m*/*z* 649.1920 [M + H]^+^ (calcd for C_34_H_33_O_13_^+^ 649.1916, Δ = 0.6 ppm).

### LCMS/MS Analysis of Samples **1**–**6**

LCMS/MS analysis was performed using a Thermo QExactive LCMS instrument on a Kinetex C_18_ column (50 × 2.1 mm, 1.7 μm). LCMS/MS method, mobile phase A: water with 0.1% formic acid; mobile phase B: acetonitrile with 0.1% formic acid; flow rate 0.7 mL/min; injection volume 10 μL; elution gradient: *T* = 0 min, 30% B; *T* = 6 min, 95% B; *T* = 7.7 min, 95% B; *T* = 7.9 min, 30% B; *T* = 11 min, 30% B. The sample was analyzed in positive mode over the *m*/*z* range 200–2000 with a resolution of 35 000. The spray voltage was set to 3000 V, and the capillary temperature was 350 °C. The sheaf gas was set to 35, and the auxiliary gas was set to 10. Data dependent MS^2^ with 17 500 resolution and an isolation window of 4.0 *m*/*z* and an isolation offset of 1.0 *m*/*z* was employed with normalized collision energies of 10, 30, and 50%. The instrument was calibrated according to the manufacturer’s instructions, and the LCMS/MS data was analyzed using Thermo Scientific FreeStyle 1.7 software.

### Direct Injection HRESIMS Analysis of Samples **7** and **8**

For HRESIMS, the samples were dissolved into water + 0.1% FA/methanol (1:1) and infused into a Synapt G2-Si mass spectrometer (Waters, Manchester, U.K.) at 10 μL/min using a Harvard Apparatus syringe pump. The mass spectrometer was controlled by Masslynx 4.1 software (Waters). It was operated in resolution and positive ion mode and calibrated using sodium iodide. The sample was analyzed for 1 min with a 1 s MS scan time over the *m*/*z* range 50–1200 with 2.0 kV capillary voltage, 40 V cone voltage, and 120 °C cone temperature. Leu-enkephalin peptide (1 ng/μL, Waters) was infused at 10 μL/min as a lock mass (*m*/*z* 556.2766) and measured every 10 s. Spectra were generated in Masslynx 4.1 by combining multiple scans, and peaks were centered using automatic peak detection with lock mass correction.

### Carbohydrate (HPAEC-PAD) Analysis

Each sample 1–8 was sealed in a tube containing aqueous trifluoroacetic acid (TFA; 1.0 M, 1 mL) and heated to 105 °C overnight. The resulting sample was diluted with water (20 mL) and freeze-dried to remove all TFA. The residue was then dissolved in water/methanol (95:5, 1 mL) and passed through a C_18_ solid phase extraction cartridge (Waters, Sep-Pak Plus Short 360 mg). The cartridge was washed with water/methanol (95:5, 2 mL), and the eluted solvent was combined and dried under reduced pressure to yield the carbohydrate residues which were dissolved in water (150 μL) for HPAEC-PAD analysis. For samples 1–6 the cartridge was further washed with water/methanol (5:95, 3 mL) to elute the retained fasamycins which were used for aglycone analysis (*vide infra*). Carbohydrate analysis was performed by high performance anion exchange chromatography with pulsed amperometric detection (HPAEC-PAD) on a Dionex ICS-5000 system using a CarboPac PA20 (3 × 150 mm) analytical column coupled to a CarboPac PA20 (3 × 30 mm) guard column. For HPAEC-PAD analyses the following conditions were used: flow rate 0.25 mL/min; injection volume 5 μL; mobile phase A: 7.8 mM NaOH; mobile phase B: 156 mM NaOH with 100 mM AcONa; elution gradient: *T* = 0 min, 0% B; *T* = 30 min, 0% B; *T* = 33 min, 100% B; *T* = 55 min, 100% B; *T* = 58 min, 0% B; *T* = 72 min, 0% B. Peaks were identified by comparison with standards for hexoses of d-glucose, d-galactose, and d-mannose; for pentoses of l-arabinose, d-ribose, and d-xylose; and for uronic acids of d-glucuronic acid and d-galacturonic acid. Co-injections with standards were also performed for verification. The resulting chromatograms are shown in Figures S14–S21 (Supporting Information).

### Aglycone Analysis and Purification

LCMS analysis of aglycones produced by acid hydrolysis of samples 1–6 was performed using an Agilent 1260 system on a Phenomenex Kinetex 5 μm XB-C_18_ 100 Å (100 × 4.6 mm) column and gradient elution. The following conditions were used: flow rate 1.0 mL/min, sample injection volume 5 μL; mobile phase A: water with 0.1% formic acid; mobile phase B: acetonitrile with 0.1% formic acid; elution gradient: *T* = 0 min, 5% B; *T* = 1 min, 5% B; *T* = 11 min, 98% B; *T* = 13 min, 98% B; *T* = 13.1 min, 5% B; *T* = 15 min, 5% B; UV–vis absorbance monitoring at 254 nm. These chromatograms were compared with that for an authentic standard of fasamycin C; the resulting chromatograms are shown in Figures S22 and S23 in SI.

Following LCMS analysis the fasamycin aglycones from fractions 5 and 6 were purified by semipreparative HPLC using a Dionex 3000 ultimate system fitted with a Phenomenex Luna 5 μm C_18_ 100 Å (250 × 10 mm) column; flow rate 4.0 mL/min, solvent A: H_2_O with 0.1% formic acid; solvent B: acetonitrile with 0.1% formic acid; gradient elution: *T* = 0 min, 50% B; *T* = 3 min, 50% B; *T* = 27 min, 99% B; *T* = 32 min, 99% B; *T* = 33 min, 50% B; *T* = 38 min, 50% B; UV absorbance monitoring at 254 and 418 nm. The purified aglycones were analyzed by ^1^H NMR.

### Antibacterial Assays

Stocks of samples 1–6 were made up in 100% MeOH. Single colonies of *E. coli* 25922, *E. coli* NR698, methicillin-sensitive *Staph. aureus* ATCC 6538P, methicillin-resistant *Staph. aureus* BAA 1717. and vancomycin-sensitive *Enterococcus faecalis* OG1RF were grown in LB (*E. coli*), tryptic soy broth (*Staph. aureus*), and brain heart infusion (*Entero. faecalis*) liquid media and incubated overnight at 37 °C with shaking at 250 rpm. The resulting cultures were subcultured into fresh liquid medium and grown to exponential phase (OD_600_ 0.4–0.6). Cultures were used to inoculate soft nutrient agar, and 10 μL samples 1–6 were spotted onto each agar plate. Plates were incubated at 37 °C overnight, after which they were examined for clearance zones due to growth inhibition.

## Supporting Information Available

Spectroscopic data for compounds **1**–**8**; HPAEC-PAD carbohydrate analysis; fermentation yield and titer (PDF)
