# Supplementary material for: Heterologous Expression of the Formicamycin Biosynthetic Gene Cluster Unveils Glycosylated Fasamycin Congeners
Source: J Nat Prod. 2023 Jun 16;86(7):1677–89. doi: 10.1021/acs.jnatprod.3c00052 (PMC10391614; doi:10.1021/acs.jnatprod.3c00052)
Supplement: Supplementary file 1 — np3c00052_si_001.pdf [file np3c00052_si_001.pdf]

Supporting Information

Heterologous expression of the formicamycin biosynthetic gene cluster unveils glycosylated fasamycin congeners

Hannah P. McDonald,[1‡] Abigail Alford,[1‡] Rebecca Devine,[1] Edward S. Hems,[1] Sergey Nepogodiev,[2] Corinne J. Arnold,[1] Neil A. Holmes,[1] Martin Rejzek,[3] Anna Stanley-Smith,[4] Neil A. Holmes,[1] Matthew I. Hutchings*[1] and Barrie Wilkinson*[1]

1. Department of Molecular Microbiology, John Innes Centre, Norwich research Park, Norwich, NR4 7UH, UK
2. NMR Platform, Norwich research Park, Norwich, NR4 7UH, UK
3. Chemistry Platform, John Innes Centre, Norwich research Park, Norwich, NR4 7UH, UK
4. Isomerase, Chesterford Research Park, Cambridge, CB10 1XL, UK

[‡]These authors contributed equally to this work.

*Corresponding author: Matthew I. Hutchings, email: matt.hutchings@jic.ac.uk

*Corresponding author: Barrie Wilkinson, email: barrie.wilkinsons@jic.ac.uk

Table of Contents

1. Table of fasamycin and formicamycin titres 8

2. Analysis of aglycones of glycosylated fasamycins.. 9

3. LCMS and LCMS/MS analysis of glycosylated fasamycins 1-6 10

4. Direct injection HRESIMS analysis of samples 7 and 8 20

5. Carbohydrate analysis of products of acid hydrolysis of glycosylated fasamycins by HPAEC-PAD 22

6. 1D and 2D NMR Spectra and ^{1}H and ^{13}C NMR spectra assignment tables for compounds 1-8. .. 30

7. Bioactivity of glycosylated fasamycin congeners.. 84

Table of Supporting Tables

Table S1. Titres of total fasamycin and formicamycin congeners produced on solid SFM medium in this study (experiments comprised biological triplicates). N.B *S. coelicolor* M1146_215GΔ*forJ* (*n*=2) . 8

Table S2 - Comparison of ^{1}H NMR data for the aglycone isolated from products of acid hydrolysis of sample 5 with the ^{1}H NMR data for fasamycin J (Reference. 1) 28

Table S3 Resonances assignment in ^{1}H and ^{13}C NMR spectra of compound 1a.. 37

Table S4 Resonances assignment in ^{1}H and ^{13}C NMR spectra of compound 2.. 44

Table S5 Resonances assignment in ^{1}H and ^{13}C NMR spectra of compound 3.. 50

Table S6 Resonances assignment in ^{1}H and ^{13}C NMR spectra of compound 4.. 57

Table S7 Resonances assignment in ^{1}H and ^{13}C NMR spectra of compound 5a.. 63

Table S8 Resonances assignment in ^{1}H and ^{13}C NMR spectra of compound 6.. 69

Table S9 Resonances assignment in ^{1}H and ^{13}C NMR spectra of compound 7.. 76

Table S10 Resonances assignment in ^{1}H and ^{13}C NMR spectra of compound 8.. 83

Table of Supporting Figures

Figure S1 Overlay of UV-Vis spectra of fasamycin C and sample 1 (contains compound **1a** and minor compounds **1b** and **1c**). Samples containing fasamycin derivatives were first identified by the presence of characteristic UV-Vis spectra of fasamycin chromophore. 9

Figure S2 LCMS analysis of sample 1 (Thermo Q-Exactive): A, total ion chromatogram; B, extracted ion chromatogram corresponding to **1a** for *m/z* 635.21; C, extracted ion chromatogram corresponding to compounds **1b** and **1c** for *m/z* 767.25. 10

Figure S3 Compound **1a** of sample 1; (**A**) HRESIMS (*m/z* 635.2125 $[M + H]^+$; calc. for $C_{34}H_{35}O_{12}^+$ 635.2123; Δ = 0.3 ppm) and (**B**) MS/MS spectra of ion 635.21. 11

Figure S4 Compounds **1b** and **1c** of sample 1; (**A**) HRESIMS (*m/z* 767.2545 $[M + H]^+$; calc. for $C_{39}H_{43}O_{16}^+$ 767.2546; Δ = -0.1 ppm) and (**B**) MS/MS spectra of ion 767.25. 12

Figure S5 Compound **2** of sample 2; (**A**) HRESIMS (*m/z* 635.2111 $[M + H]^+$; calc. for $C_{34}H_{35}O_{12}^+$ 635.2123; Δ = -1.9 ppm) and (**B**) MS/MS spectra of ion 635.21. 13

Figure S6 Compound **3** of sample 3; (**A**) HRESIMS (*m/z* of 767.2531 $[M + H]^+$; calc for $C_{39}H_{43}O_{16}^+$ 767.2546; Δ = -2.0 ppm) and (**B**) MS/MS spectra of ion 767.25. 14

Figure S7 Compound **4** of sample 4; (**A**) HRESIMS (*m/z* 767.2541 $[M + H]^+$; calc for $C_{39}H_{43}O_{16}^+$ 767.2546; Δ = -0.7 ppm) and (**B**) MS/MS spectra of ion 767.25. 15

Figure S8 LCMS analysis of sample 5 (Thermo Q-Exactive): A, total ion chromatogram; B, extracted ion chromatogram for *m/z* 801.22 corresponding to compound **5a**; C, extracted ion chromatogram for *m/z* 669.17 corresponding to compound **5b**. 16

Figure S9 Compound **5a** of sample 5; (**A**) HRESIMS (*m/z* 801.2151 $[M + H]^+$; calc for $C_{39}H_{42}ClO_{16}^+$ 801.2126; Δ = 3.1 ppm) and (**B**) MS/MS spectra of ion 801.21. 17

Figure S10 Compound **5b** of sample 5; (**A**) HRESIMS (*m/z* 669.1731 $[M + H]^+$; calc for $C_{34}H_{34}ClO_{12}^+$ 669.1733; Δ = -0.3 ppm) and (**B**) MS/MS spectra of ion 669.17. 18

Figure S11 Compound **6** of sample 6; (**A**) HRESIMS (*m/z* 801.2145 $[M + H]^+$; calc for $C_{39}H_{42}ClO_{16}^+$ 801.2156; Δ = -1.4 ppm) and (**B**) MS/MS spectra of ion 801.22. 19

Figure S12 HRESIMS spectrum of sample 7 (*m/z* 649.1904 [M + H]$^+$ (calc for $C_{34}H_{33}O_{13}^+$ 649.1916, Δ = -1.8 ppm)........20
Figure S13 HRESIMS spectrum of sample 8 (*m/z* 649.1920, [M + H]$^+$; calc for $C_{34}H_{33}O_{13}^+$ 649.1916; Δ = 0.6 ppm).........21
Figure S14 The sugars hydrolysed from sample 1 were glucose, galactose and arabinose........22
Figure S15 The sugar hydrolysed from sample 2 was glucose........23
Figure S16 The sugars hydrolysed from sample 3 were glucose and arabinose........23
Figure S17 The carbohydrates hydrolysed from sample 4 were glucose and arabinose, with a minor peak of galactose........24
Figure S18 -The sugars hydrolysed from sample 5 were glucose, galactose and arabinose........24
Figure S19 The sugars hydrolysed from sample 6 were glucose and galactose........25
Figure S20 The sugar hydrolysed from sample 7 was glucuronic acid........25
Figure S21 The sugar hydrolysed from sample 8 was glucuronic acid........26
Figure S22 HPLC confirmed the aglycone after acid hydrolysis of samples 1, 2, 3 and 4 were fasamycin C by comparison with an authentic fasamycin C sample........27
Figure S23 Overlay of HPLC traces of fasamycin C (blue) with the hydrolysed fasamycin aglycones of sample 5 (green) and 6 (red). LCMS showed that the aglycones of samples 5 and 6 have the same mass but different retention times........27
Figure S24 ^{1}H NMR (600 MHz, 298 K, CD_3OD) of fasamycin J, the aglycone released by acid hydrolysis of sample 5........29
Figure S25 ^{1}H NMR spectrum (CD_3OD, 600 MHz, 298K) of fasamycin glycosides of Sample 1. Assignment of resonances of compound **1a** is shown on the main spectrum. Insert: expansion of the region showing provisional assignment of anomeric signals of disaccharide glycosides **1b** and **1c**....30
Figure S26 ^{13}C NMR spectrum (CD_3OD, 150 MHz, 298K) of fasamycin glycosides of sample 1. Assignment of resonances of compound **1a** is shown........31

Figure S27 ^1H-^1H COSY spectrum (CD_3OD, 298K) of fasamycin glycosides of sample 1. Assignment of resonances of compound **1a** is shown. 32

Figure S28 ^1H-^{13}C HSQC-edited spectrum (CD_3OD, 298K) of fasamycin glycosides of sample 1. Assignment of resonances of compound **1a** is shown........ 33

Figure S29 Anomeric region of coupled ^1H-^{13}C HSQC spectrum (CD_3OD, 298K) of fasamycin glycosides of sample 1. The size of $^1J_{C1Glc,H1Glc}$ coupling constants is measured along F2 dimension. .. 34

Figure S30 ^1H-^{13}C HMBC spectrum (CD_3OD, 298K) of fasamycin glycosides of sample 1. Assignment of resonances of compound **1a** is shown........ 35

Figure S31 ^1H-^1H ROESY spectrum (CD_3OD, 298K) of fasamycin glycosides of sample 1. Assignment of resonances of compound **1a** is shown........ 36

Figure S32 ^1H NMR spectrum (CD_3OD, 600 MHz, 298K) of fasamycin glycoside **2**........ 38

Figure S33 ^{13}C NMR spectrum (CD_3OD, 150 MHz, 298K) of fasamycin glycoside **2**........ 39

Figure S34 ^1H-^1H COSY spectrum (CD_3OD, 298K) of fasamycin glycoside **2**........ 40

Figure S35 ^1H-^{13}C HSQC-edited spectrum (CD_3OD, 298K) of fasamycin glycoside **2**........ 41

Figure S36 ^1H-^{13}C HMBC spectrum (CD_3OD, 298K) of fasamycin glycoside **2** 42

Figure S37 ^1H-^1H ROESY spectrum (CD_3OD, 600 MHz, 298K) of fasamycin glycoside **2** 43

Figure S38 ^1H NMR spectrum (CD_3OD, 600 MHz, 298K) of fasamycin glycoside **3**........ 45

Figure S39 ^1H-^1H COSY spectrum (CD_3OD, 298K) of fasamycin glycoside **3**........ 46

Figure S40 ^1H-^{13}C HSQC-edited spectrum (CD_3OD, 298K) of fasamycin glycoside **3**........ 47

Figure S41 ^1H-^{13}C HMBC spectrum (CD_3OD, 298K) of fasamycin glycoside **3** 48

Figure S42 ^1H-^1H ROESY spectrum (CD_3OD, 298K) of fasamycin glycoside **3**........ 49

Figure S43 ^1H NMR spectrum (CD_3OD, 600 MHz, 298K) of fasamycin glycoside **4**........ 51

Figure S44 ^{13}C NMR spectrum (CD_3OD, 150 MHz, 298K) of fasamycin glycoside **4**........ 52

Figure S45 ^1H-^1H COSY spectrum (CD3OD, 298K) of fasamycin glycoside **4** 53

Figure S46 ^1H-^{13}C HSQC-edited spectrum (CD_3OD, 298K) of fasamycin glycoside **4**........ 54

Figure S47 ^1H-^{13}C HMBC spectrum (CD_3OD, 298K) of fasamycin glycoside **4** 55

Figure S48 ^1H-^1H ROESY spectrum (CD_3OD, 298K) of fasamycin glycoside **4**................................56

Figure S49 ^1H NMR spectrum (CD_3OD, 600 MHz, 298K) of fasamycin glycoside **5a**.......................58

Figure S50 ^1H-^1H COSY spectrum (CD_3OD, 298K) of fasamycin glycoside **5a**................................59

Figure S51 ^1H-^{13}C HSQC-edited spectrum (CD_3OD, 298K) of fasamycin glycoside **5a**....................60

Figure S52 ^1H-^{13}C HMBC spectrum (CD_3OD, 298K) of fasamycin glycoside **5a** 61

Figure S53 ^1H-^1H ROESY spectrum (CD_3OD, 298K) of fasamycin glycoside **5a**...............................62

Figure S54 ^1H NMR spectrum (CD_3OD, 600 MHz, 298K) of fasamycin glycoside **6**........................64

Figure S55 ^1H-^1H COSY spectrum (CD_3OD, 298K) of fasamycin glycoside **6** zoomed in carbohydrate signals region 65

Figure S56 ^1H-^{13}C HSQC-edited spectrum (CD_3OD, 298K) of fasamycin glycoside **6**......................66

Figure S57 ^1H-^{13}C HMBC spectrum (CD_3OD, 298K) of fasamycin glycoside **6** 67

Figure S58 ^1H-^1H ROESY spectrum (CD_3OD, 298K) of fasamycin glycoside **6**................................68

Figure S59 ^1H NMR spectrum (CD_3OD, 600 MHz, 298K) of fasamycin glycoside **7**........................70

Figure S60 ^{13}C NMR spectrum (CD_3OD, 150 MHz, 298K) of fasamycin glycoside **7**.......................71

Figure S61 ^1H-^1H COSY spectrum (CD_3OD, 298K) of fasamycin glycoside **7**..................................72

Figure S62 1H-13C HSQC-edited spectrum (CD_3OD, 298K) of fasamycin glycoside **7** 73

Figure S63 ^1H-^{13}C HMBC spectrum (CD_3OD, 298K) of fasamycin glycoside **7** 74

Figure S64 ^1H-^1H NOESY spectrum (CD_3OD, 298K) of fasamycin glycoside **7** 75

Figure S65 ^1H NMR spectrum (CD_3OD, 600 MHz, 298K) of fasamycin glycoside **8** 77

Figure S66 ^{13}C NMR spectrum (CD_3OD, 150 MHz, 298K) of fasamycin glycoside **8**.......................78

Figure S67 ^1H-^1H COSY spectrum (CD_3OD, 298K) of fasamycin glycoside **8**..................................79

Figure S68 ^1H-^{13}C HSQC-edited spectrum (CD_3OD, 298K) of fasamycin glycoside **8**......................80

Figure S69 ^1H-^{13}C HMBC spectrum (CD_3OD, 298K) of fasamycin glycoside **8** 81

Figure S70 ^1H-^1H ROESY spectrum (CD_3OD, 298K) of fasamycin glycoside **8**................................82

Figure S71 Representative examples of spot on lawn bioassay plates to determine bioactivity of samples 1-6. E. coli ATCC 25922 and S. aureus ATCC BA-1717 were grown to confluence in soft

nutrient agar and B. subtilis 168 was grown in LB agar medium containing no salt. Samples 1 (420 µg/mL), 2 (440 µg/mL), 3 (120 µg/mL), 4 (400 µg/mL), 5 (250 µg/mL), 6 (300 µg/mL) and apramycin (150 µg/mL) in methanol were spotted directly onto the agar and the plates were grown overnight at 37 ˚C. The positive control, apramycin (Apra), showed a zone of inhibition whereas none of samples 1-6 showed any activity against the strains tested..84

1. Table of fasamycin and formicamycin titres

Table S1. Titres of total fasamycin and formicamycin congeners produced on solid SFM medium in this study (experiments comprised biological triplicates). N.B *S. coelicolor* M1146_215GΔ*forJ* (*n*=2)

**Strain**	**Titre (µM)**		
	**Fasamycins**	**Formicamycins**	**Combined fasamycins and formicamycins**
WT *S. formicae*	20.6 ± 6.1	75.5 ± 3.5	96.1 ± 9.6
S. formicae Δ*forJ*	144.3 ± 21.0	455.0 ± 38.1	599.3 ± 59.1
S. coelicolor M1146	0	0	0
S. coelicolor M1146_215G	0	0	0
S. coelicolor M1146_215GΔ*forJ*	0.4 ± 0.3	31.2 ± 5.1	31.6 ± 5.4
S. erythraea Δ*ery*	0	0	0
S. erythraea Δ*ery*_215G	0	0	0
S. erythraea Δ*ery*_215GΔ*forJ*	33.4 ± 21.1	2.1± 1.3	35.5 ± 24.4

2. Analysis of aglycones of glycosylated fasamycins

UV-Visible spectroscopy of glycosylated fasamycins

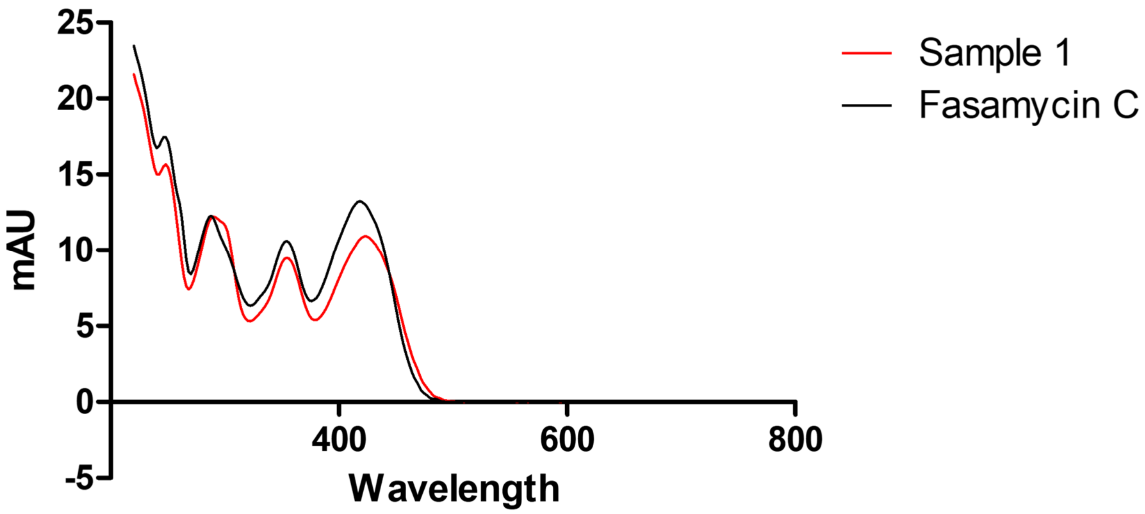

Figure S1 Overlay of UV-Vis spectra of fasamycin C and sample 1 (contains compound **1a** and minor compounds **1b** and **1c**). Samples containing fasamycin derivatives were first identified by the presence of characteristic UV-Vis spectra of fasamycin chromophore.

3. LCMS and LCMS/MS analysis of glycosylated fasamycins 1-6

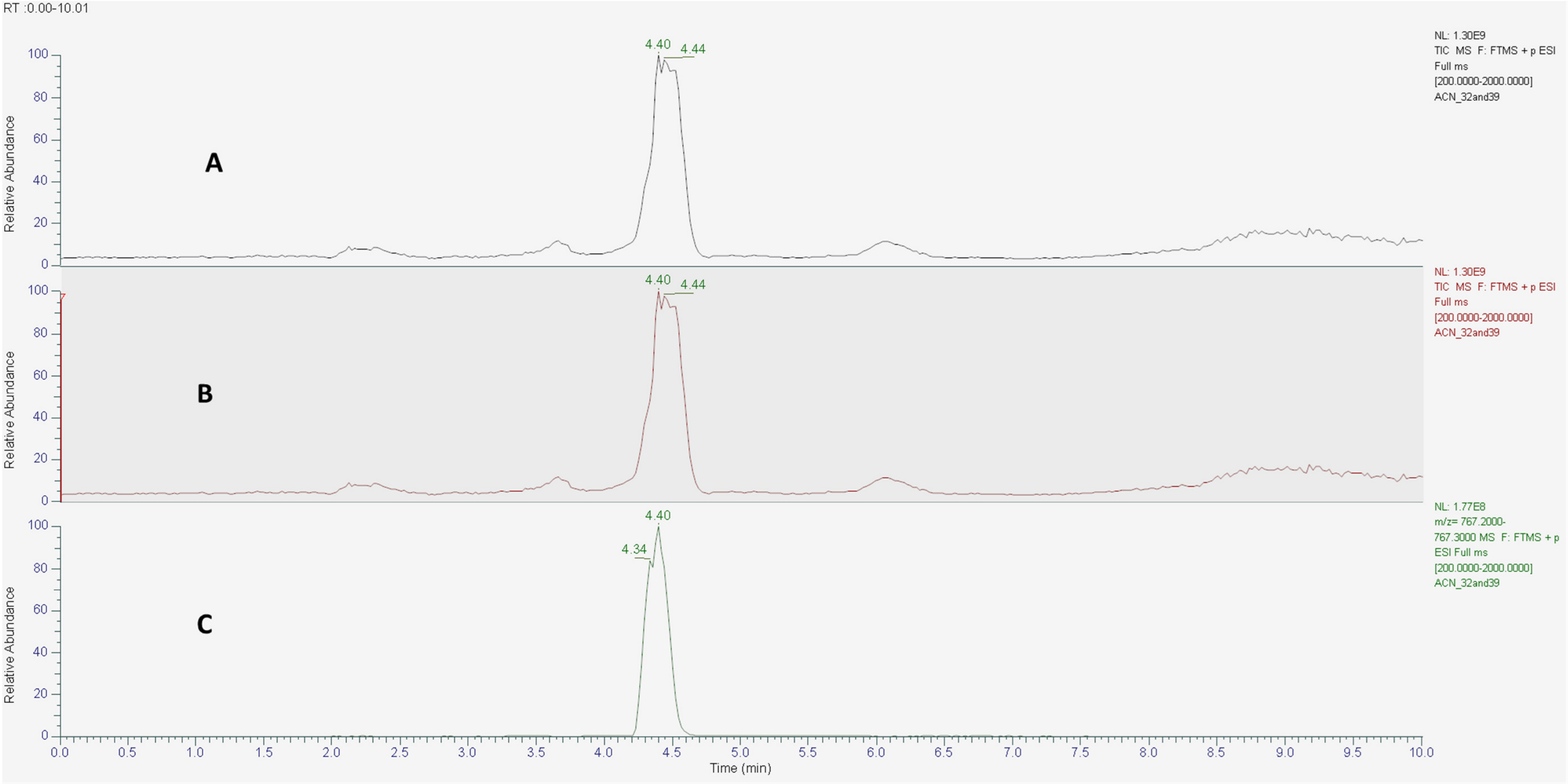

Figure S2 LCMS analysis of sample 1 (Thermo Q-Exactive): A, total ion chromatogram; B, extracted ion chromatogram corresponding to **1a** for *m/z* 635.21; C, extracted ion chromatogram corresponding to compounds **1b** and **1c** for *m/z* 767.25.

A

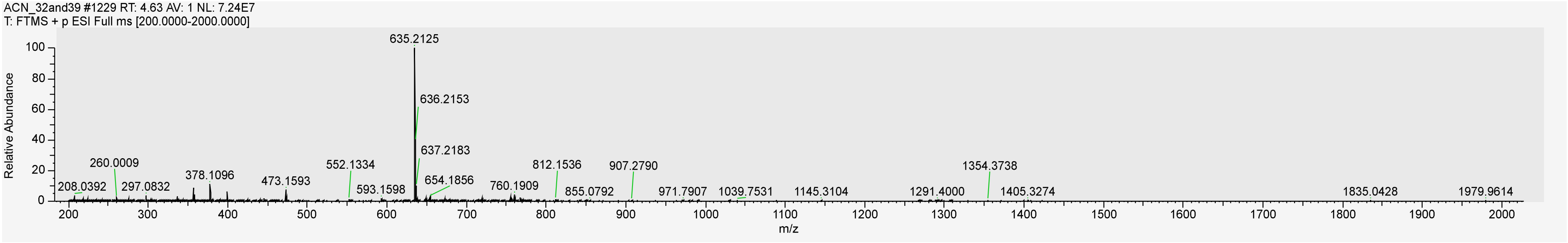

B

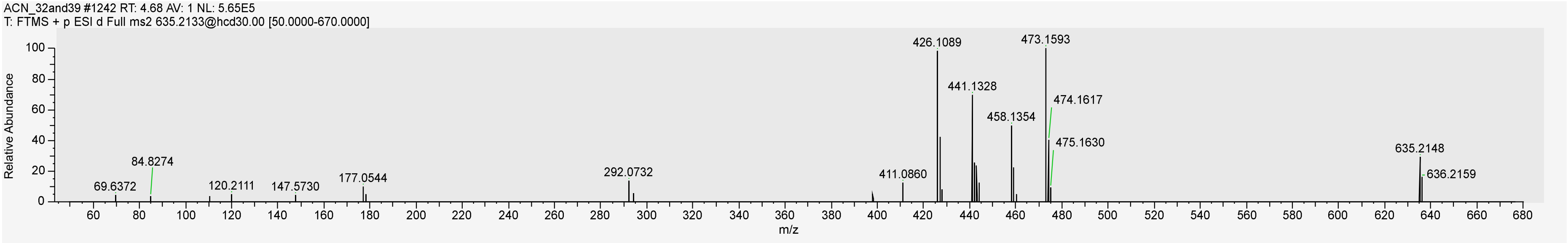

Figure S3 Compound **1a** of sample 1; (**A**) HRESIMS (*m/z* 635.2125 [M + H]$^+$; calc. for $C_{34}H_{35}O_{12}^+$ 635.2123; Δ = 0.3 ppm) and (**B**) MS/MS spectra of ion 635.21.

A

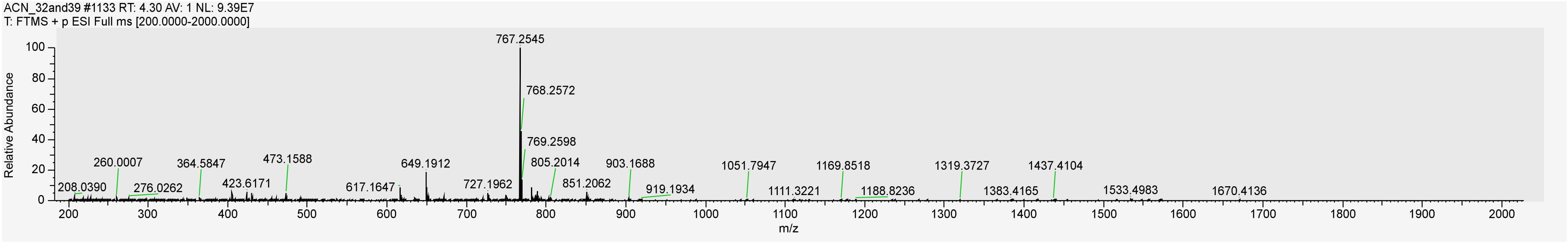

B

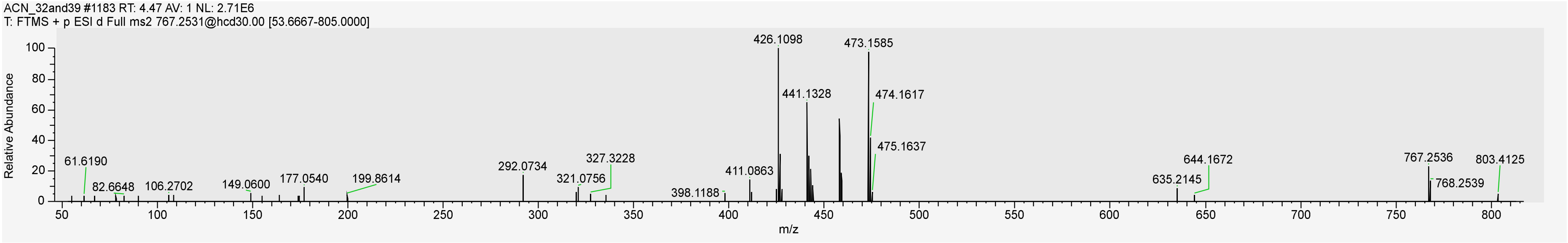

Figure S4 Compounds **1b** and **1c** of sample 1; (**A**) HRESIMS (*m/z* 767.2545 [M + H]$^+$; calc. for $C_{39}H_{43}O_{16}^+$ 767.2546; Δ = -0.1 ppm) and (**B**) MS/MS spectra of ion 767.25.

A

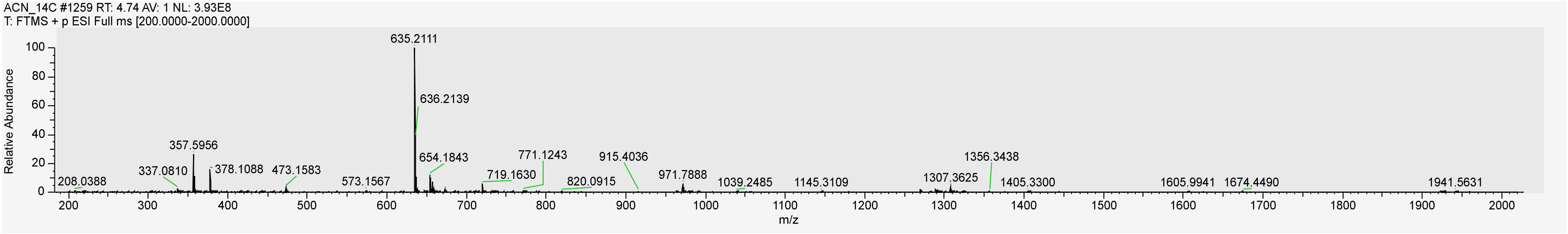

B

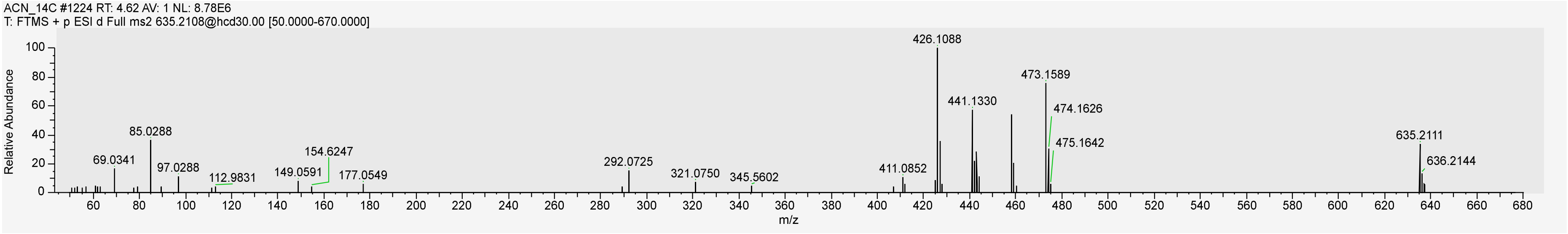

Figure S5 Compound **2** of sample 2; (**A**) HRESIMS (*m/z* 635.2111 [M + H]$^+$; calc. for $C_{34}H_{35}O_{12}^+$ 635.2123; Δ = -1.9 ppm) and (**B**) MS/MS spectra of ion 635.21.

A

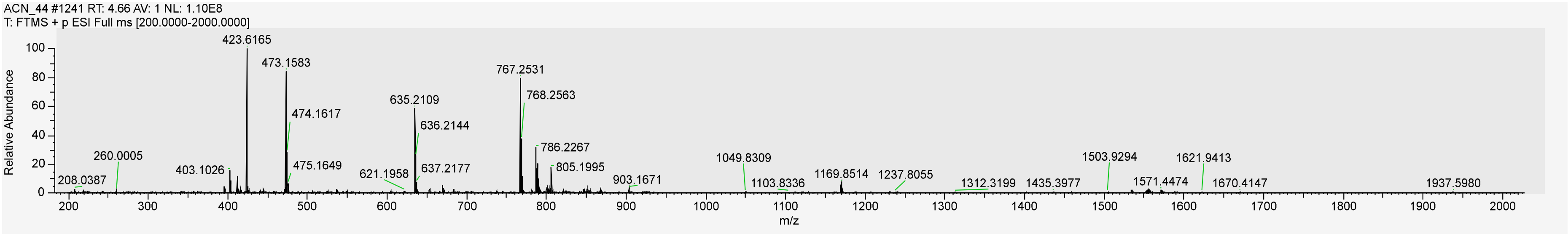

B

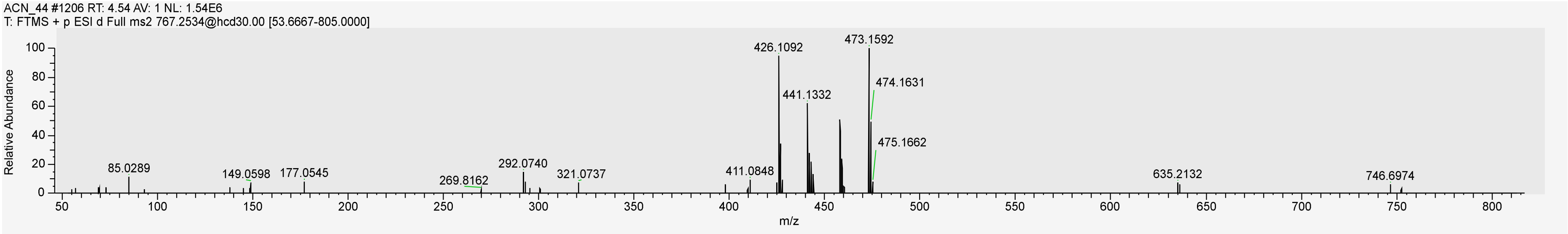

Figure S6 Compound **3** of sample 3; (**A**) HRESIMS (*m/z* of 767.2531 [M + H]$^+$; calc for $C_{39}H_{43}O_{16}^+$ 767.2546; Δ = -2.0 ppm) and (**B**) MS/MS spectra of ion 767.25.

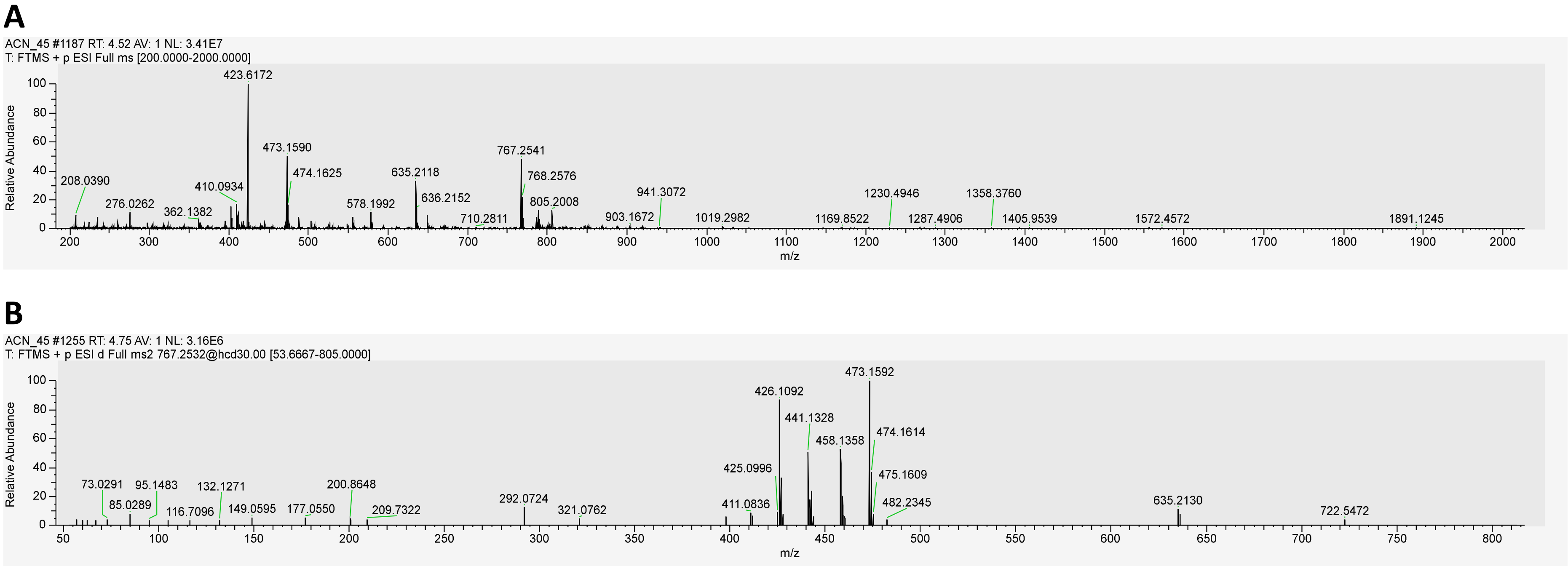

Figure S7 Compound **4** of sample 4; (**A**) HRESIMS (*m/z* 767.2541 [M + H]$^+$; calc for $C_{39}H_{43}O_{16}^+$ 767.2546; Δ = -0.7 ppm) and (**B**) MS/MS spectra of ion 767.25.

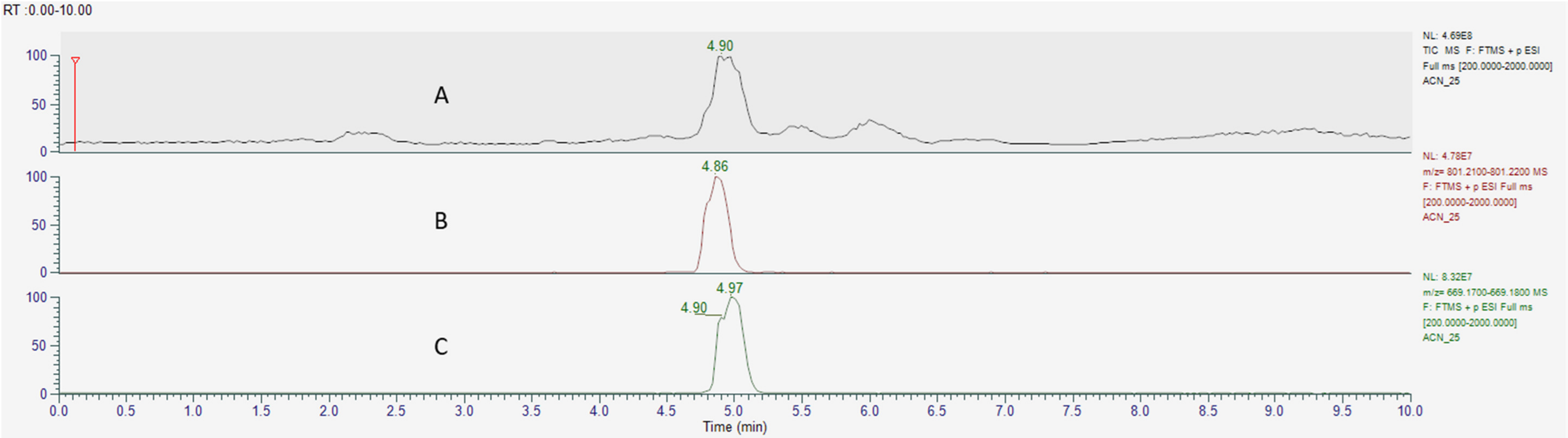

Figure S8 LCMS analysis of sample 5 (Thermo Q-Exactive): A, total ion chromatogram; B, extracted ion chromatogram for *m/z* 801.22 corresponding to compound **5a**; C, extracted ion chromatogram for *m/z* 669.17 corresponding to compound **5b**.

A

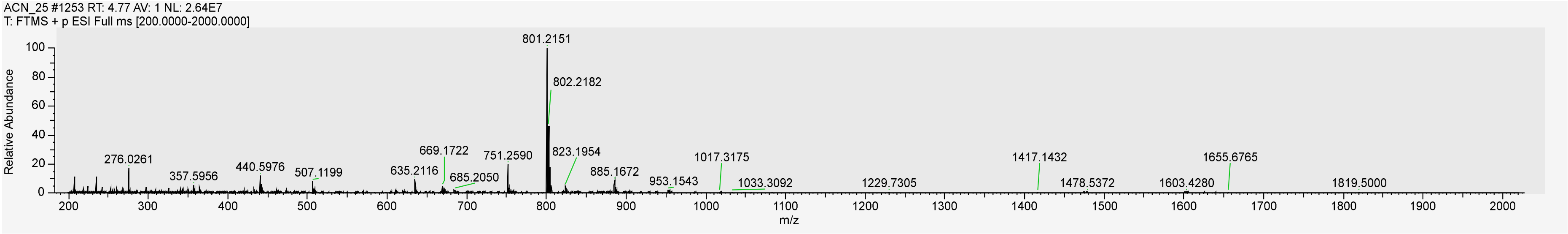

B

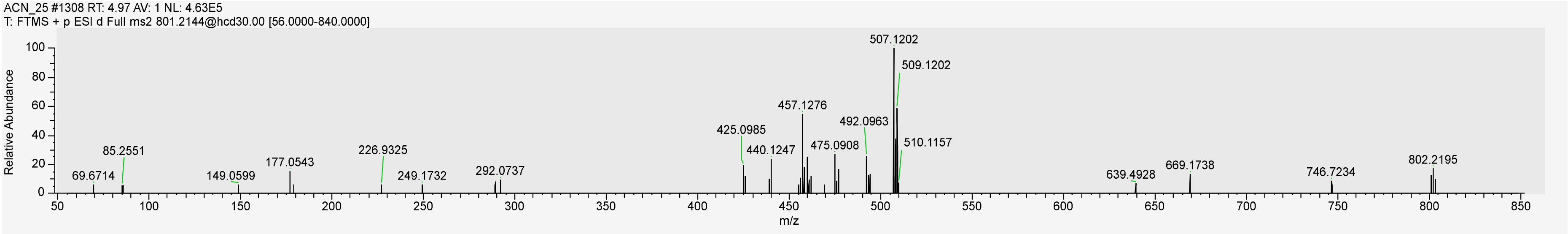

Figure S9 Compound **5a** of sample 5; (**A**) HRESIMS (*m/z* 801.2151 [M + H]$^+$; calc for $C_{39}H_{42}ClO_{16}^+$ 801.2156; Δ = -0.6 ppm) and (**B**) MS/MS spectra of ion 801.21.

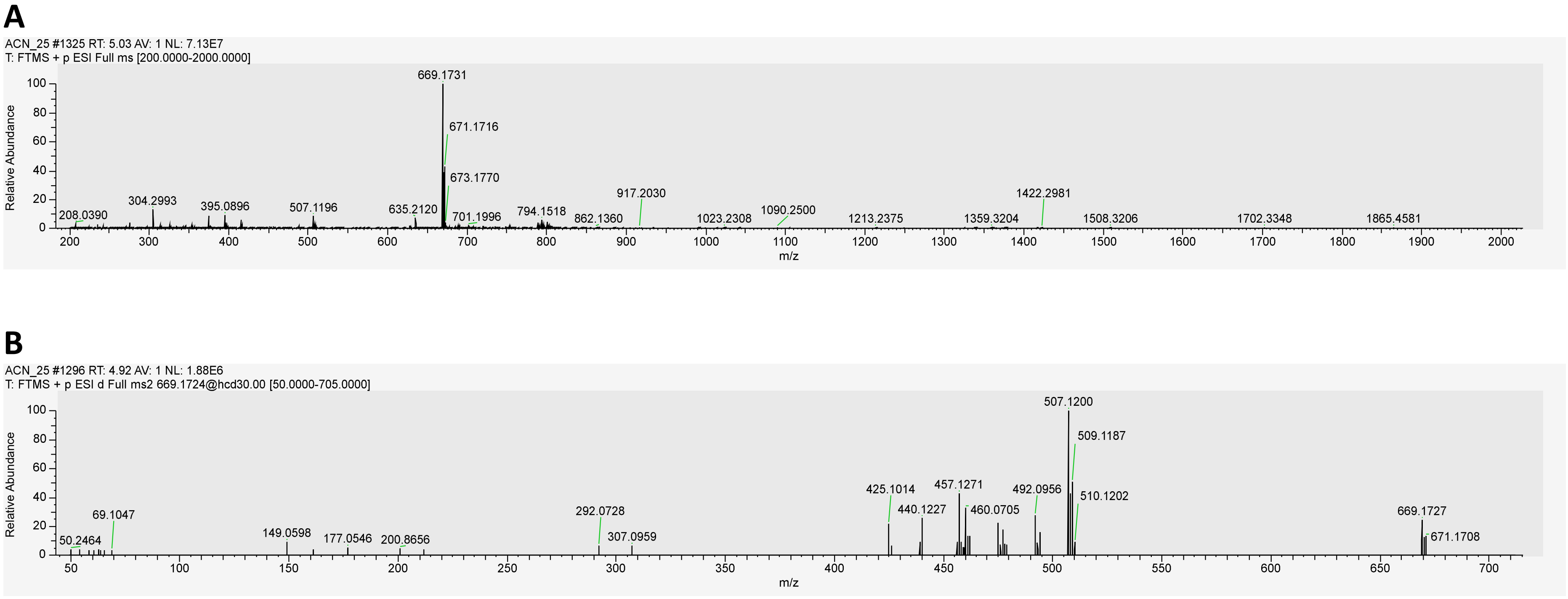

Figure S10 Compound **5b** of sample 5; (**A**) HRESIMS (*m/z* 669.1731 [M + H]$^+$; calc for $C_{34}H_{34}ClO_{12}^+$ 669.1733; Δ = -0.3 ppm) and (**B**) MS/MS spectra of ion 669.17.

A

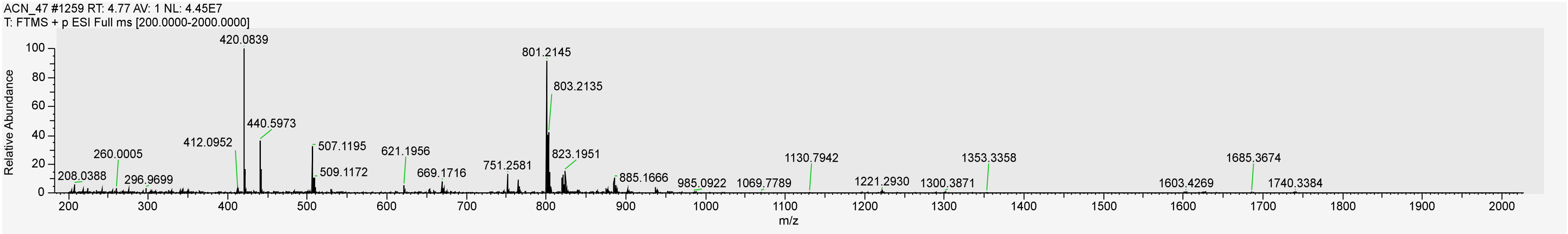

B

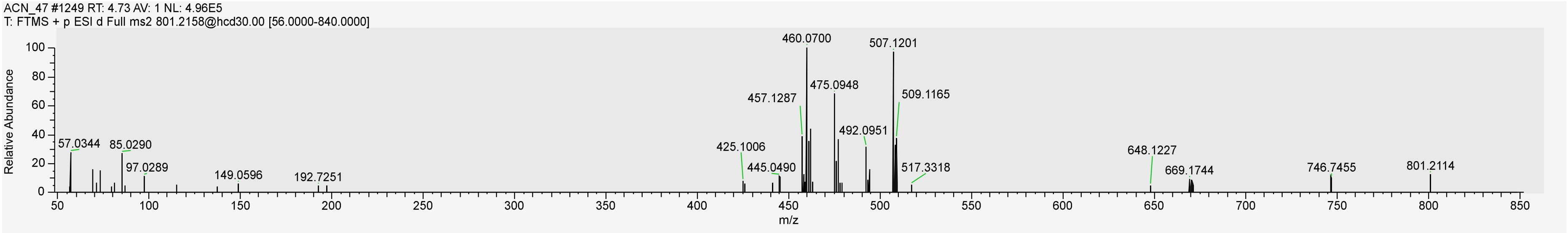

Figure S11 Compound **6** of sample 6; (**A**) HRESIMS (*m/z* 801.2145 [M + H]$^+$; calc for $C_{39}H_{42}ClO_{16}^+$ 801.2156; Δ = -1.4 ppm) and (**B**) MS/MS spectra of ion 801.22.

4. Direct injection HRESIMS analysis of samples 7 and 8

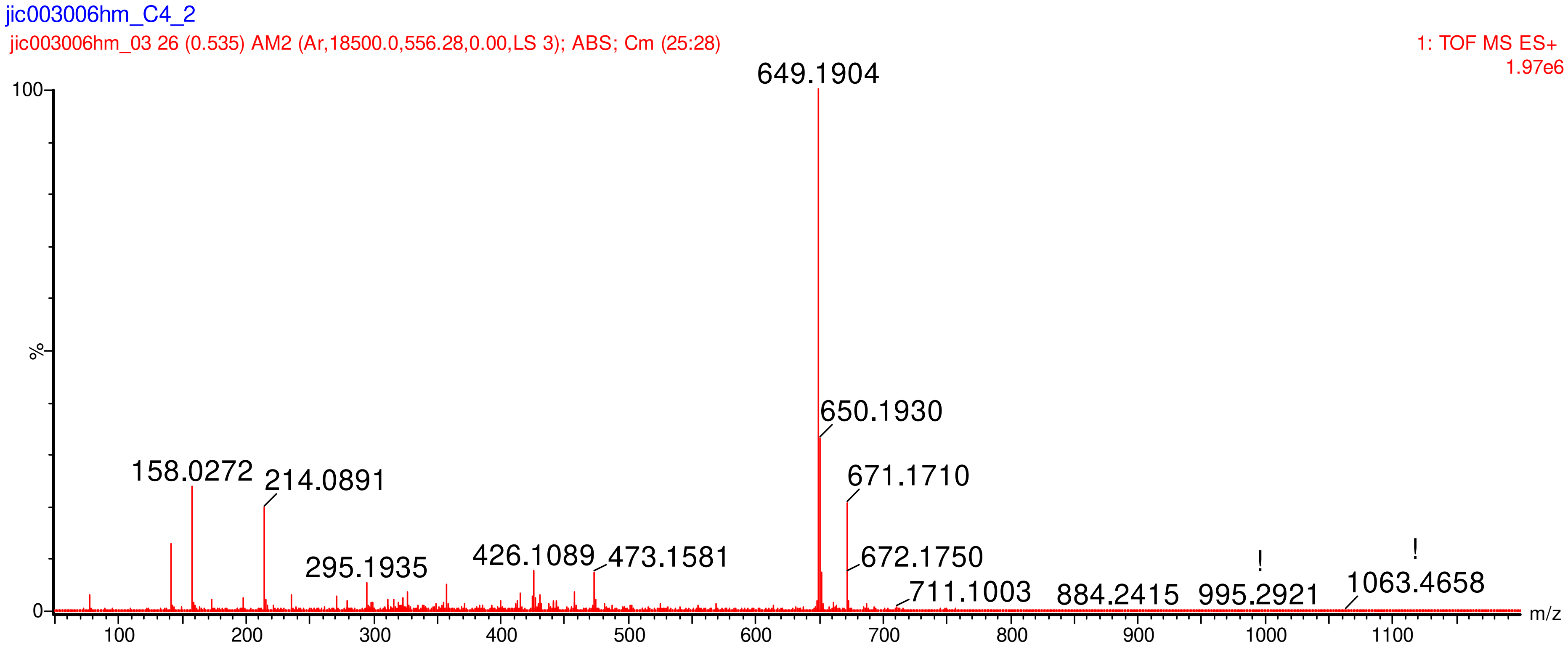

Figure S12 HRESIMS spectrum of sample 7 (*m/z* 649.1904 $[M + H]^+$ (calc for $C_{34}H_{33}O_{13}^+$ 649.1916, Δ = -1.8 ppm).

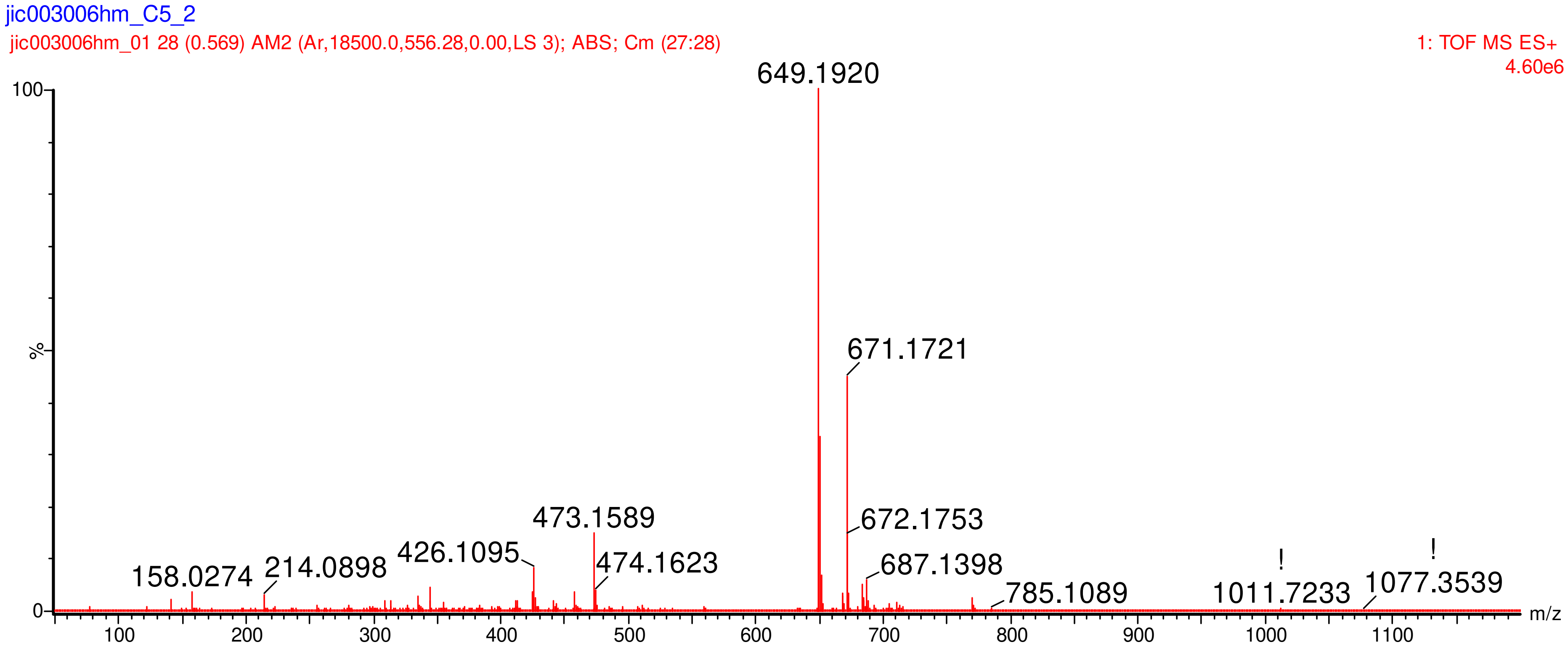

Figure S13 HRESIMS spectrum of sample 8 (*m/z* 649.1920, [M + H]$^+$; calc for $C_{34}H_{33}O_{13}^+$ 649.1916; Δ = 0.6 ppm).

5. Carbohydrate analysis of products of acid hydrolysis of glycosylated fasamycins by HPAEC-PAD

The carbohydrates liberated by hydrolysis were analysed by HPAEC-PAD, in repeated injections the samples were spiked with carbohydrate standards for verification. A screen against glucose, galactose, mannose, arabinose, ribose, xylose, glucuronic acid and galacturonic acid standards were performed and results are shown in Figures S14-21.

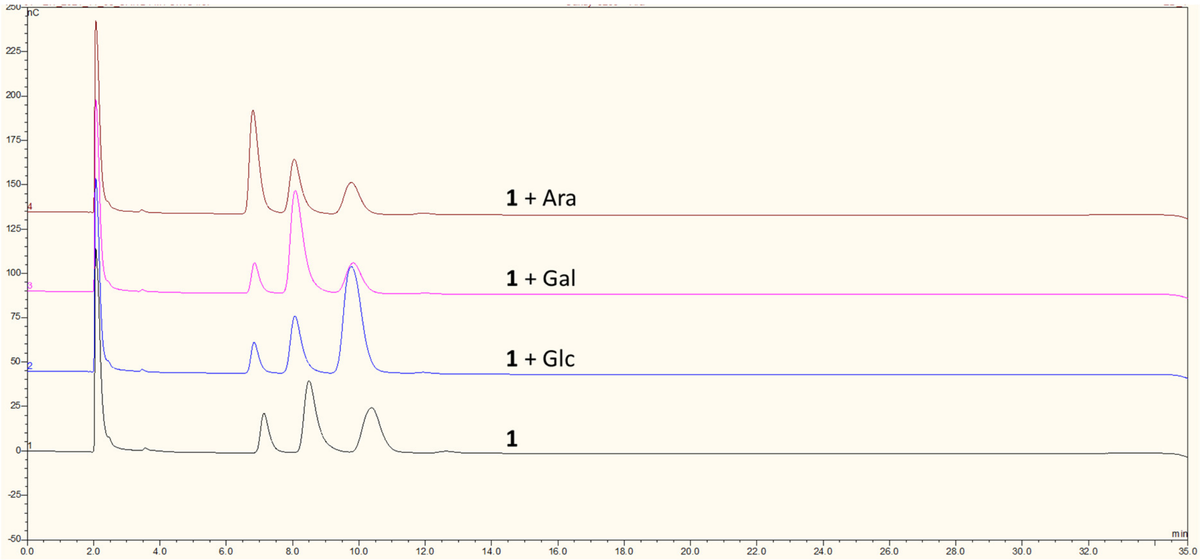

Figure S14 The sugars hydrolysed from sample 1 were glucose, galactose and arabinose

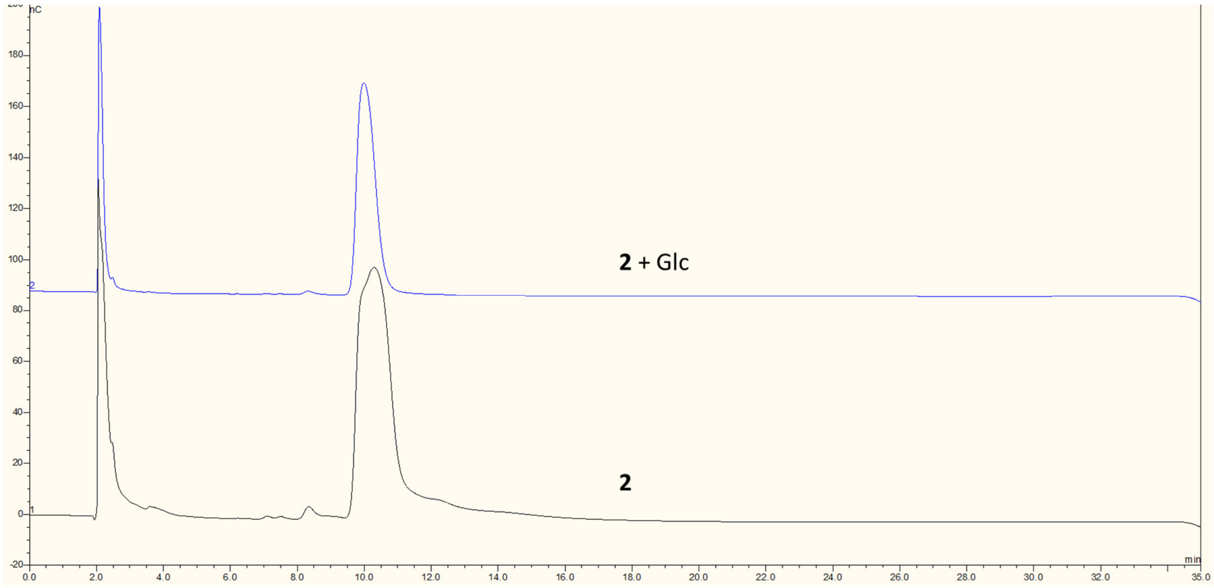

Figure S15 The sugar hydrolysed from sample 2 was glucose

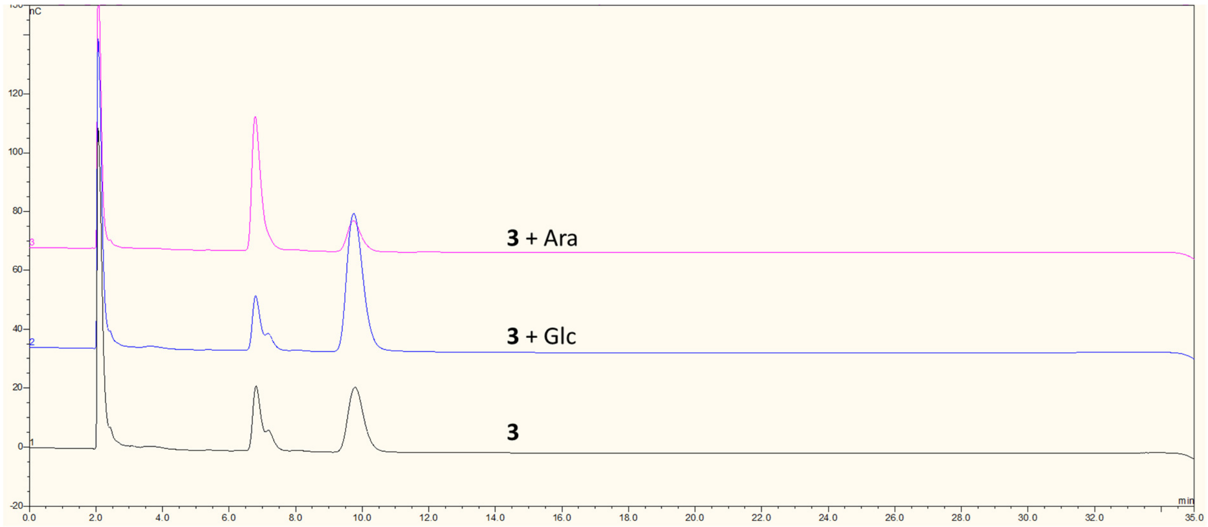

Figure S16 The sugars hydrolysed from sample 3 were glucose and arabinose

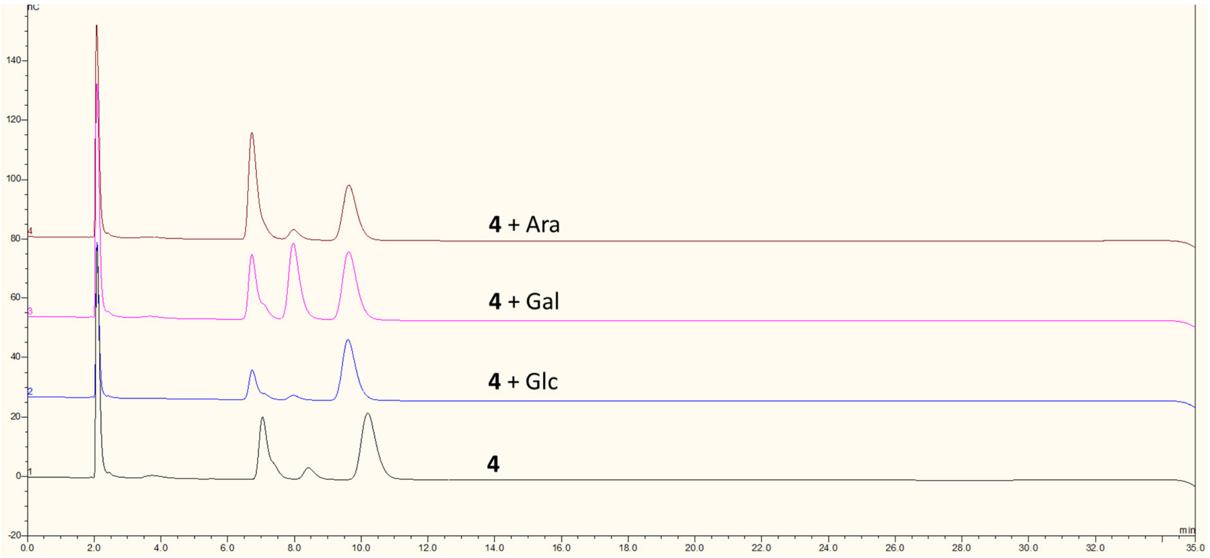

Figure S17 The carbohydrates hydrolysed from sample 4 were glucose and arabinose, with a minor peak of galactose

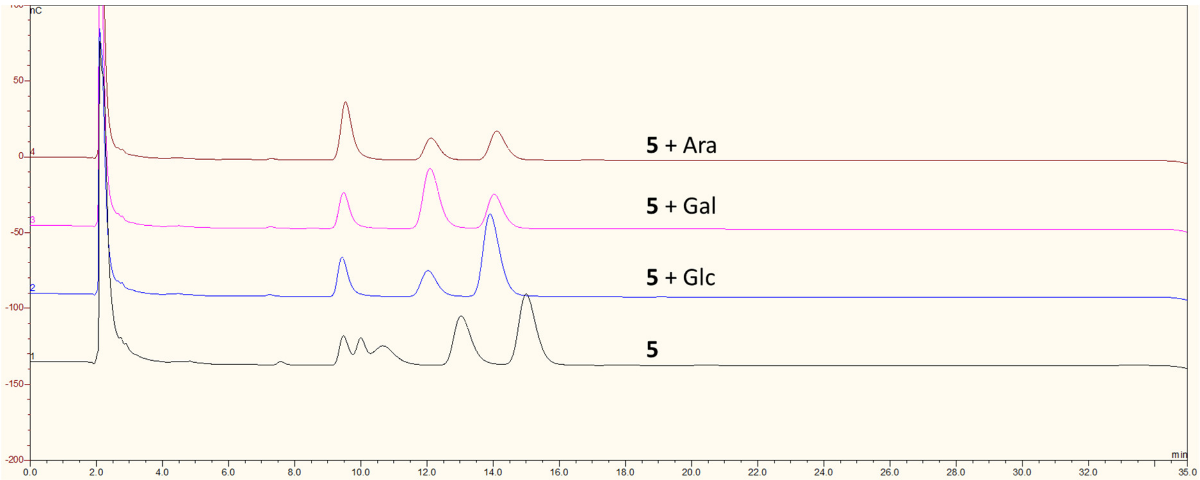

Figure S18 -The sugars hydrolysed from sample 5 were glucose, galactose and arabinose

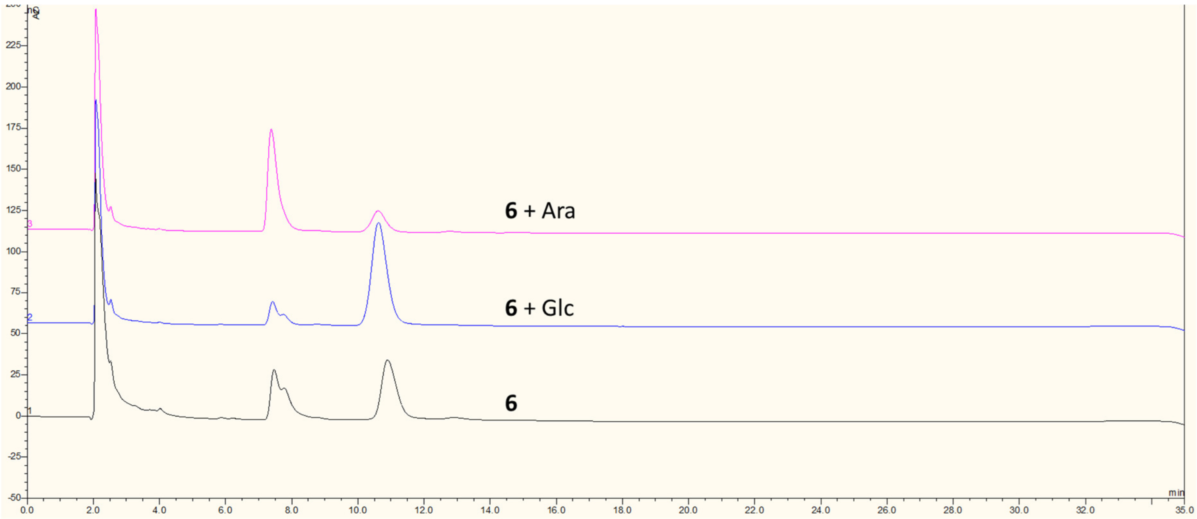

Figure S19 The sugars hydrolysed from sample 6 were glucose and galactose

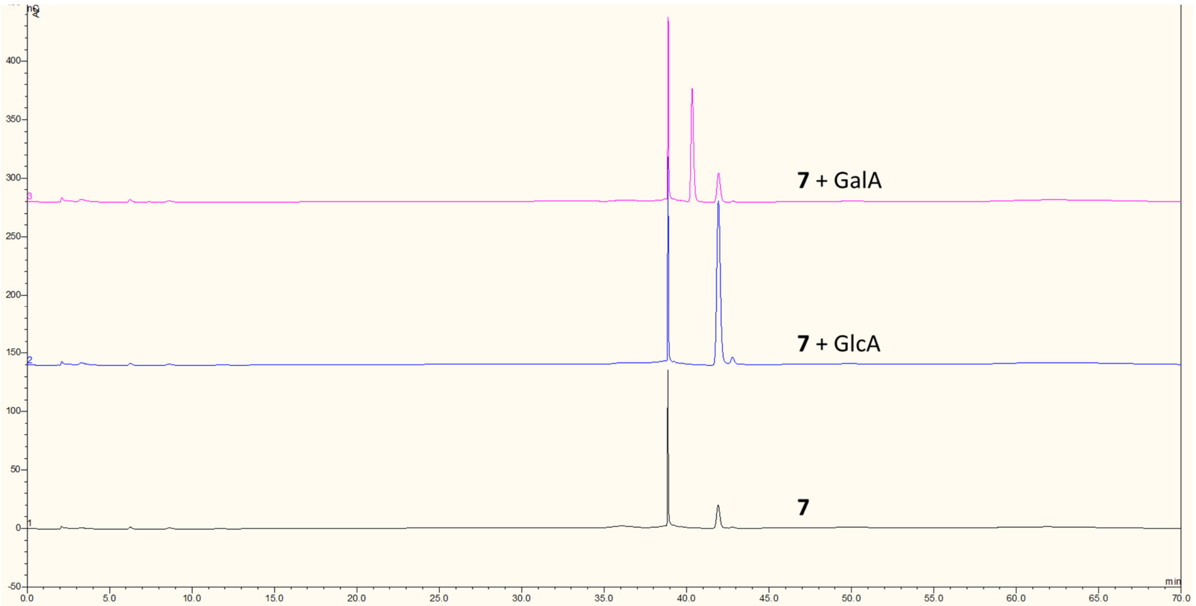

Figure S20 The sugar hydrolysed from sample 7 was glucuronic acid

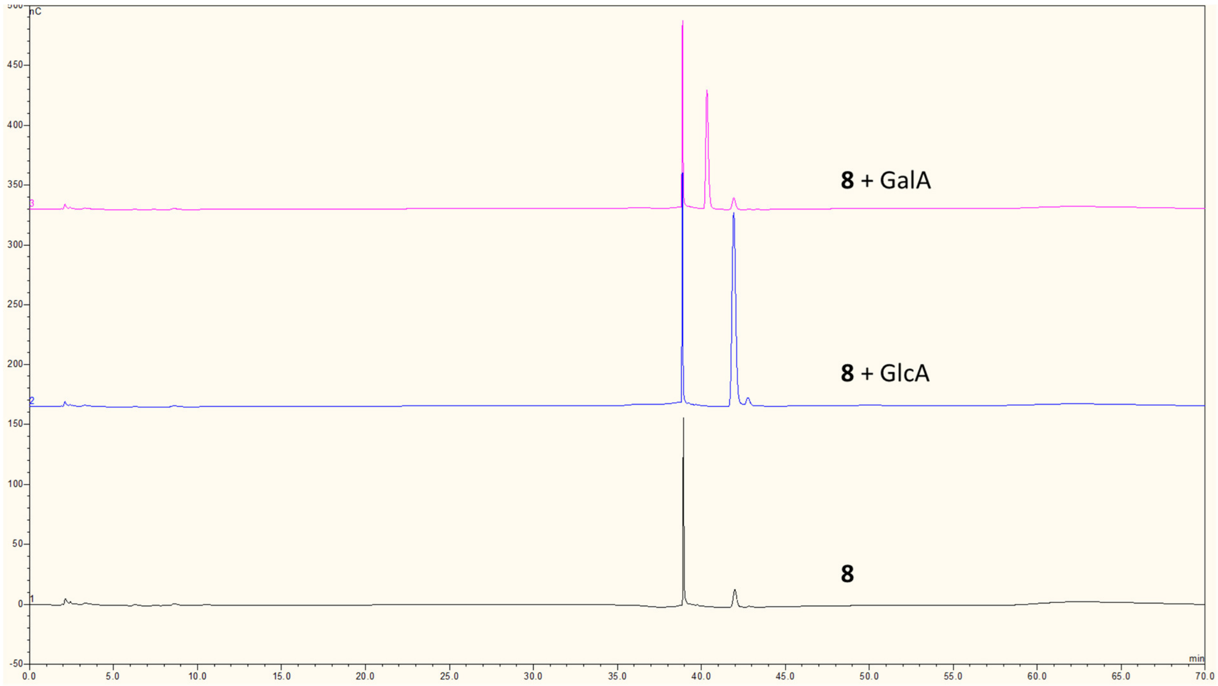

Figure S21 The sugar hydrolysed from sample 8 was glucuronic acid

3.2 HPLC analysis of aglycones after acid hydrolysis

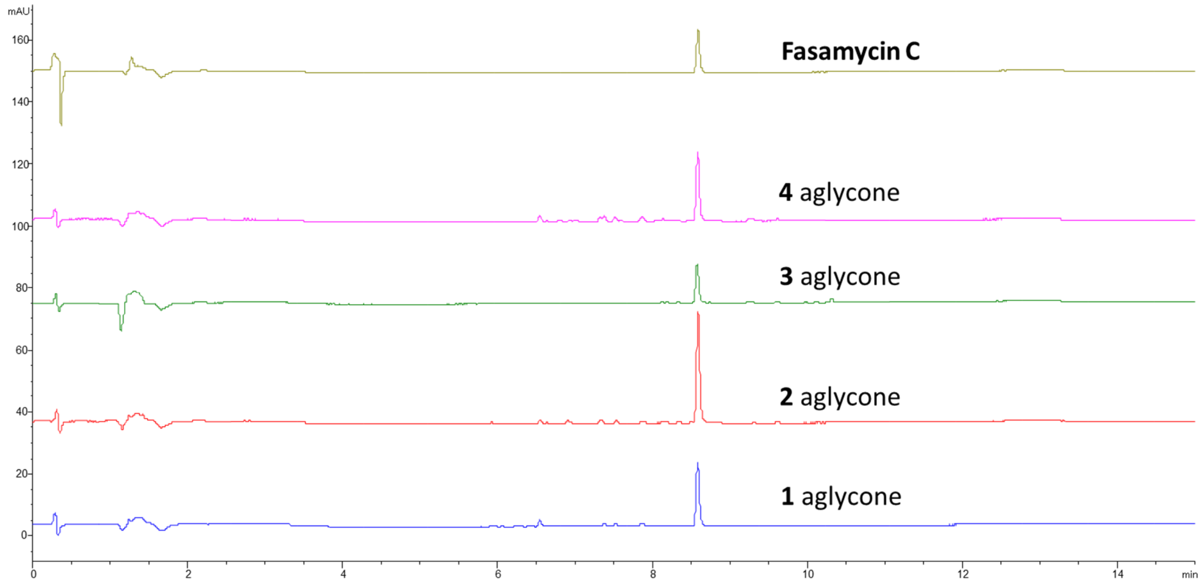

Figure S22 HPLC confirmed the aglycone after acid hydrolysis of samples 1, 2, 3 and 4 were fasamycin C by comparison with an authentic fasamycin C sample.

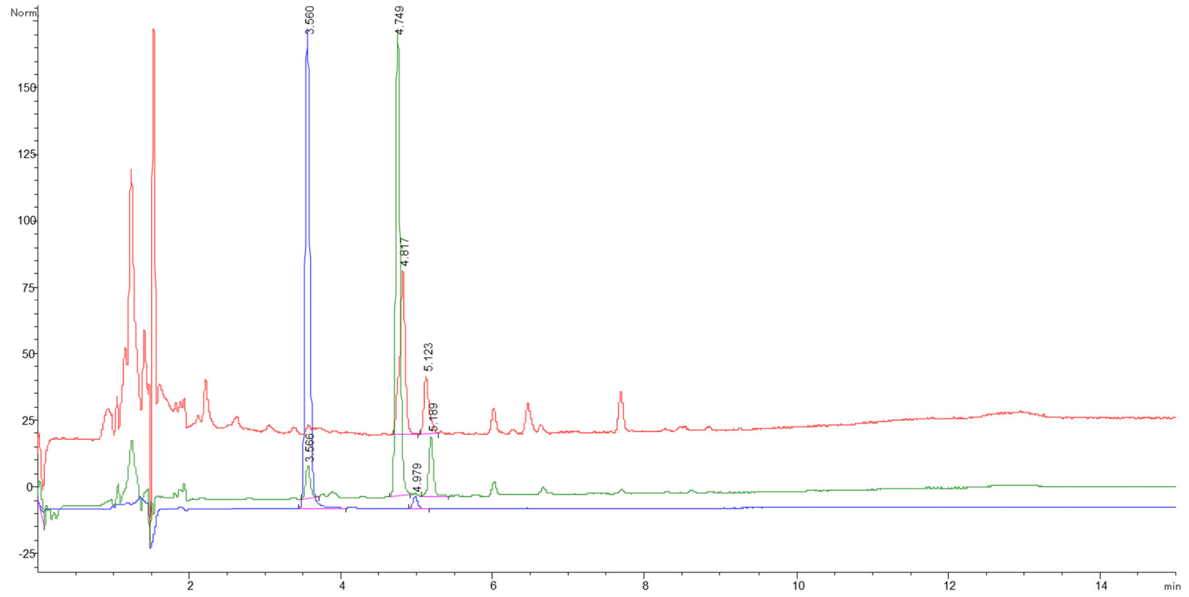

Figure S23 Overlay of HPLC traces of fasamycin C (blue) with the hydrolysed fasamycin aglycones of sample 5 (green) and 6 (red). LCMS showed that the aglycones of samples 5 and 6 have the same mass but different retention times.

3.3 NMR analysis of the aglycone resulted from the acid hydrolysis of sample 5.

Table S2 - Comparison of ^{1}H NMR data for the aglycone isolated from products of acid hydrolysis of sample 5 with the ^{1}H NMR data for fasamycin J (Reference. 1)

**Proton**	**Chemical shift, δ (coupling constant, Hz)**	
	Published (400 MHz, CD_3OD) (Ref. 1)	Observed (600 MHz, CD_3OD)
4	6.51 s	6.52 s
14	6.20 d (3.0)	6.20 d (2.3)
16	6.65 d (3.0)	6.66 d (2.3)
20	7.37 s	7.37 s
22	7.05 d (3.0)	7.06 d (2.5)
24	6.64 d (3.6)	6.64 d (2.6)
25	1.98 s	1.99 s
26	1.71 s	1.71 s
27	1.71 s	1.71 s
28	3.59 s	3.60 s

Reference: Jie Yaun et al., *J. Nat. Prod.* 2020, 83, 6, 1919–1924.
https://pubs.acs.org/doi/abs/10.1021/acs.jnatprod.0c00161

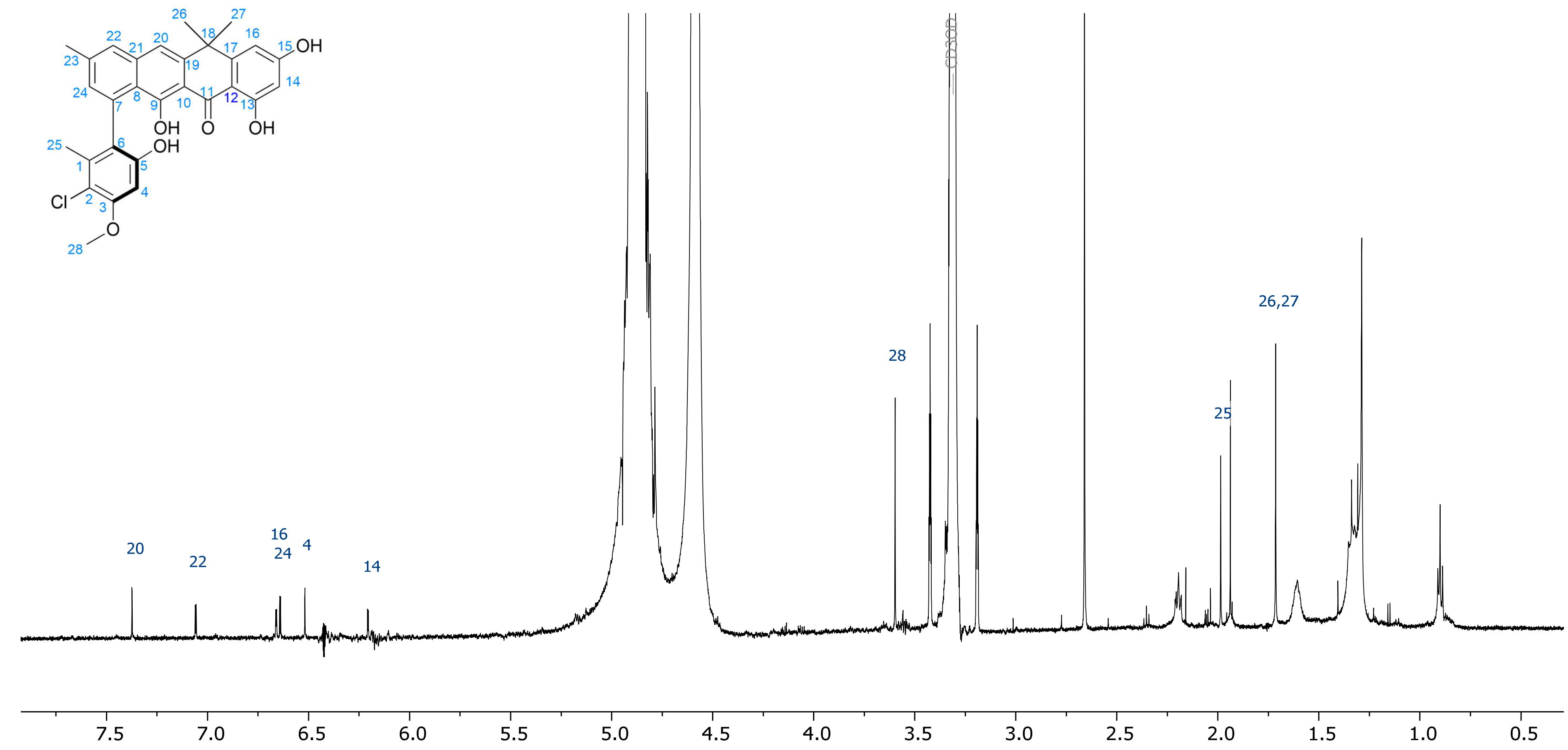

Figure S24 ^1H NMR (600 MHz, 298 K, CD_3OD) of fasamycin J, the aglycone released by acid hydrolysis of sample 5.

6. 1D and 2D NMR Spectra and ^{1}H and ^{13}C NMR spectra assignment tables for compounds 1-8.

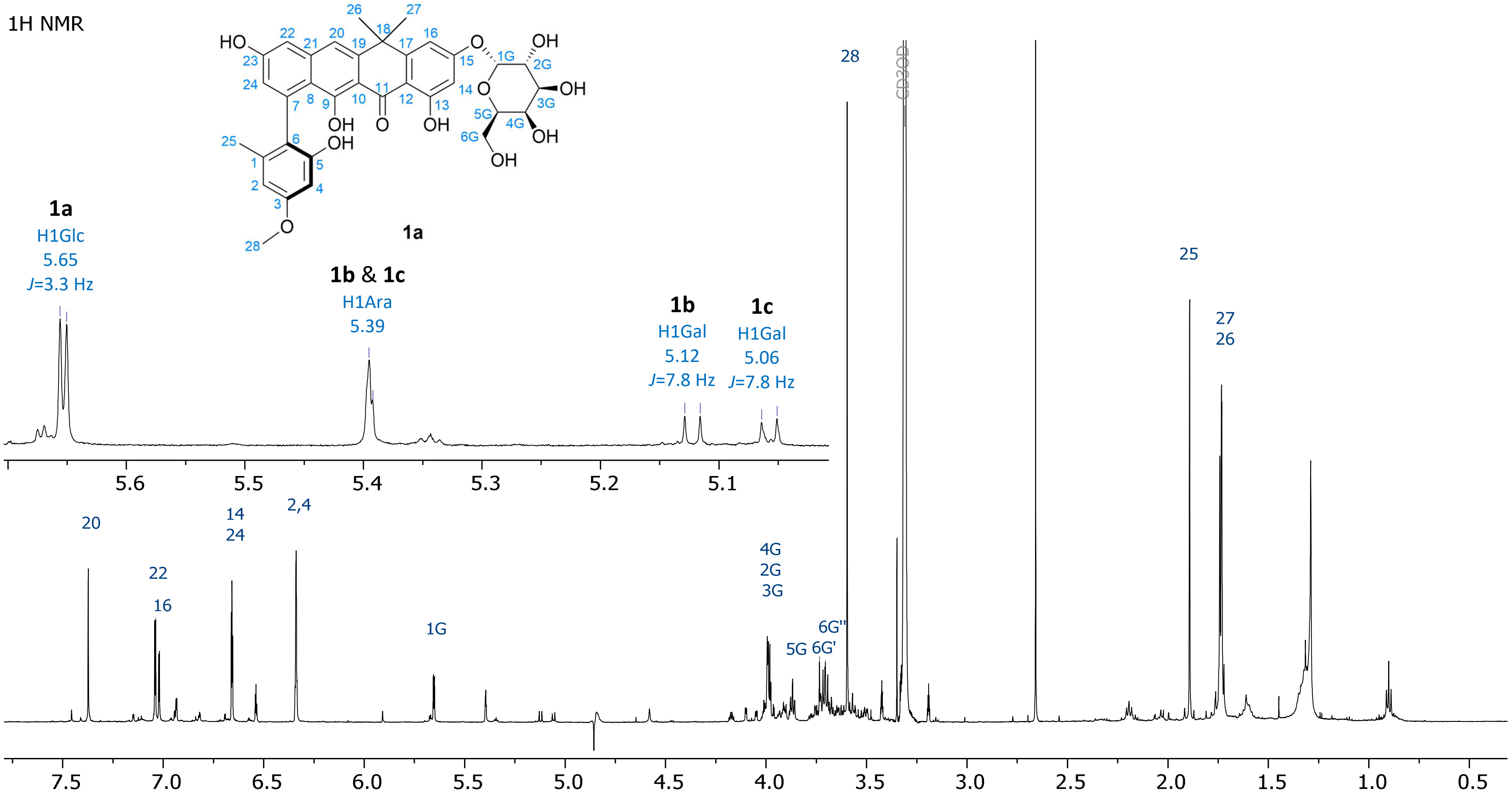

Figure S25 ^{1}H NMR spectrum (CD_3OD, 600 MHz, 298K) of fasamycin glycosides of Sample 1. Assignment of resonances of compound **1a** is shown on the main spectrum. Insert: expansion of the region showing provisional assignment of anomeric signals of disaccharide glycosides **1b** and **1c**.

13C

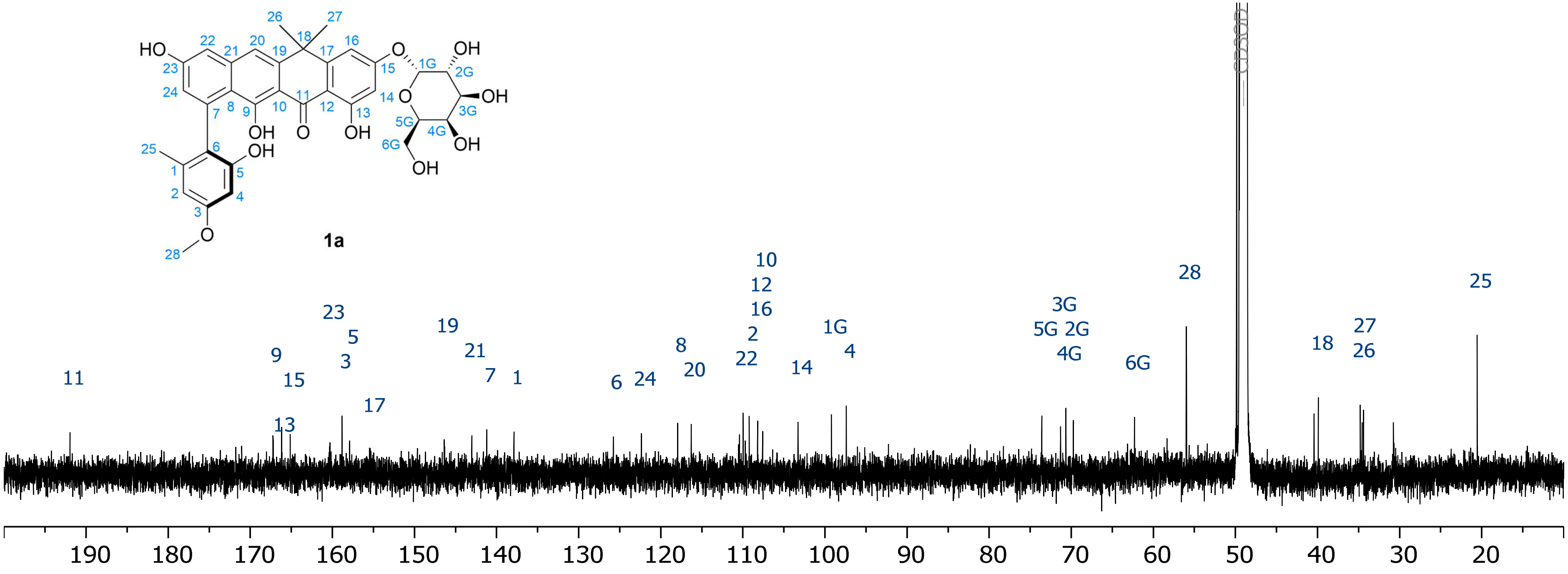

Figure S26 ^{13}C NMR spectrum (CD_3OD, 150 MHz, 298K) of fasamycin glycosides of sample 1. Assignment of resonances of compound **1a** is shown.

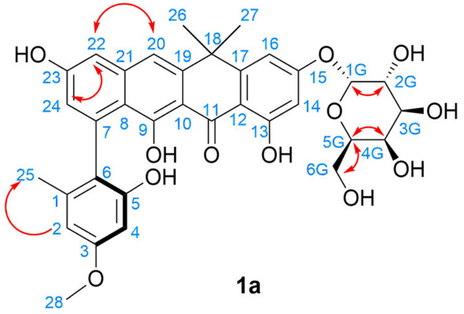

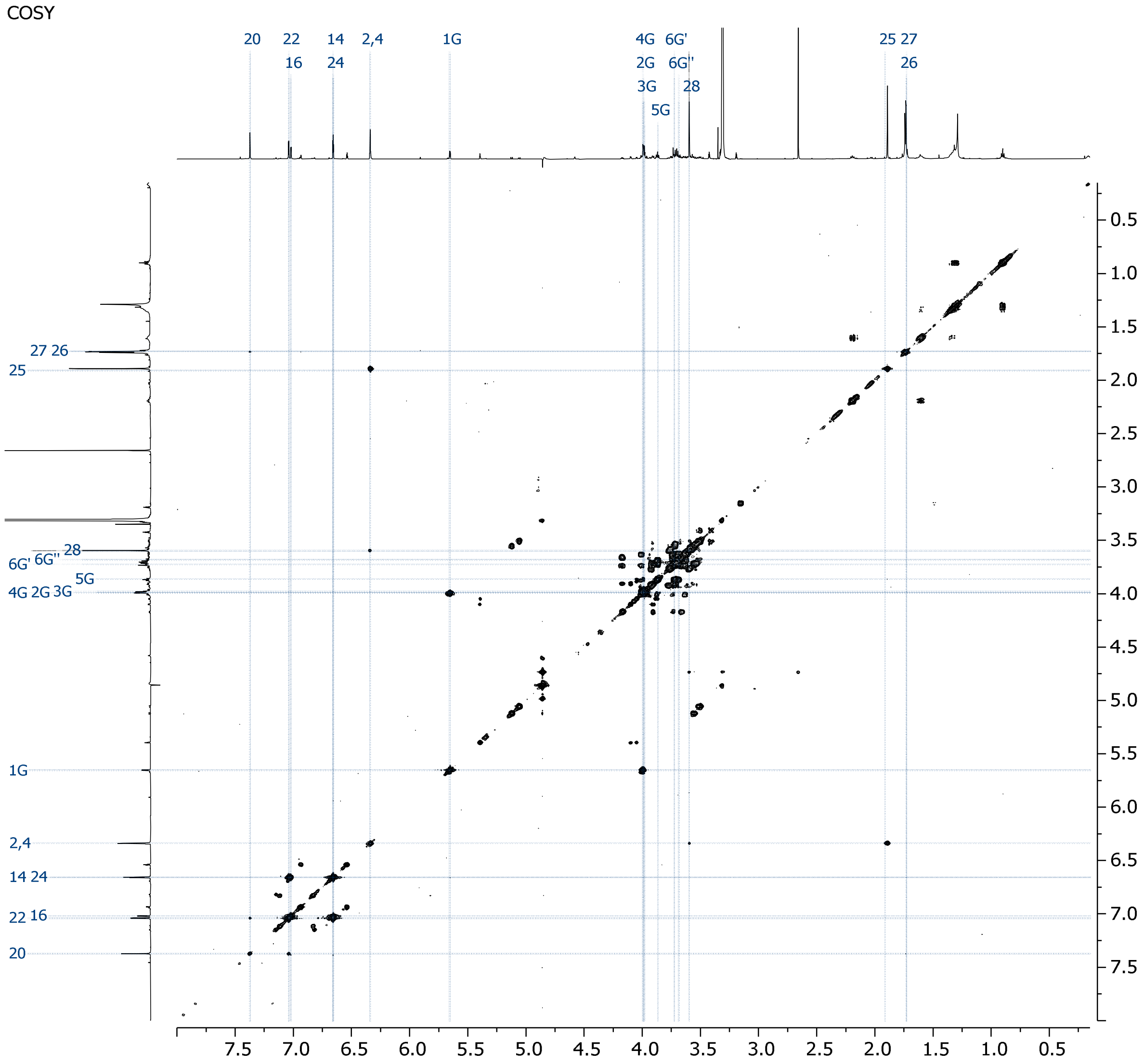

Figure S27 ^{1}H-^{1}H COSY spectrum (CD_3OD, 298K) of fasamycin glycosides of sample 1. Assignment of resonances of compound **1a** is shown.

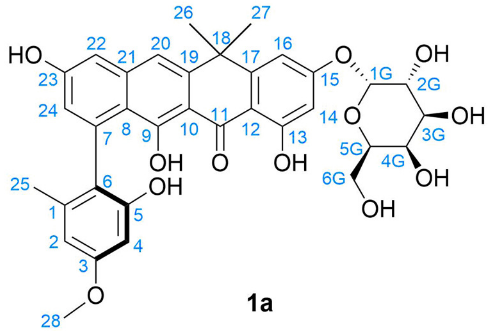

1a

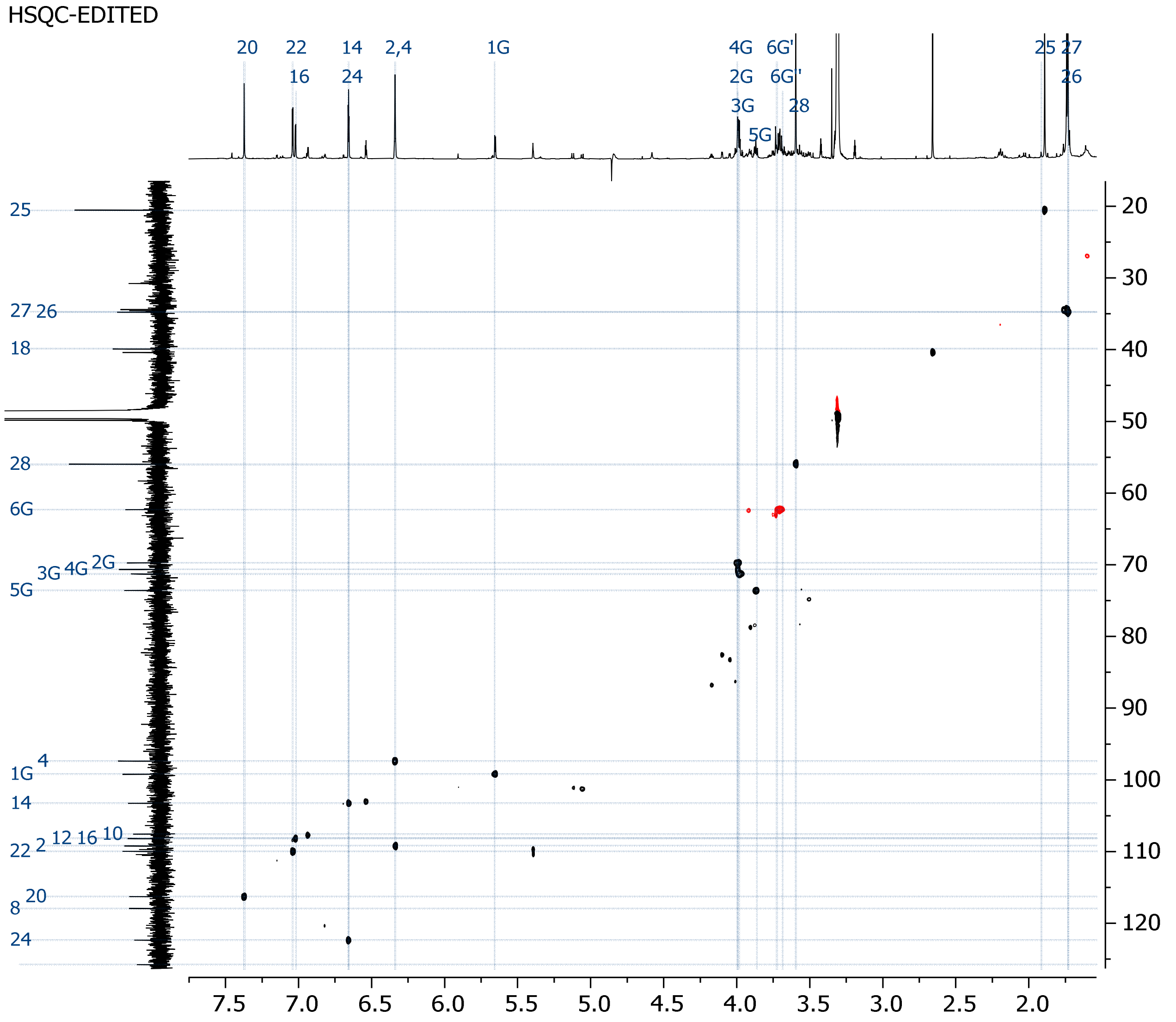

Figure S28 ^{1}H-^{13}C HSQC-edited spectrum (CD_3OD, 298K) of fasamycin glycosides of sample 1. Assignment of resonances of compound **1a** is shown.

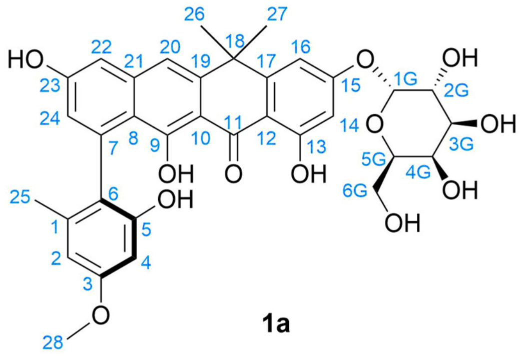

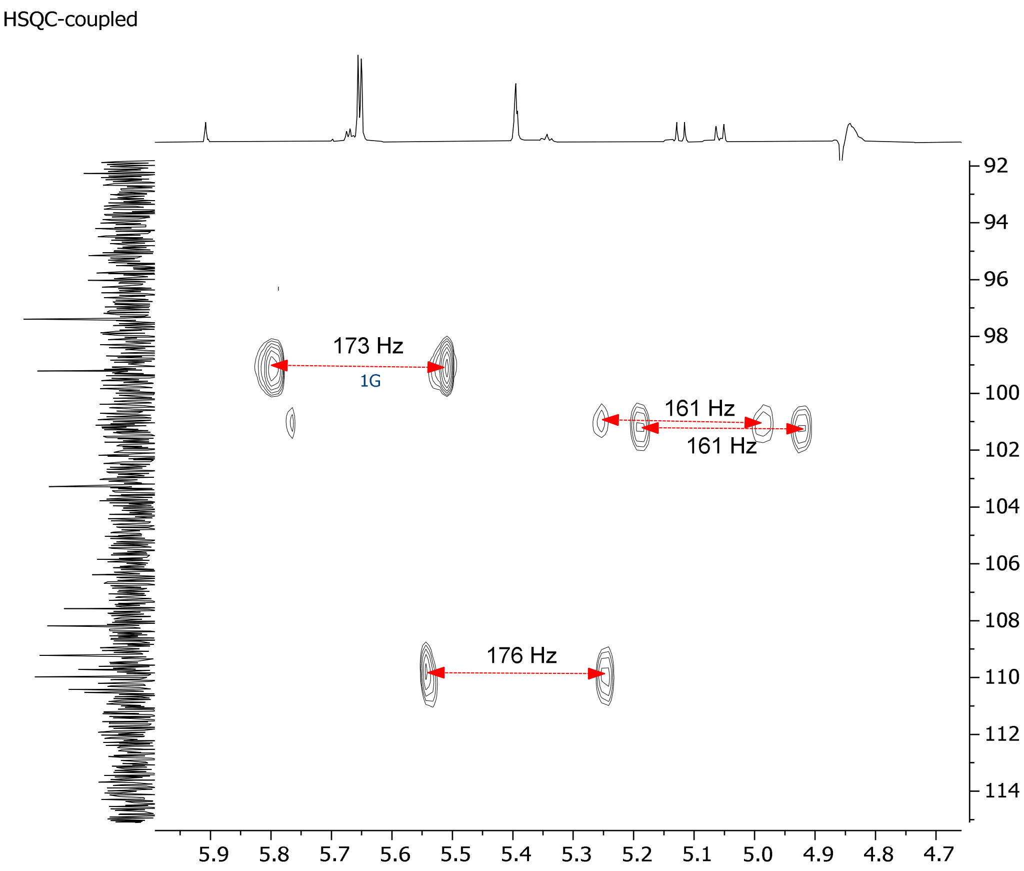

Figure S29 Anomeric region of coupled ^{1}H-^{13}C HSQC spectrum (CD_3OD, 298K) of fasamycin glycosides of sample 1. The size of $^{1}J_{C1Glc,H1Glc}$ coupling constants is measured along F2 dimension.

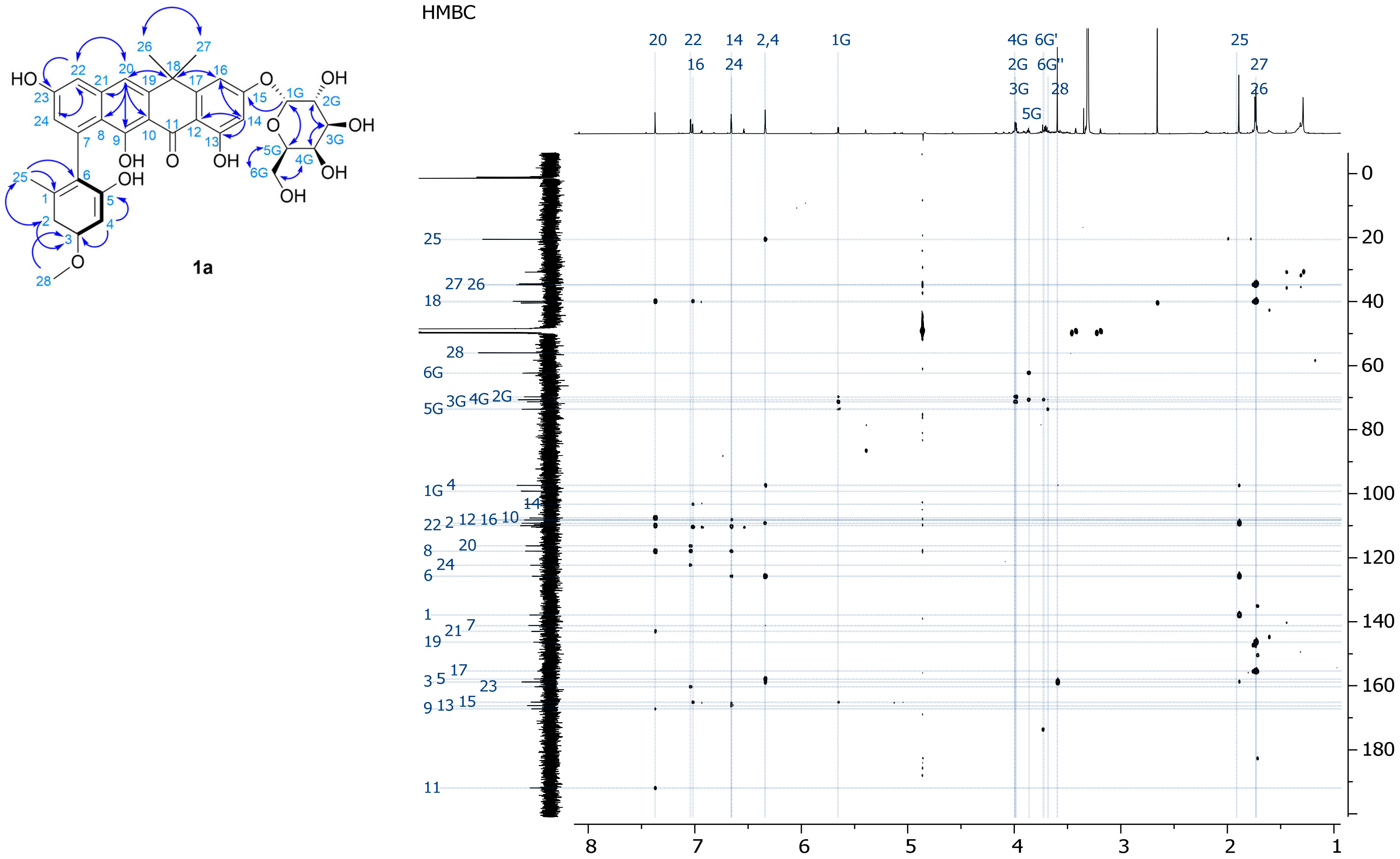

Figure S30 ^{1}H-^{13}C HMBC spectrum (CD_3OD, 298K) of fasamycin glycosides of sample 1. Assignment of resonances of compound **1a** is shown.

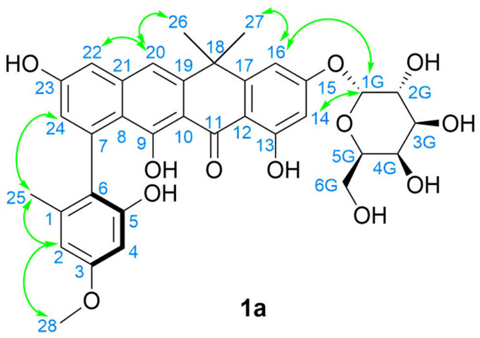

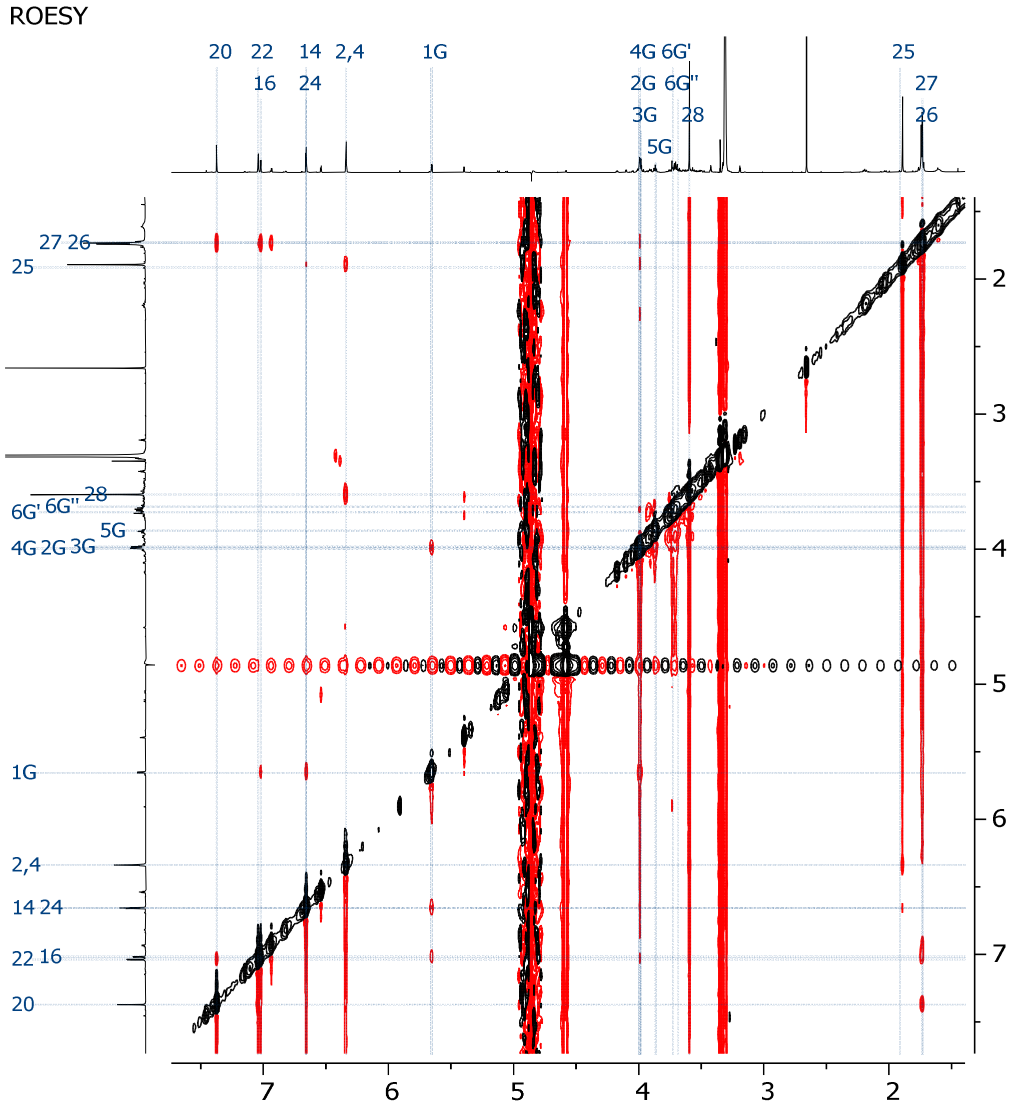

Figure S31 ^{1}H-^{1}H ROESY spectrum (CD$_3$OD, 298K) of fasamycin glycosides of sample 1. Assignment of resonances of compound **1a** is shown.

Table S3 Resonances assignment in ^{1}H and ^{13}C NMR spectra of compound 1a

No	δ_H	δ_C	COSY	HSQC-EDITED	HMBC	ROESY
**1**	-	137.9	-	-	-	-
**2**	6.34	109.2	25	2	3, 4, 6, 25	25, 28
**3**	-	158.8	-	-	-	-
**4**	6.34	97.4	-	4	2, 3, 5, 6	-
**5**	-	157.9	-	-	-	-
**6**	-	125.8	-	-	-	-
**7**	-	141.2	-	-	-	-
**8**	-	118	-	-	-	-
**9**	-	167.2	-	-	-	-
**10**	-	107.6	-	-	-	-
**11**	-	192.0	-	-	-	-
**12**	-	108.2	-	-	-	-
**13**	-	166.3	-	-	-	-
**14**	6.66	103.3	-	14	12, 13	1G
**15**	-	165.1	-	-	-	-
**16**	7.02	108.2	-	-	14,18	1G, 26, 27
**17**	-	155.3	-	-	-	-
**18**	-	39.9	-	-	-	-
**19**	-	146.4	-	-	-	-
**20**	7.37	116.3	22	20	8, 9, 11, 18, 21, 22	22, 26, 27
**21**	-	143	-	-	-	-
**22**	7.04	110	20,24	22	20, 23, 24	20
**23**	-	160.3	-	-	-	-
**24**	6.66	122.3	22	24	22	25
**25**	1.91	20.6	2	25	1, 2, 6	2, 24
**26**	1.73	34.8	-	26	18, 27	16, 20
**27**	1.73	34.7	-	-	26	16, 20
**28**	3.6	56.0	-	-	3	2
**1G**	5.66	99.2	2G	1G	2G, 3G, 5G, 15	14, 16
**2G**	3.99	69.7	1G	-	-	-
**3G**	3.98	71.3	-	-	2G	-
**4G**	3.99	70.7	5G	-	3G	-
**5G**	3.86	73.6	4G, 6'G, 6''G	-	4G, 6G	-
**6G'**	3.73	62.3	5G	-	4G	-
**6G''**	3.68	62.3	5G	-	5G	-

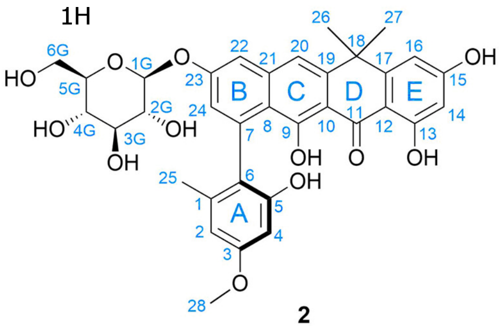

2

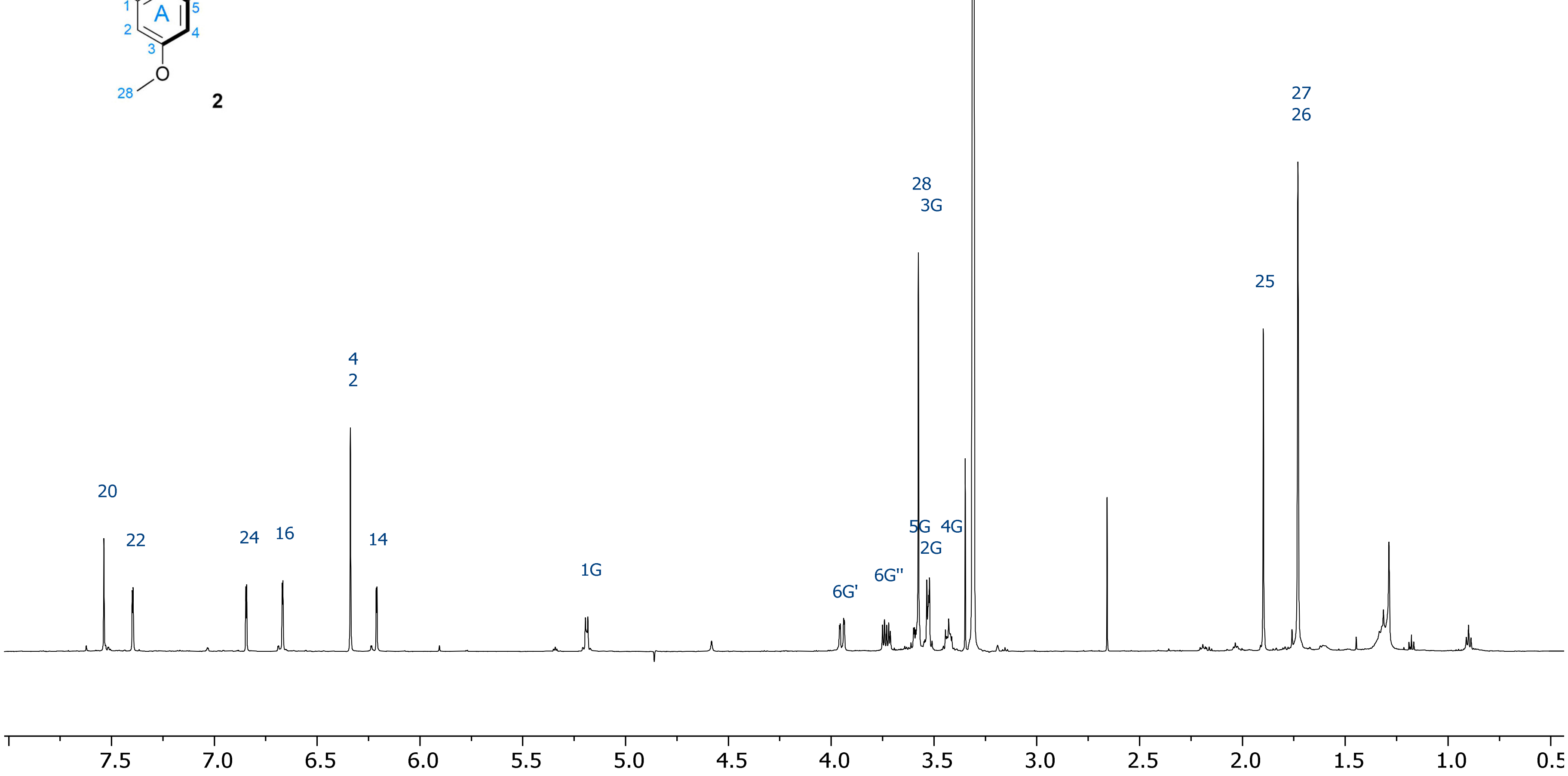

Figure S32 ^{1}H NMR spectrum (CD_3OD, 600 MHz, 298K) of fasamycin glycoside **2**

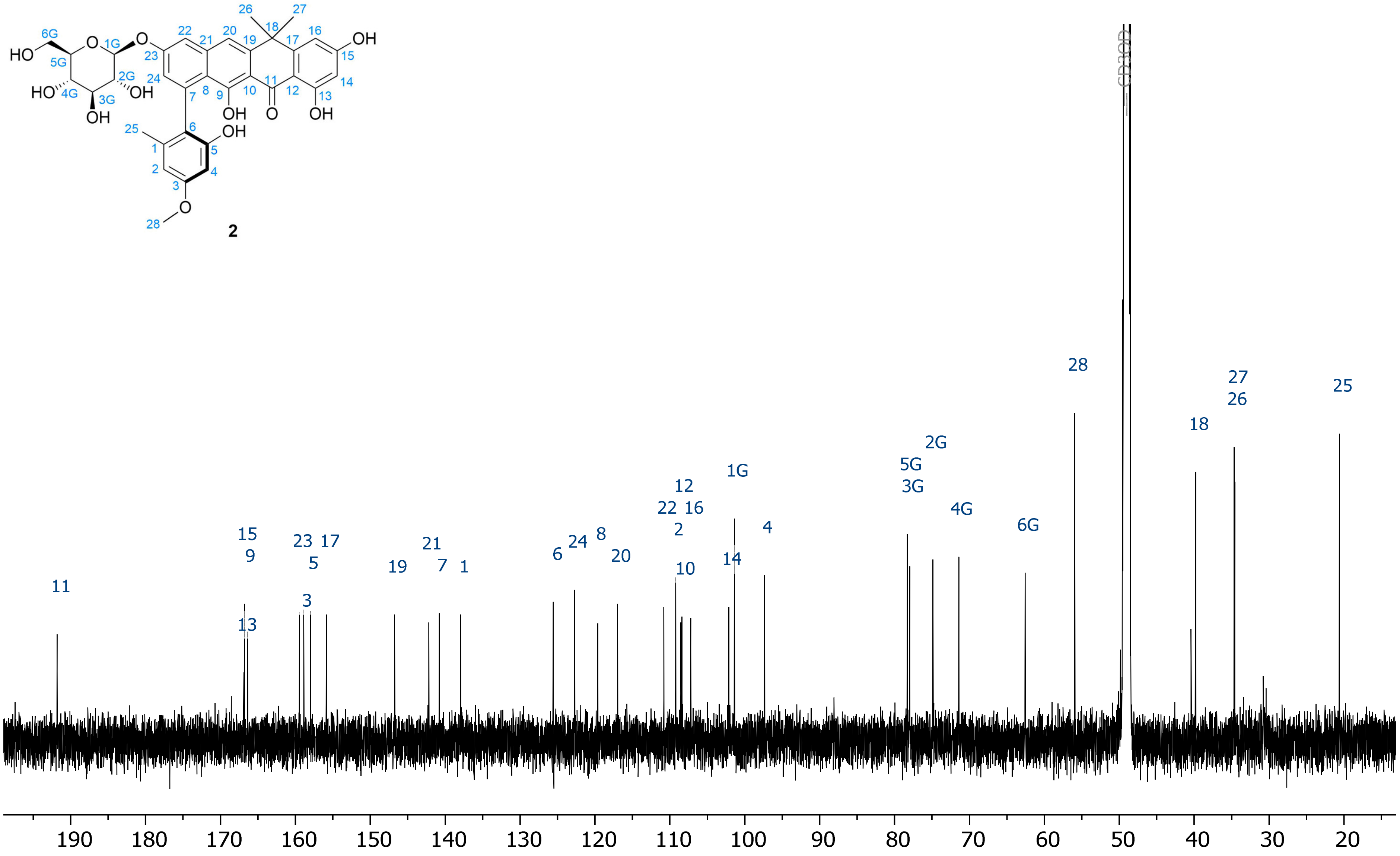

Figure S33 ^{13}C NMR spectrum (CD_3OD, 150 MHz, 298K) of fasamycin glycoside **2**

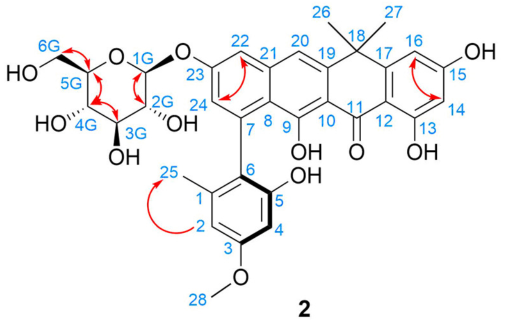

2

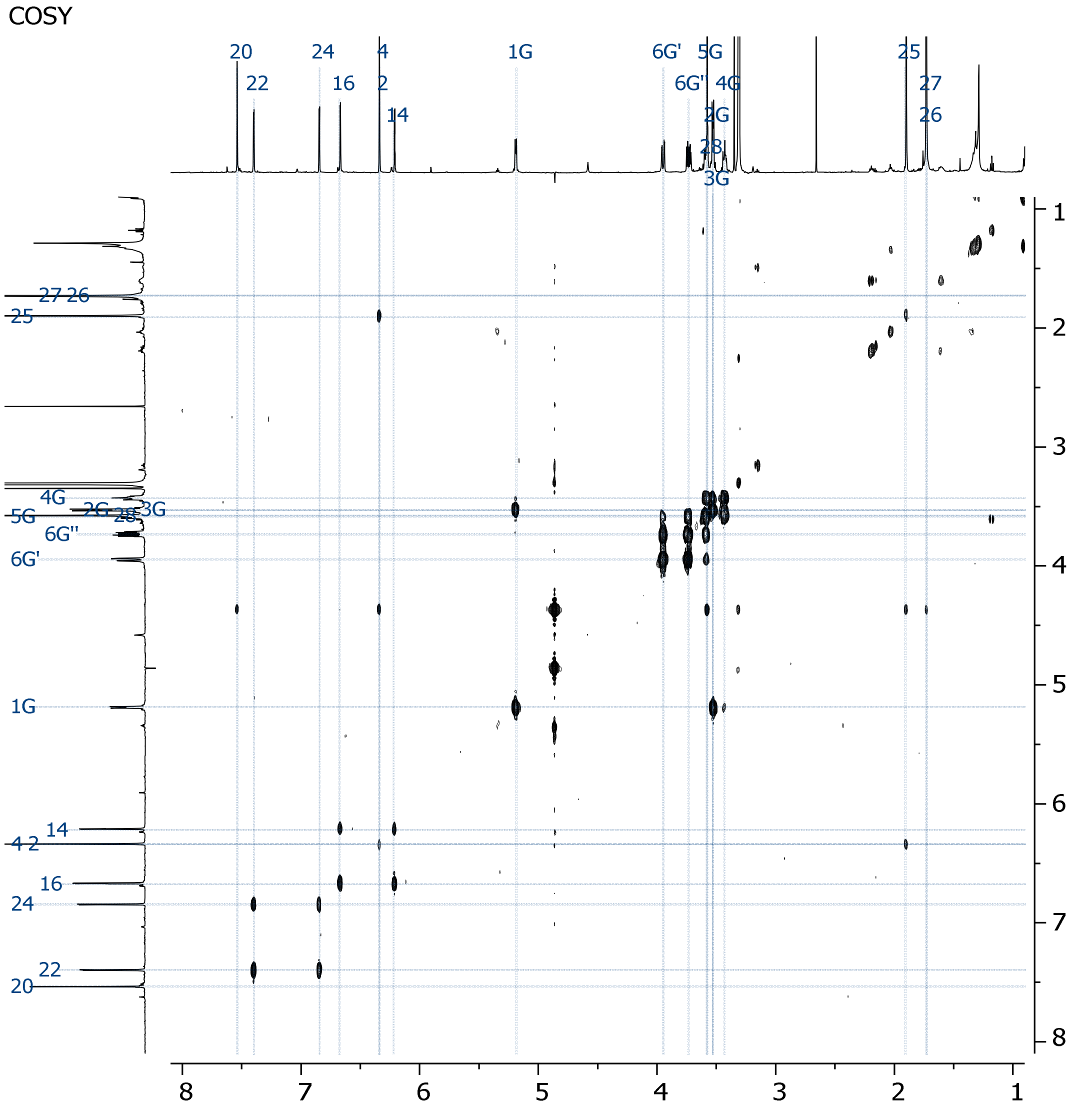

Figure S34 ^1H-^1H COSY spectrum (CD_3OD, 298K) of fasamycin glycoside **2**

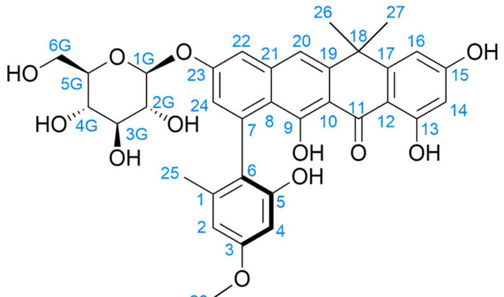

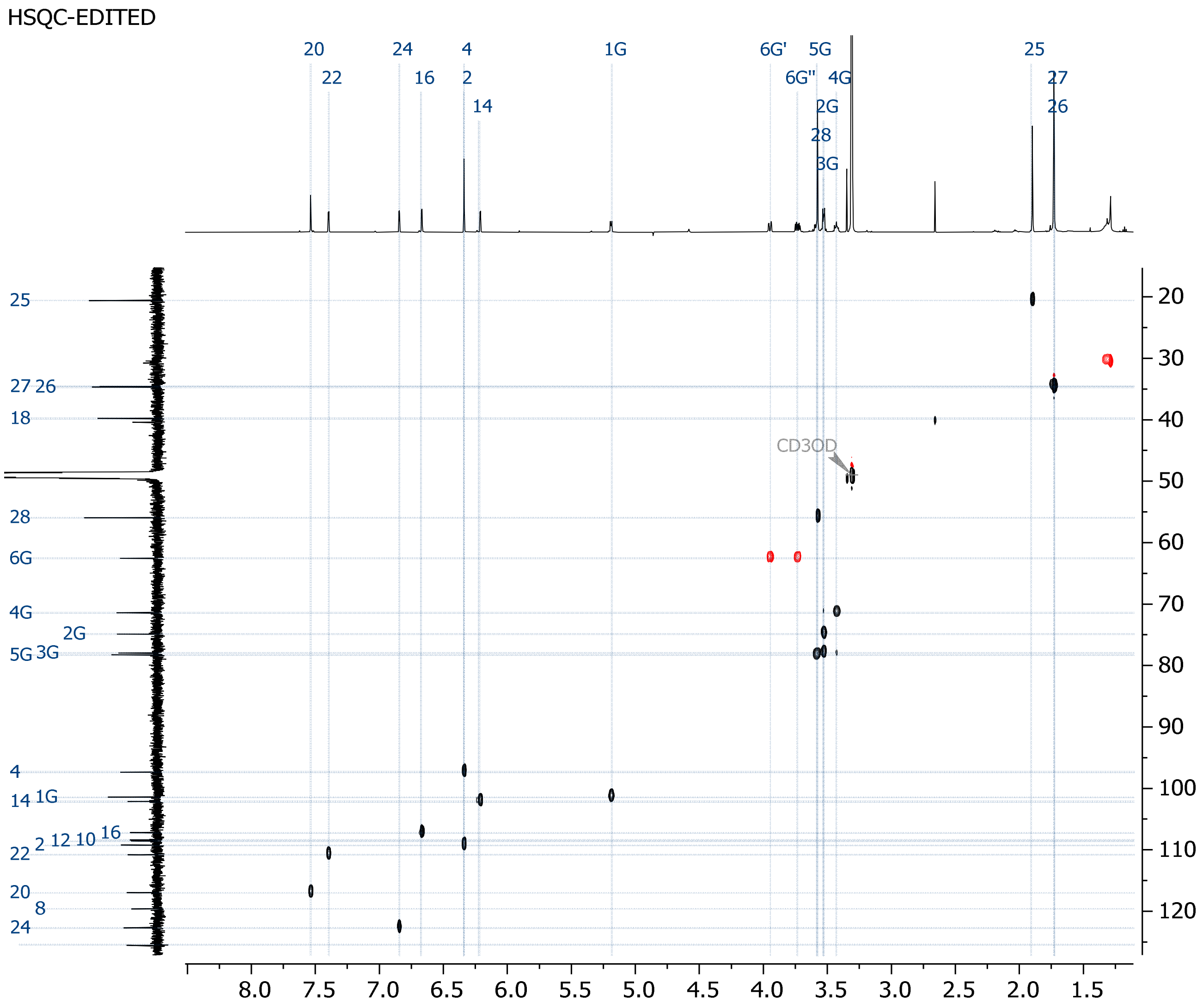

Figure S35 ^{1}H-^{13}C HSQC-edited spectrum (CD_3OD, 298K) of fasamycin glycoside **2**

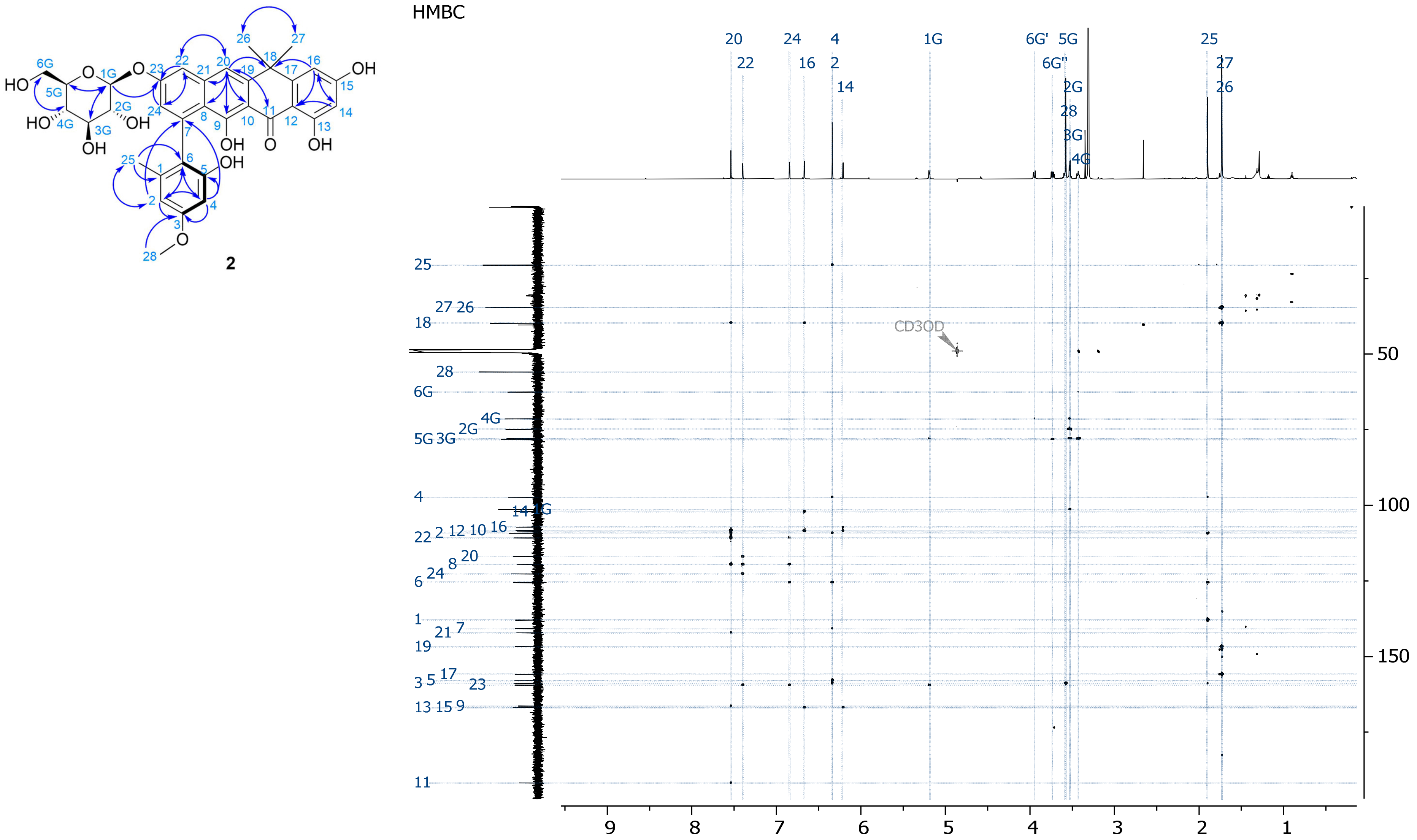

Figure S36 ^{1}H-^{13}C HMBC spectrum (CD_3OD, 298K) of fasamycin glycoside **2**

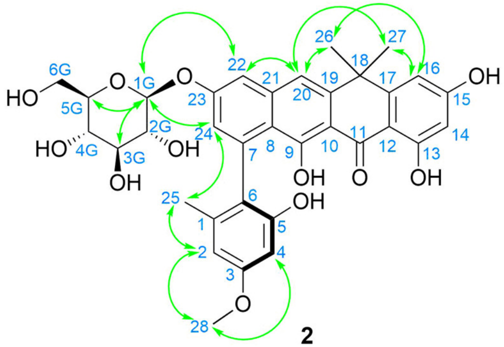

2

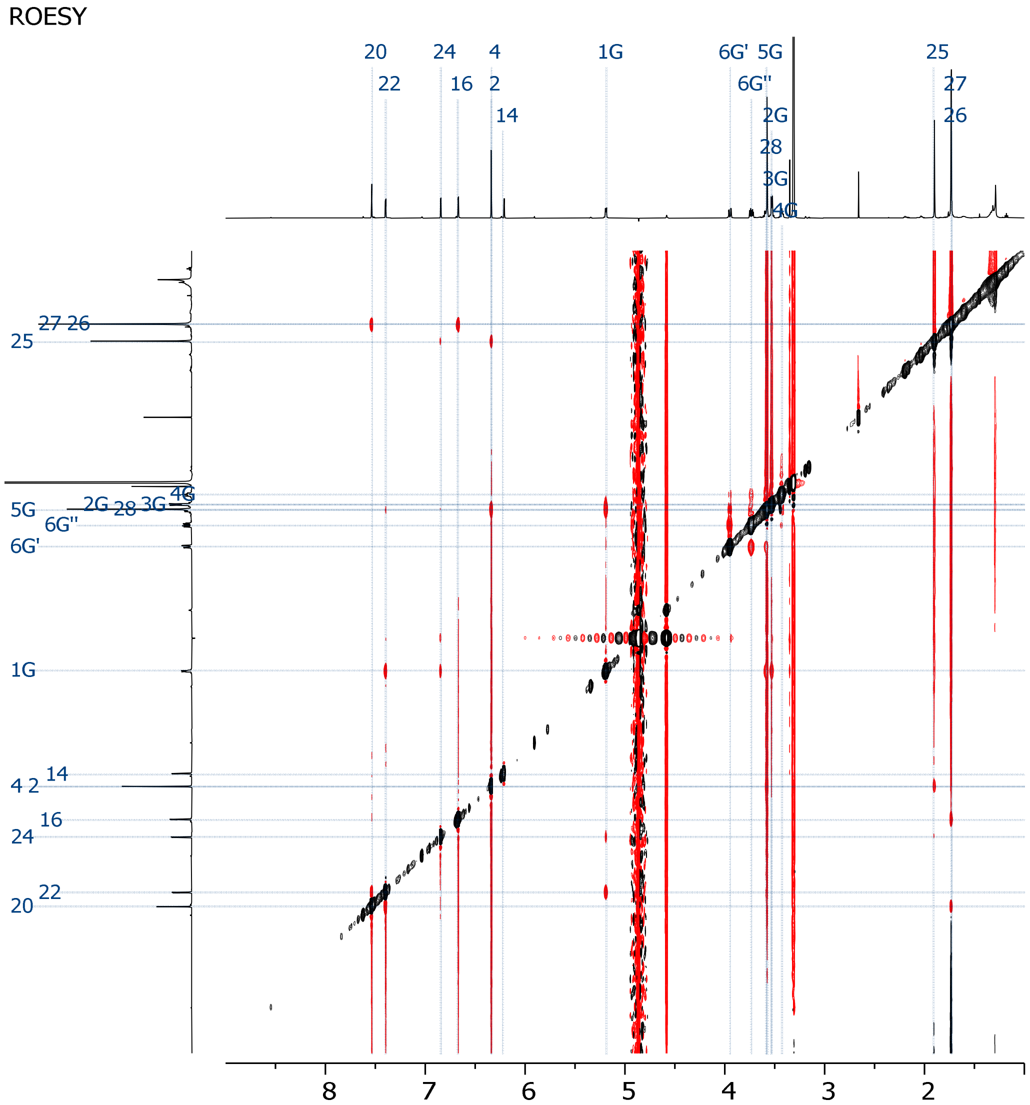

Figure S37 ^{1}H-^{1}H ROESY spectrum (CD_3OD, 600 MHz, 298K) of fasamycin glycoside **2**

Table S4 Resonances assignment in ^{1}H and ^{13}C NMR spectra of compound 2

No	δ_H	δ_C	COSY	HSQC-EDITED	HMBC	ROESY
**1**	-	137.8	-	-	-	-
**2**	6.34	109.2	25	2	3, 4, 6, 7, 25	25, 28
**3**	-	158.9	-	-		-
**4**	6.34	97.4	-	-	2, 3, 4, 5, 7	28
**5**	-	158.0	-	-	-	-
**6**	-	125.4	-	-	-	-
**7**	-	140.8	-	-	-	-
**8**	-	119.6	-	-	-	-
**9**	-	166.4	-	-	-	-
**10**	-	108.4	-	-	-	-
**11**	-	191.7	-	-	-	-
**12**	-	108.6	-	-	-	-
**13**	-	166.9	-	-	-	-
**14**	6.22	102.2	16	14	12, 13, 15, 16	-
**15**	-	166.8	-	-	-	-
**16**	6.67	107.2	14	16	14, 18	26, 27
**17**	-	155.9	-	-	-	-
**18**	-	39.8	-	-	-	-
**19**	-	146.8	-	-	-	-
**20**	7.54	117.0	-	20	8, 9, 10, 11, 18, 21, 22	22, 26, 27
**21**	-	142.2	-	-	-	-
**22**	7.4	110.8	24	-	24	1G, 20
**23**	-	159.5	-	-	-	-
**24**	6.84	122.7	22	-	22, 23	1G, 25
**25**	1.91	20.6	2	25	1, 2, 3, 6	2, 24
**26**	1.73	34.7	-	26	17, 18,19, 27	16, 20
**27**	1.73	34.6	-	27	17, 18,19, 26	16, 20
**28**	3.58	55.9	-	-	3	2, 4
**1G**	5.18	101.4	2G	-	23	3G, 5G, 22,24
**2G**	3.53	74.9	1G	-	1G, 3G	-
**3G**	3.53	78.0	4G	3G	2G, 4G, 5G	1G
**4G**	3.43	71.5	3G,5G	4G	2G, 3G	-
**5G**	3.58	78.3	4G, 6G'', 6G'	-	-	1G
**6'G**	3.95	62.6	5G	-	4G	-
**6''G**	3.73	62.6	5G	6G	4G	-

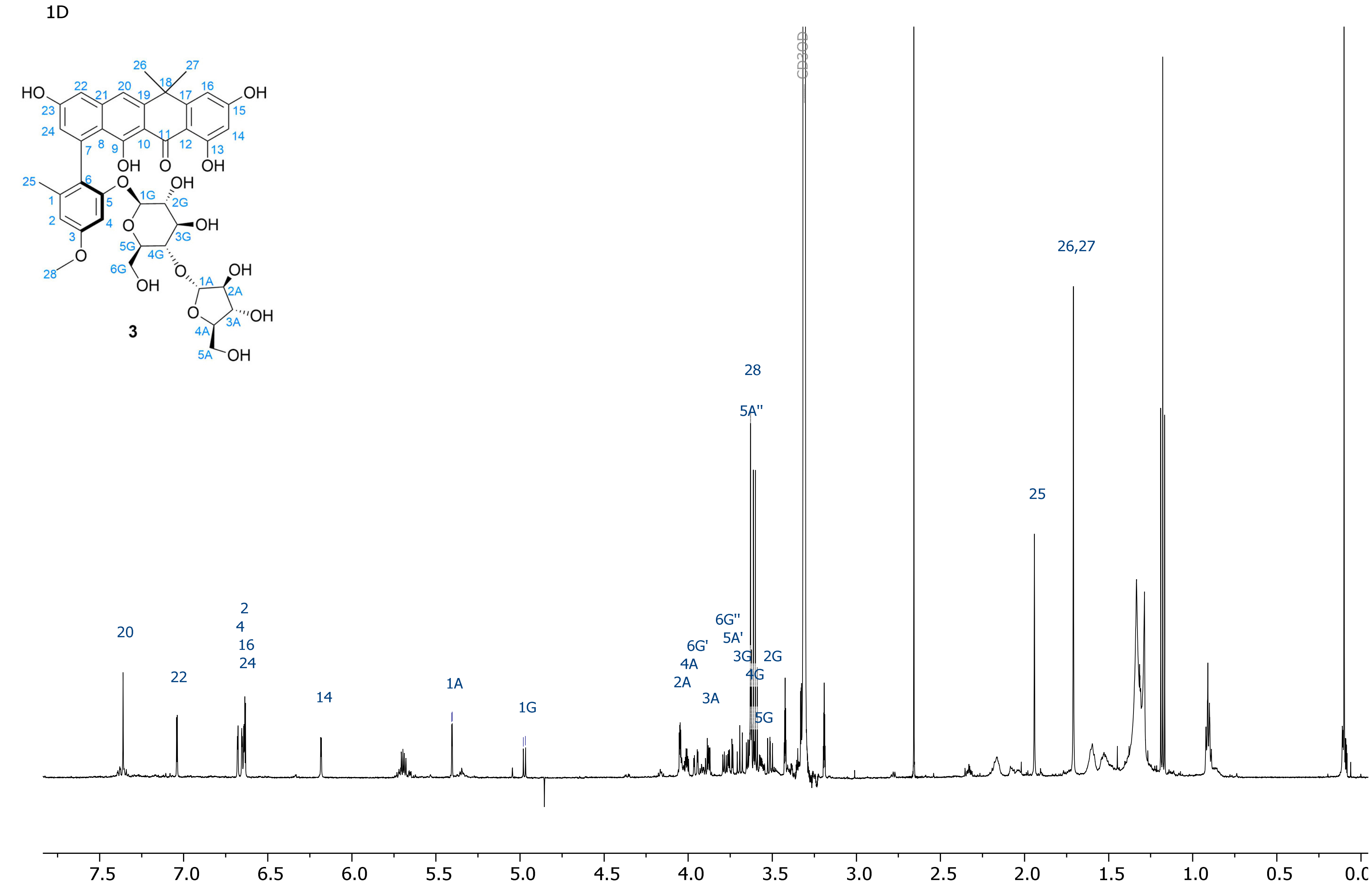

Figure S38 ^{1}H NMR spectrum (CD_3OD, 600 MHz, 298K) of fasamycin glycoside **3**

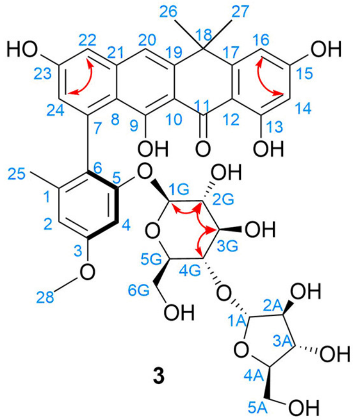

3

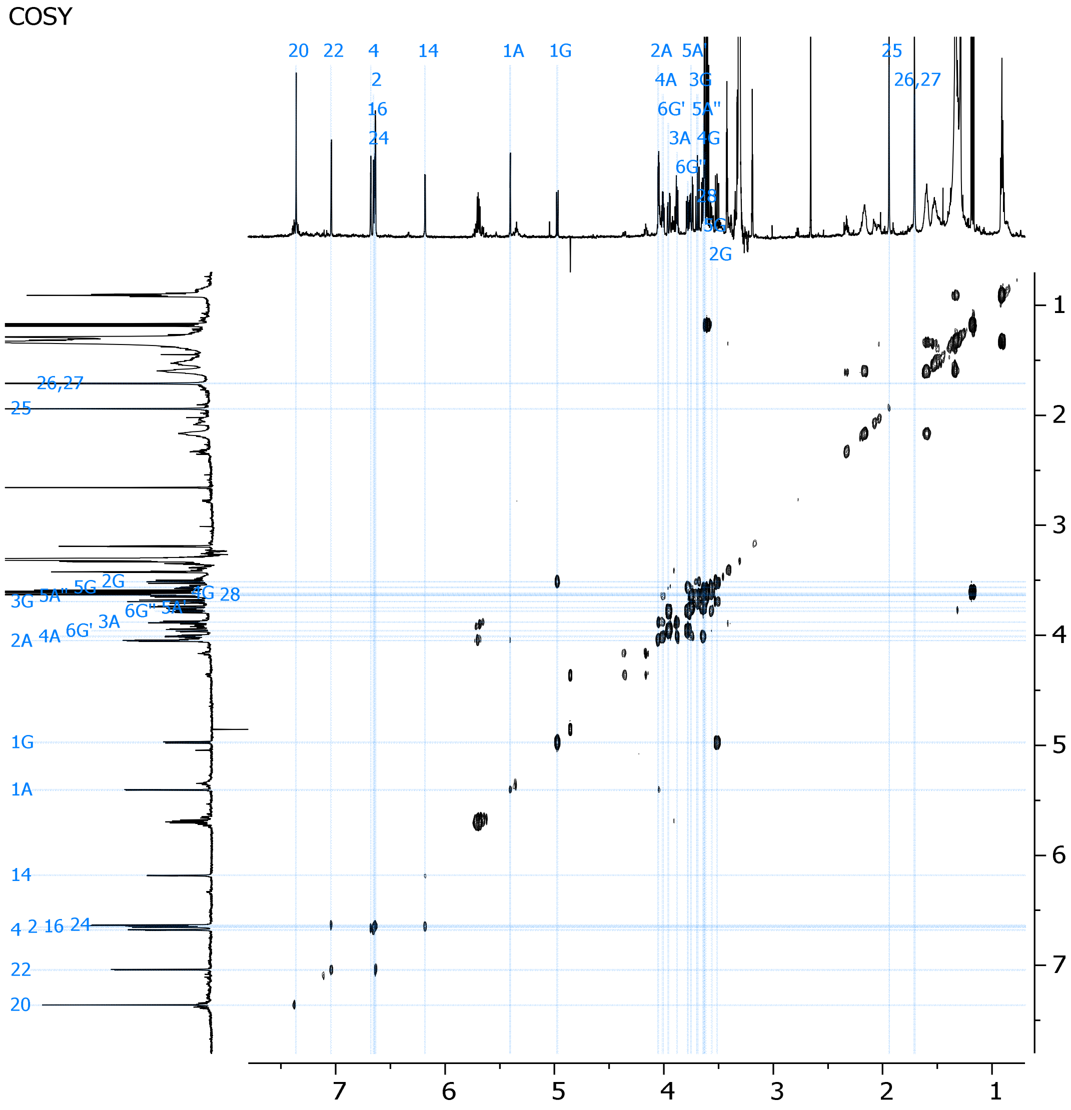

Figure S39 ${}^{1}H$-${}^{1}H$ COSY spectrum (CD_3OD, 298K) of fasamycin glycoside **3**

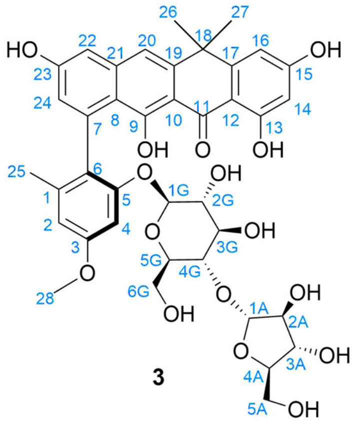

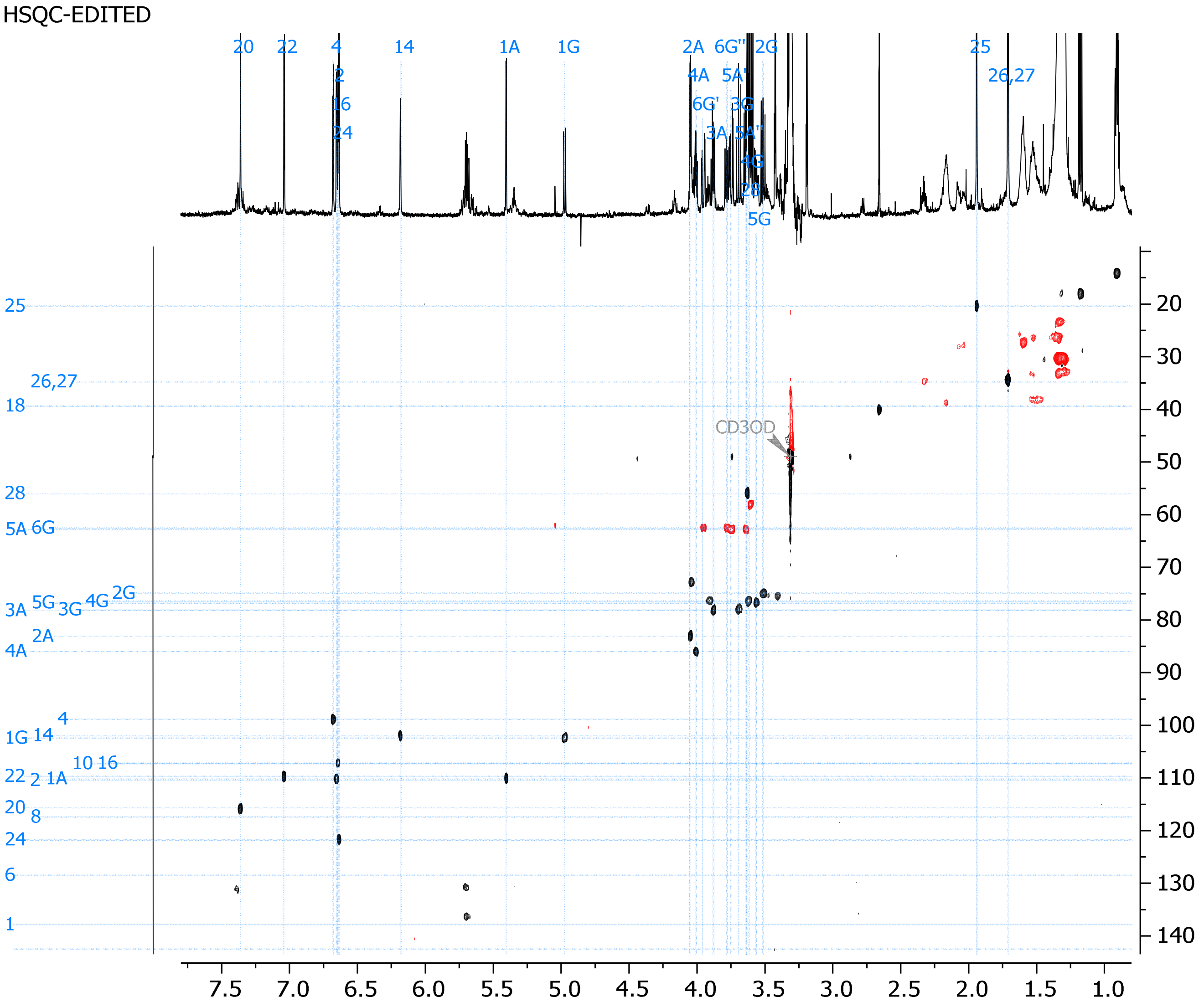

Figure S40 ^{1}H-^{13}C HSQC-edited spectrum (CD_3OD, 298K) of fasamycin glycoside **3**

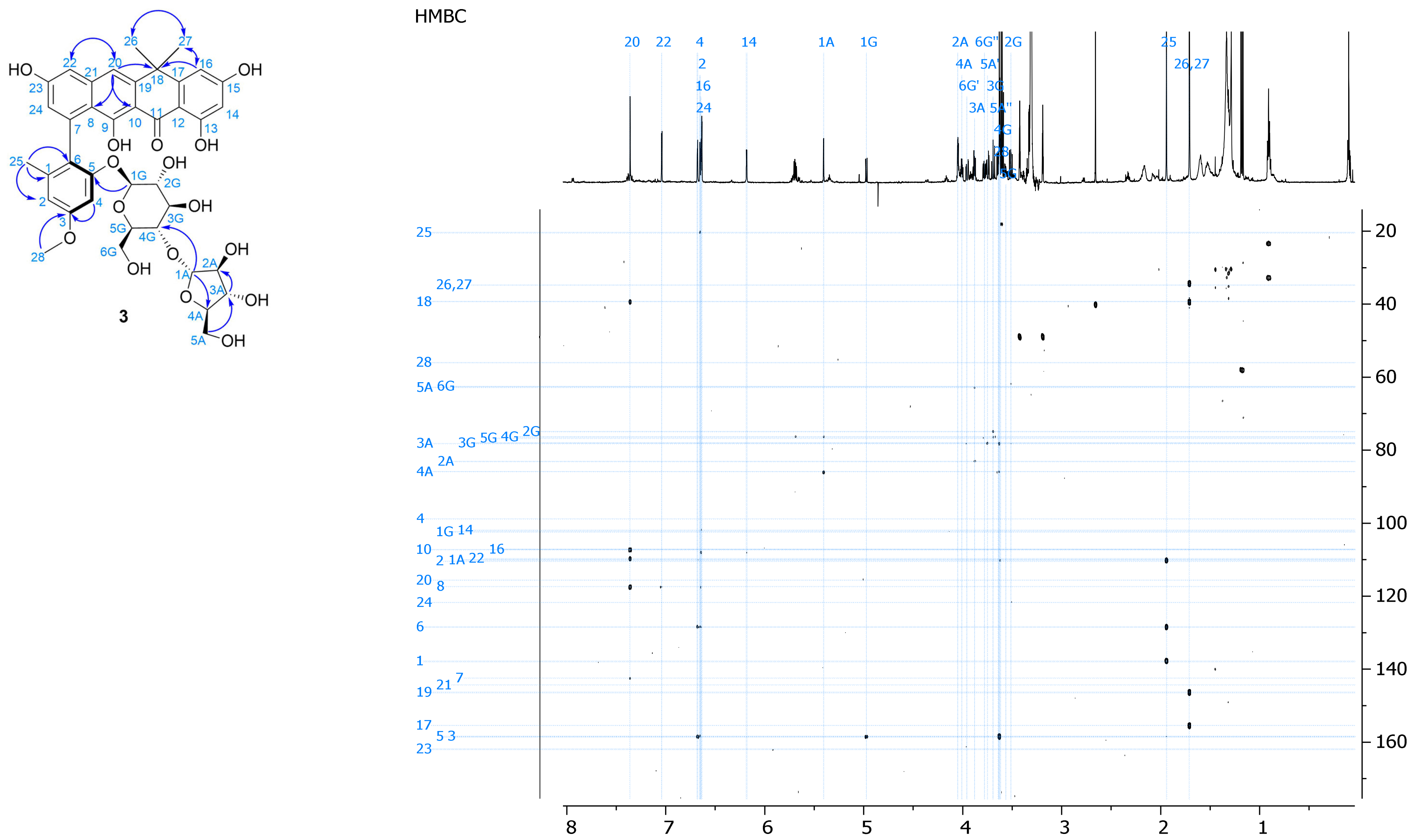

Figure S41 ^{1}H-^{13}C HMBC spectrum (CD_3OD, 298K) of fasamycin glycoside **3**

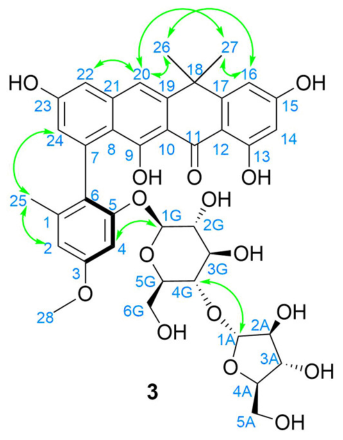

3

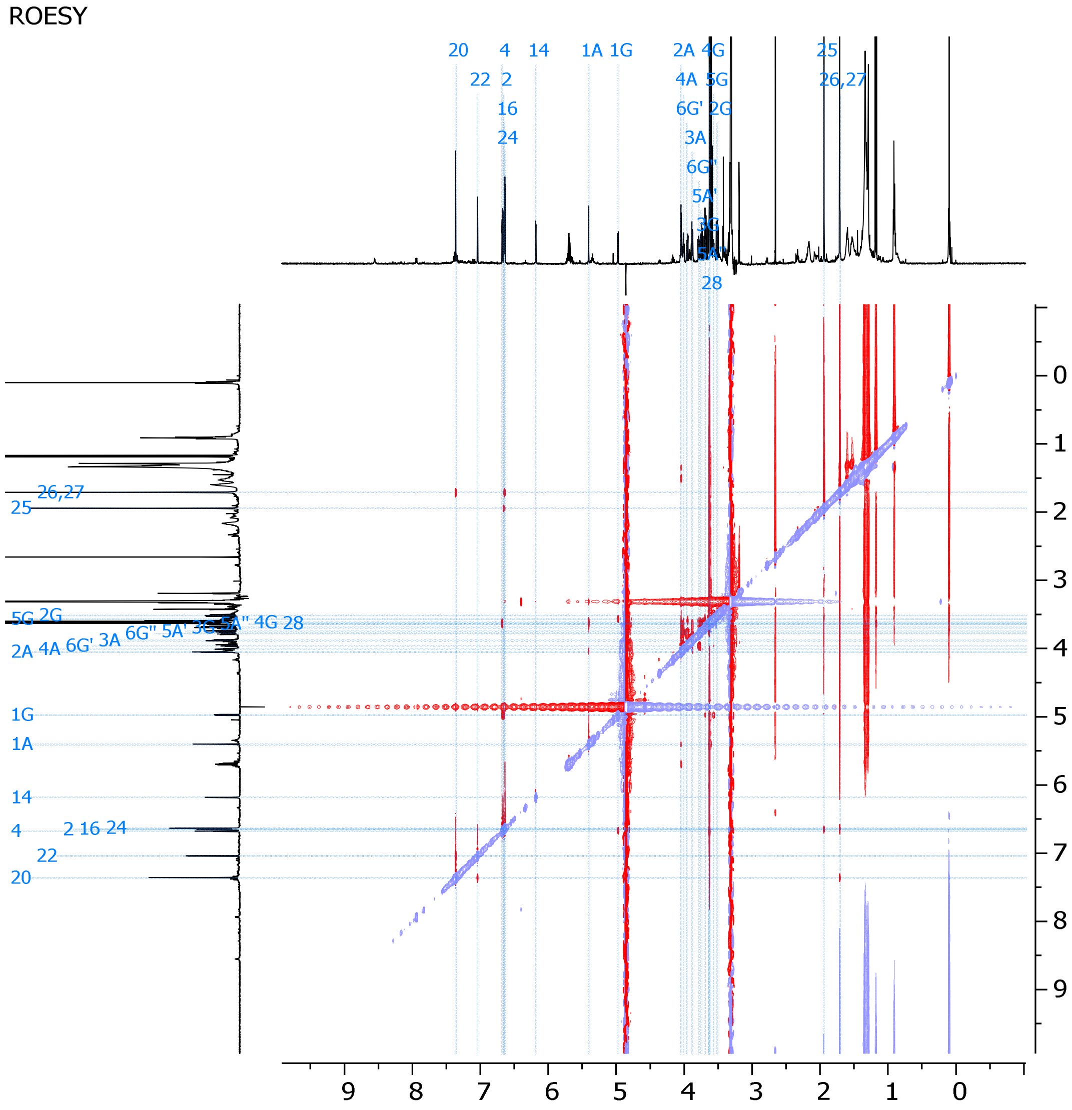

Figure S42 ^{1}H-^{1}H ROESY spectrum (CD_3OD, 298K) of fasamycin glycoside **3**

Table S5 Resonances assignment in ^{1}H and ^{13}C NMR spectra of compound 3

No	δ_H	δ_C*	COSY	HSQC-EDITED	HMBC	ROESY
**1**	-	137.9	-	-	25	-
**2**	6.65	110.4	-	2	25	25
**3**	-	158.4	-	-	4, 28	-
**4**	6.68	98.8	-	4	3,6	G1
**5**	-	158.5	-	-	G1	-
**6**	-	128.5	-	-	4, 25	-
**7**	-	142.5	-	-	-	-
**8**	-	117.4	-	-	20	-
**10**	-	107.3	-	-	-	-
**14**	6.18	102.0	16	14	-	-
**16**	6.65	107.2	14	16	-	26, 27
**17**	-	155.4	-	-	26, 27	-
**18**	-	39.3	-	-	20, 26, 27	-
**19**	-	146.4	-	-	26, 27	-
**20**	7.36	115.7	-	20	8, 10, 18, 22	22, 26, 27
**21**	-	144.4	-	-	-	-
**22**	7.04	109.7	24	22	-	20
**23**	-	161.9	-	-	-	-
**24**	6.64	121.8	22	24	-	25
**25**	1.94	20.4	-	25	1, 2,6	2, 24
**26**	1.71	34.8	-	27	17, 18,19, 27	16, 20
**27**	1.71	34.8	-	26	17, 18,19, 26	16, 20
**28**	3.63	56.0	-	28	3	-
**1A**	5.40	110.1	-	1A	4G, 4A	4G
**2A**	4.05	83.1	-	2A	-	-
**3A**	3.88	78.2	-	3A	F2	-
**4A**	4.01	85.9	-	4A	F5	-
**5'A**	3.75	62.8	-	5A	F3	-
**5''A**	3.64	62.8	-	5A	F3	-
**1G**	4.97	102.4	2G	1G	5	4
**2G**	3.51	74.9	3G, 1G	2G	-	-
**G3**	3.69	78.0	4G, 2G	3G	-	-
**4G**	3.62	76.4	3G	4G	-	1A
**5G**	3.57	76.7	-	5G	-	-
**6'G**	3.96	62.5	-	6G	-	-
**6''G**	3.78	62.5	-	6G	-	-

*Chemical shift values were measured using HSQC and HMBC spectra.

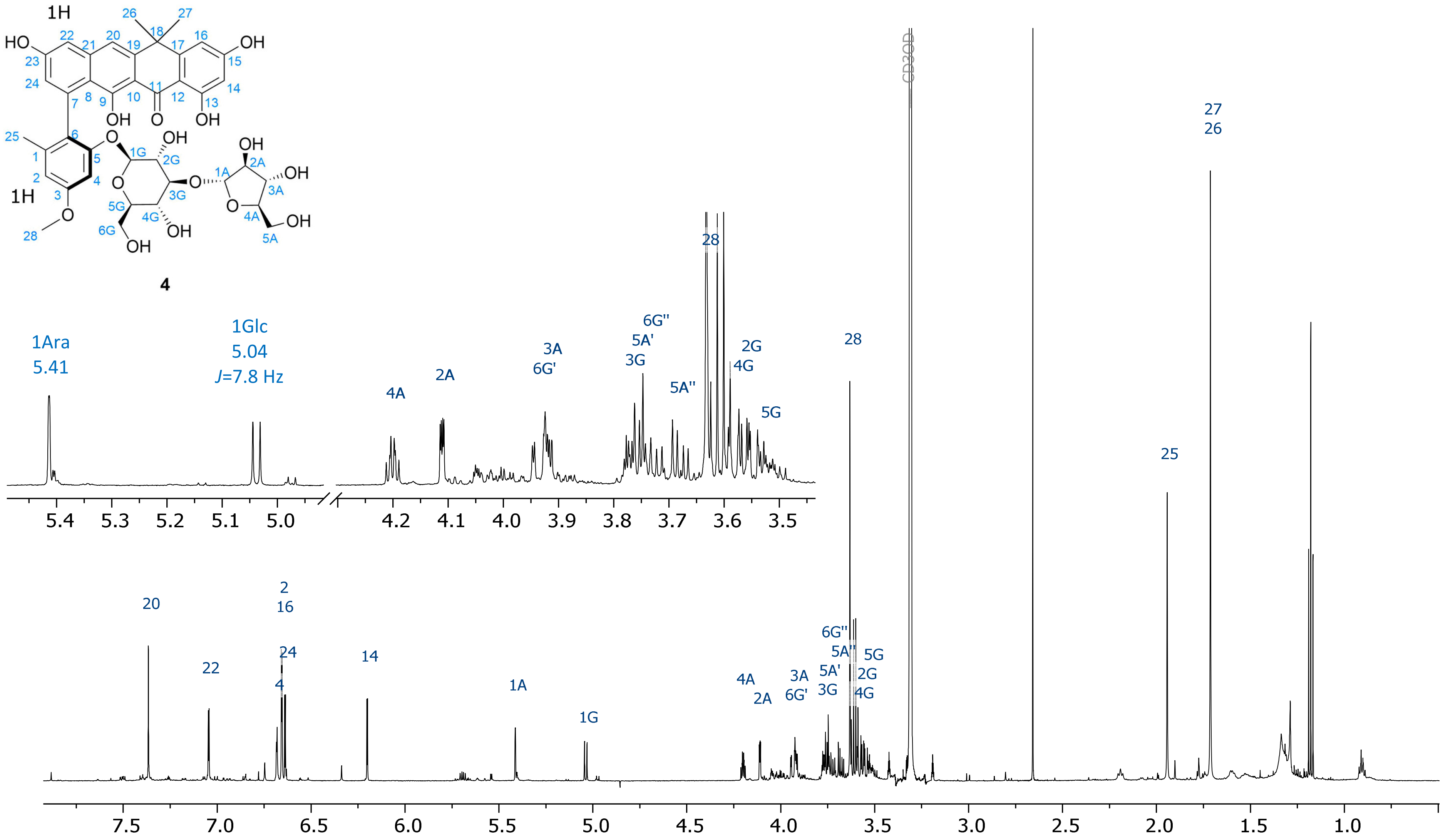

Figure S43 ^{1}H NMR spectrum (CD_3OD, 600 MHz, 298K) of fasamycin glycoside **4**

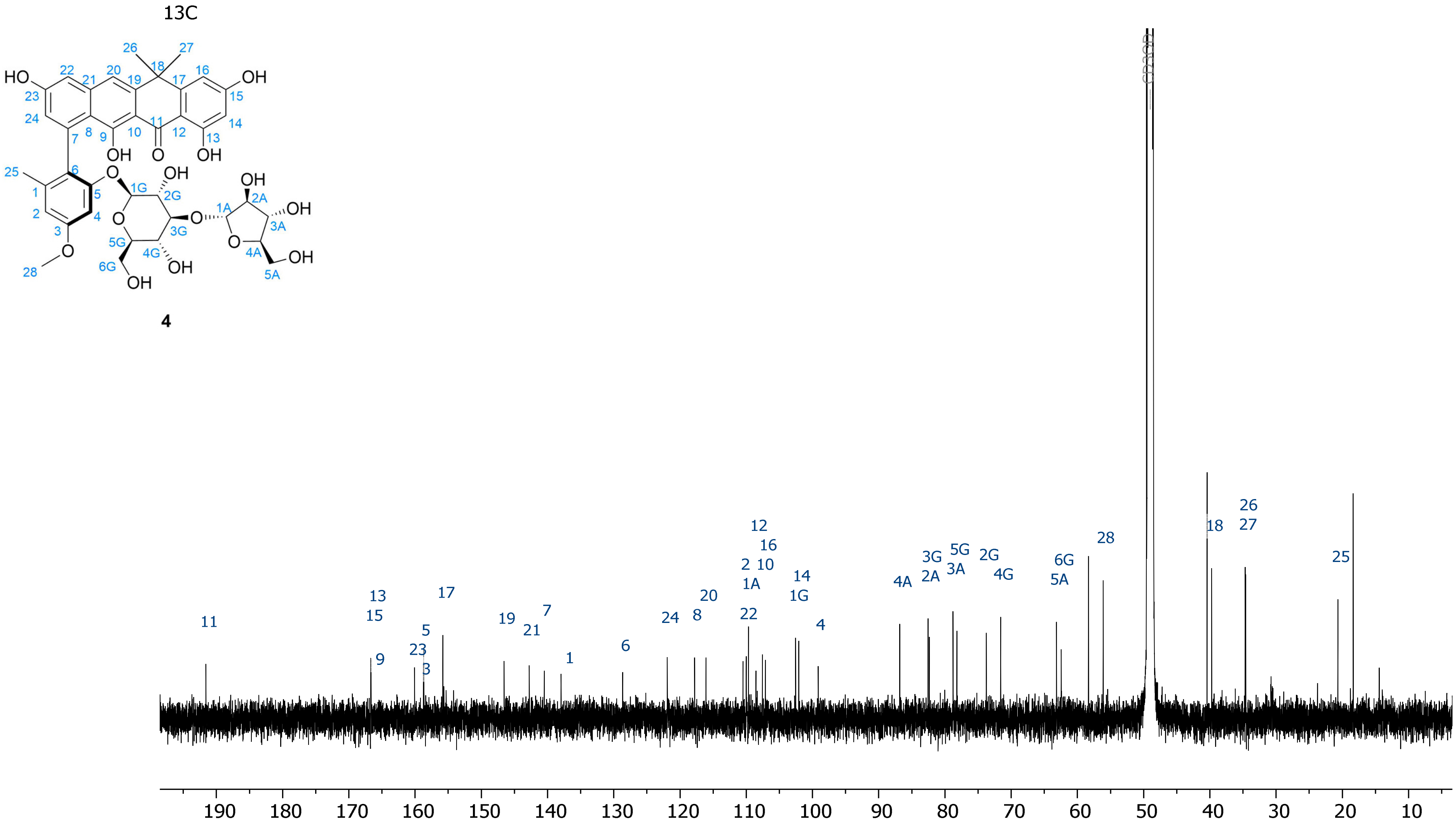

Figure S44 ^{13}C NMR spectrum (CD_3OD, 150 MHz, 298K) of fasamycin glycoside **4**

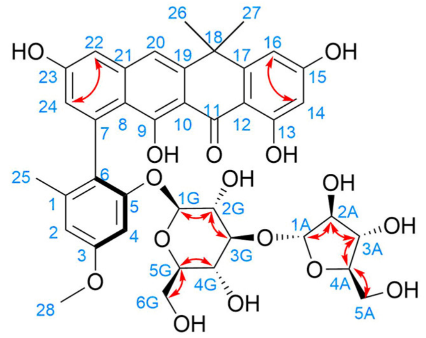

4

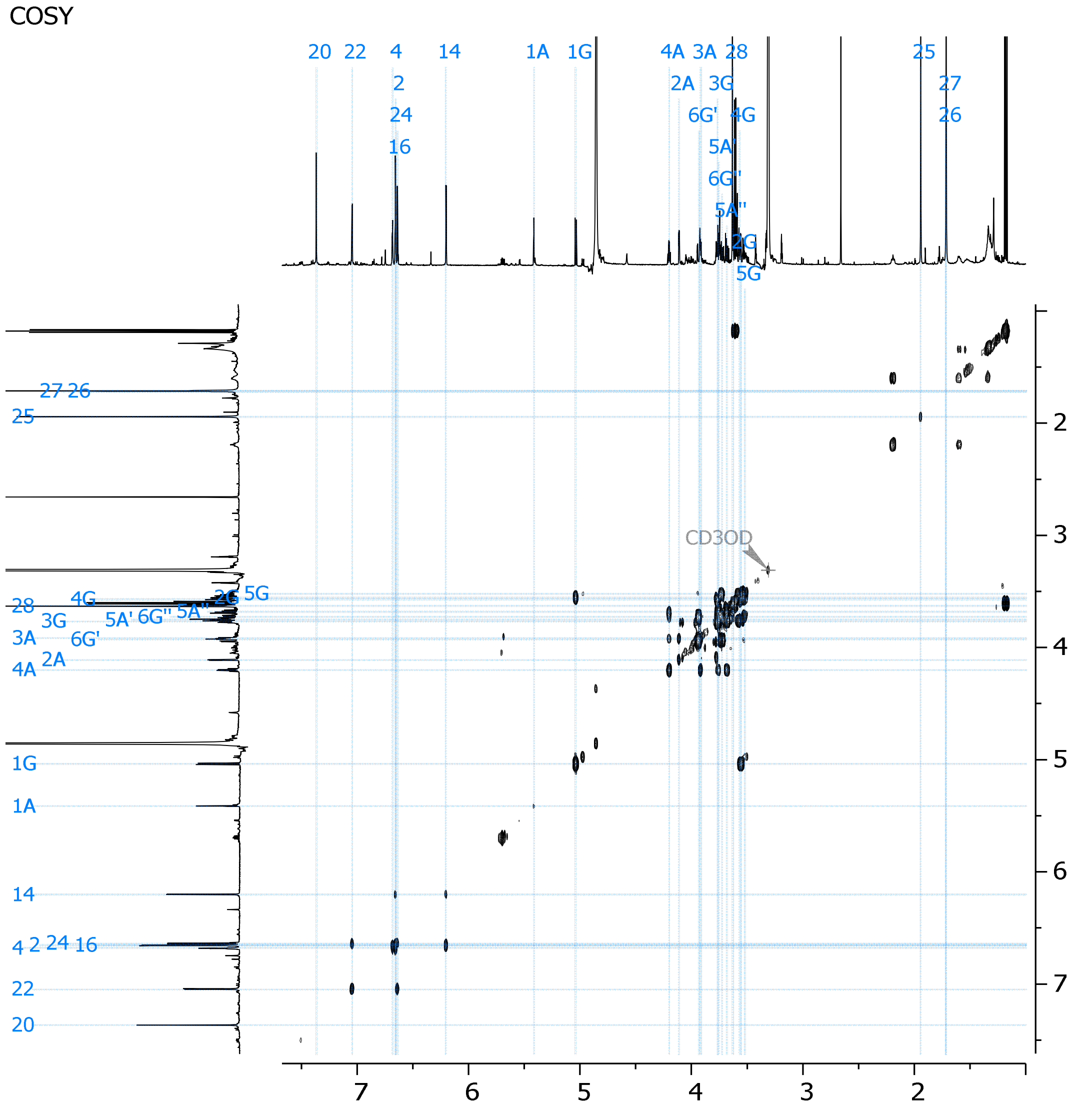

Figure S45 ^1H-^1H COSY spectrum (CD3OD, 298K) of fasamycin glycoside **4**

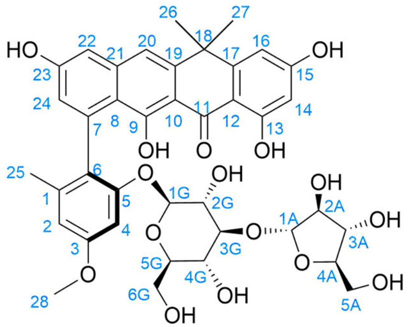

4

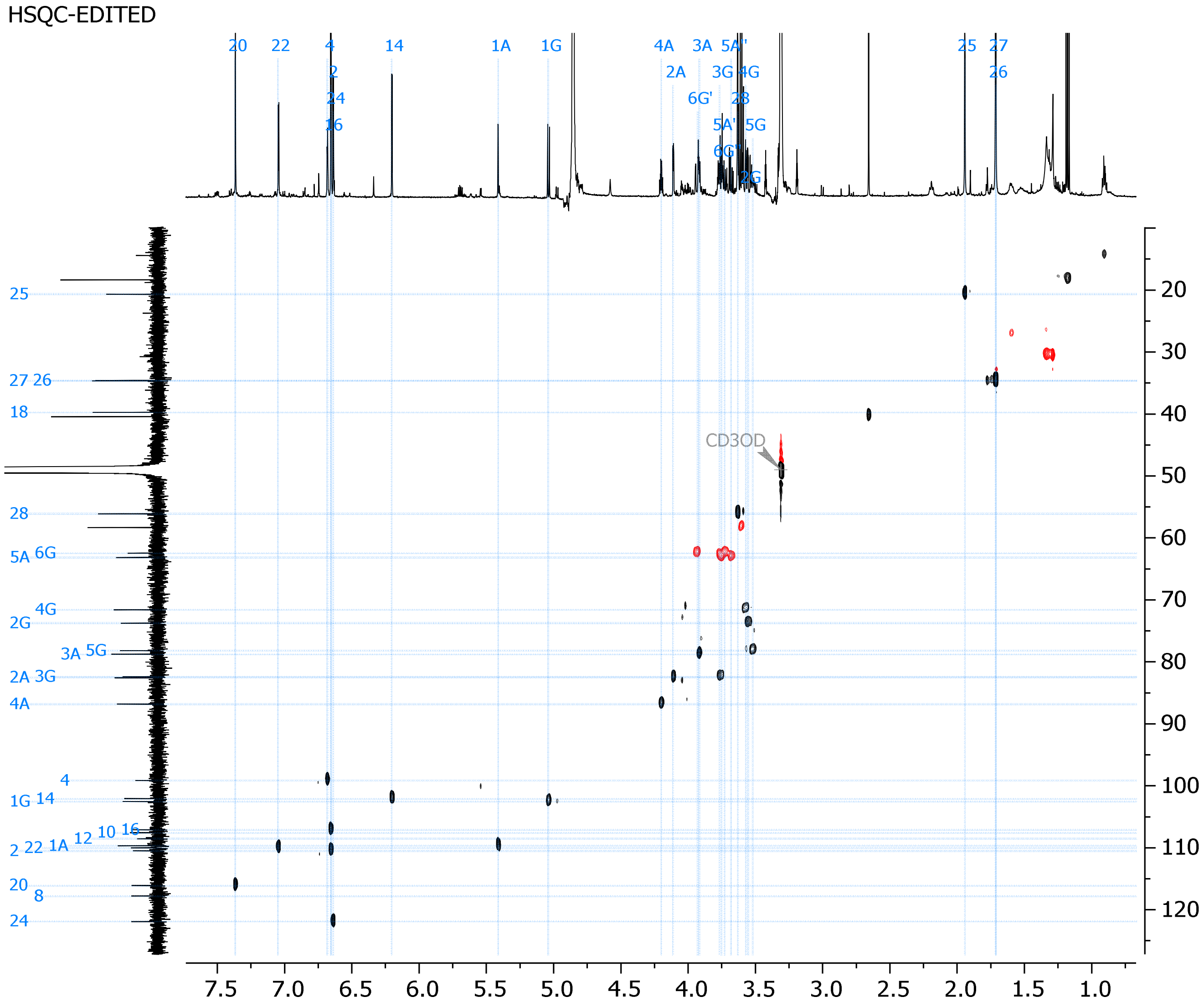

Figure S46 ^{1}H-^{13}C HSQC-edited spectrum (CD_3OD, 298K) of fasamycin glycoside **4**

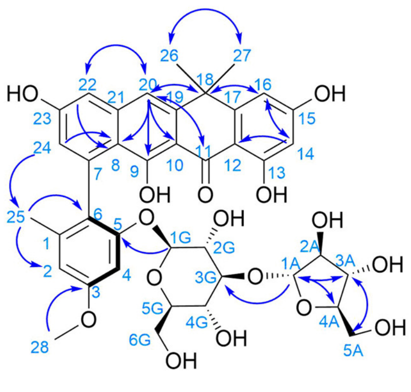

4

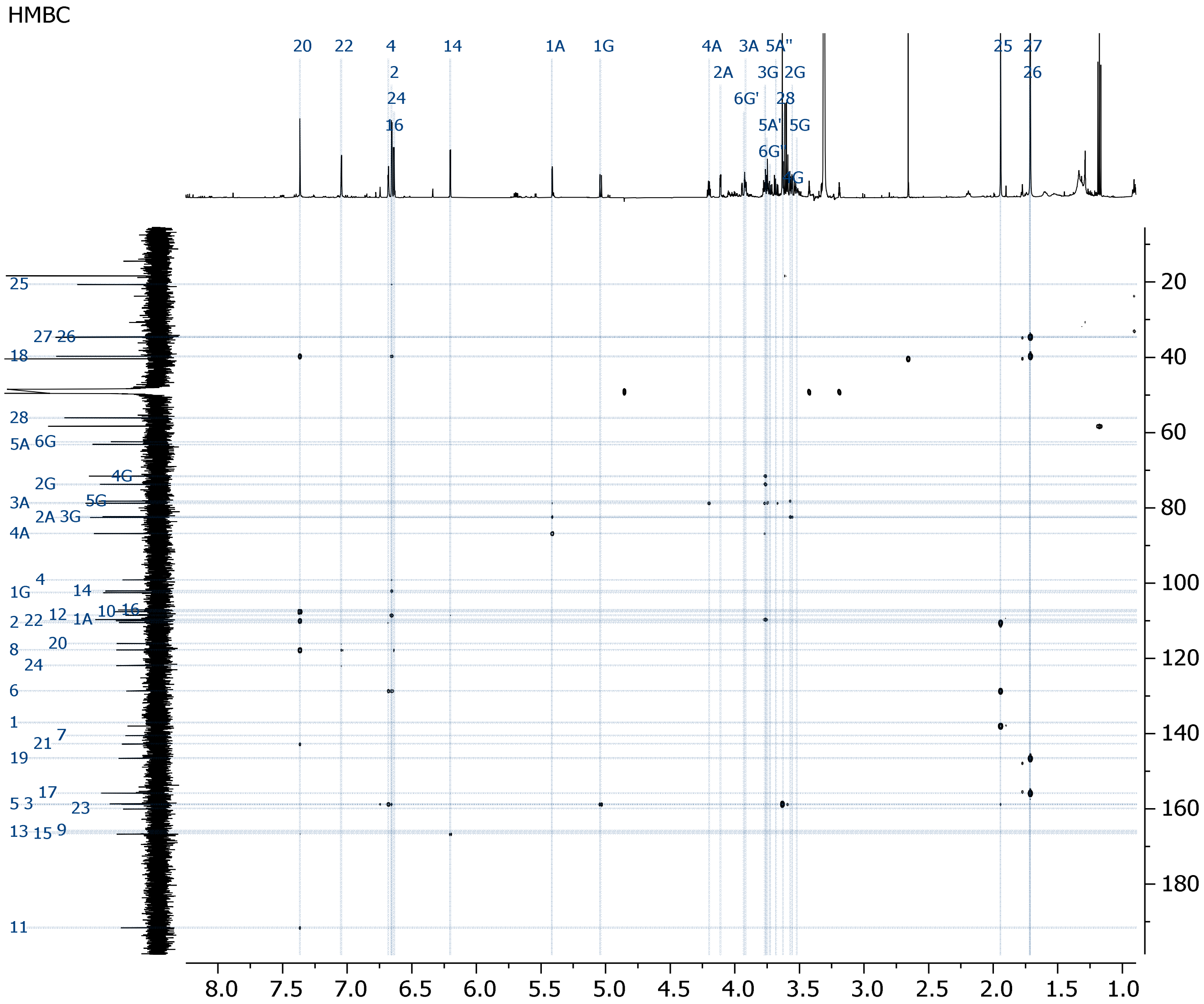

Figure S47 ^{1}H-^{13}C HMBC spectrum (CD_3OD, 298K) of fasamycin glycoside **4**

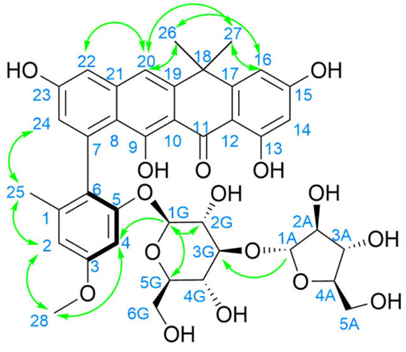

4

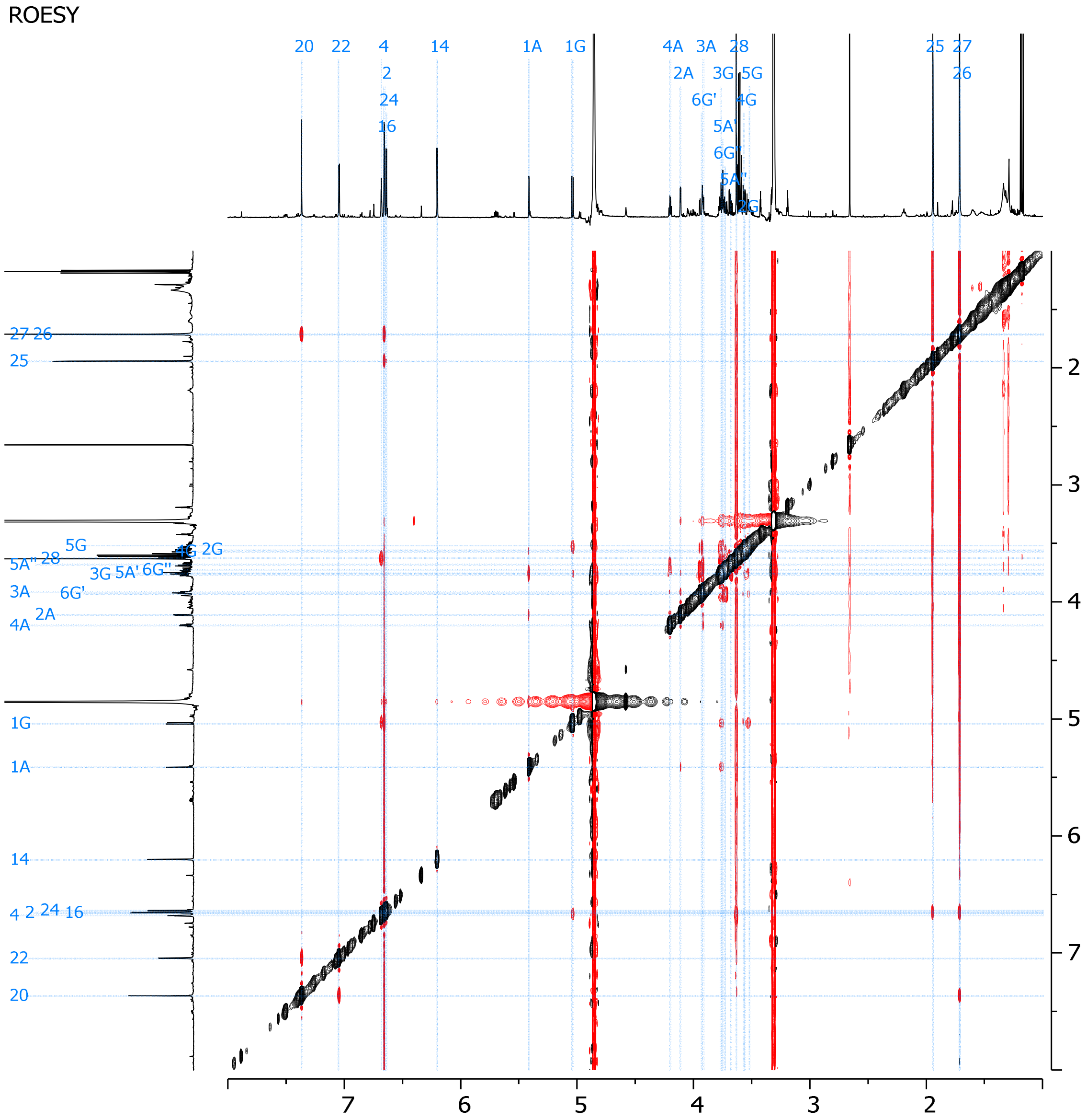

Figure S48 ^{1}H-^{1}H ROESY spectrum (CD_3OD, 298K) of fasamycin glycoside **4**

Table S6 Resonances assignment in ^{1}H and ^{13}C NMR spectra of compound 4

No	δ_H	δ_C	COSY	HSQC-EDITED	HMBC	ROESY
**1**	-	137.95	-	-	-	-
**2**	6.66	110.5	-	2	4	28
**3**	-	158.7	-	-	-	-
**4**	6.68	99.1	-	4	3, 5	1G, 28
**5**	-	158.8	-	-	-	-
**6**	-	128.6	-	-	25	-
**7**	-	140.5	-	-	-	-
**8**	-	117.8	-	-	-	-
**9**	-	165.7	-	-	-	-
**10**	-	107.6	-	-	-	-
**11**	-	191.6	-	-	-	-
**12**	-	108.5	-	-	-	-
**13**	-	166.1	-	-	-	-
**14**	6.2	102.1	16	14	12, 15, 16	-
**15**	-	166.7	-	-	-	-
**16**	6.66	107.1	14	16	15,18	26, 27
**17**	-	155.8	-	-	-	-
**18**	-	39.7	-	-	-	-
**19**	-	146.6	-	-	-	-
**20**	7.37	116.1	-	20	8, 9, 10, 11, 18, 22	22, 26, 27
**21**	-	142.8	-	-	-	-
**22**	7.05	110	24	22	8	20
**23**	-	160	-	-	-	-
**24**	6.64	121.9	22	24	8, 25	25
**25**	1.94	20.7	-	-	1, 2, 6	24
**26**	1.71	34.6	-	26	17, 18,19, 27	16,20
**27**	1.71	34.7	-	27	17, 18,19, 26	16,20
**28**	3.63	56.1	-	28	3	2, 4
**1A**	5.41	109.7	2A	1A	3A, 4A, 3G	3G
**2A**	4.11	82.6	1A, 3A	2A	3A	-
**3A**	3.92	78.8	2A, 4A	3A	1A, 2A, 5A	-
**4A**	4.2	86.8	3A, 5''A, 5'A	4A	3A	-
**5'A**	3.75	63.2	4A	5A	-	-
**5''A**	3.68	63.2	4A	5A	-	-
**1G**	5.04	102.5	2G	1G	5	4, 5G, 2G
**2G**	3.56	73.8	1G, 3G	2G	-	1G
**3G**	3.77	82.4	2G	3G	-	1A
**4G**	3.57	71.6	5G	-	-	-
**5G**	3.52	78.2	4G, 6''G	5G	-	1G
**6'G**	3.93	62.5	-	-	-	-
**6''G**	3.73	62.5	5G	6G	-	-

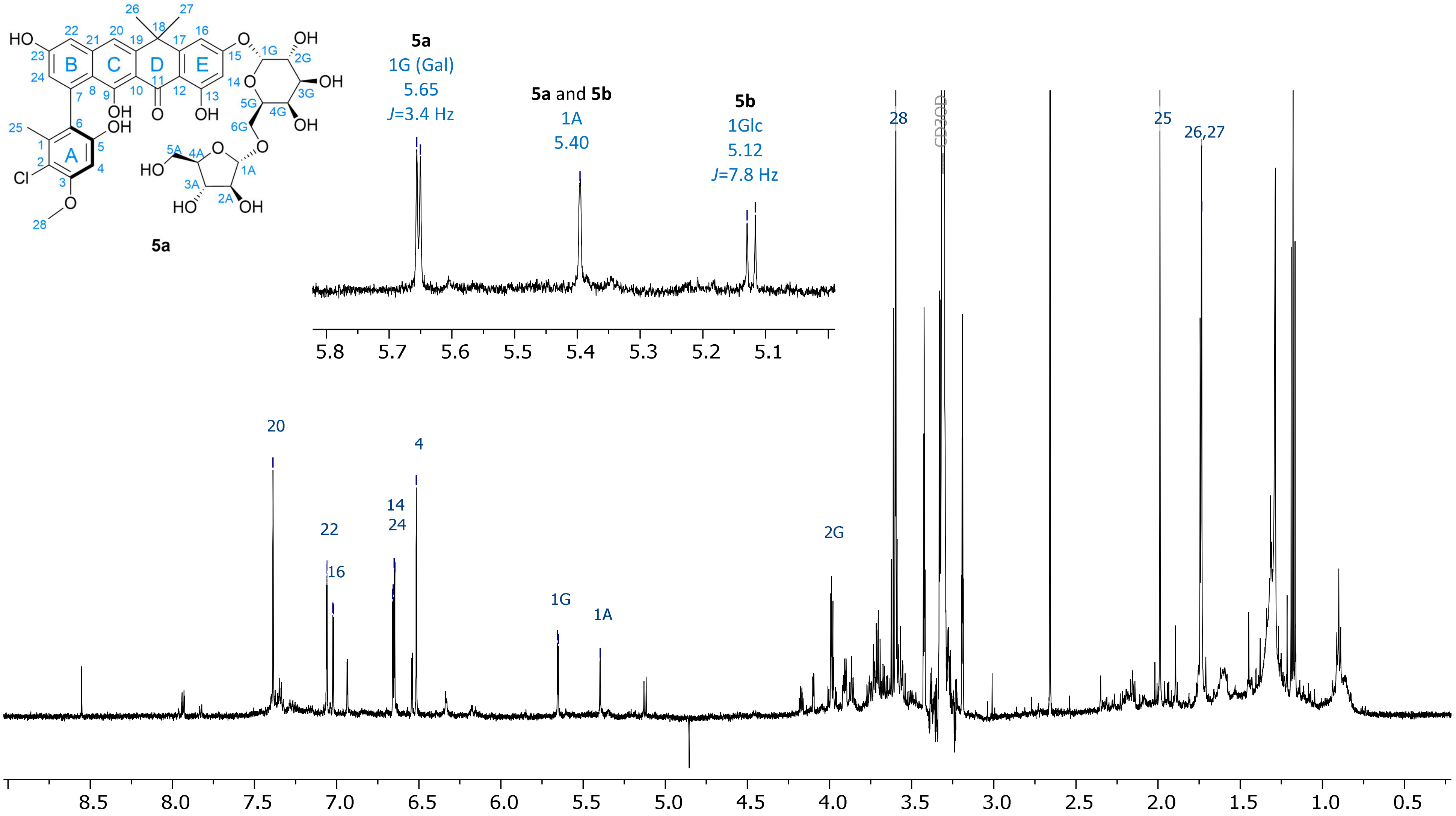

Figure S49 ^{1}H NMR spectrum (CD_3OD, 600 MHz, 298K) of fasamycin glycoside **5a**

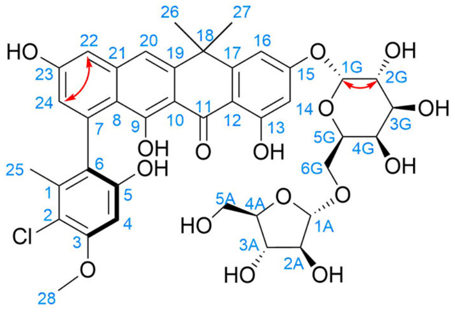

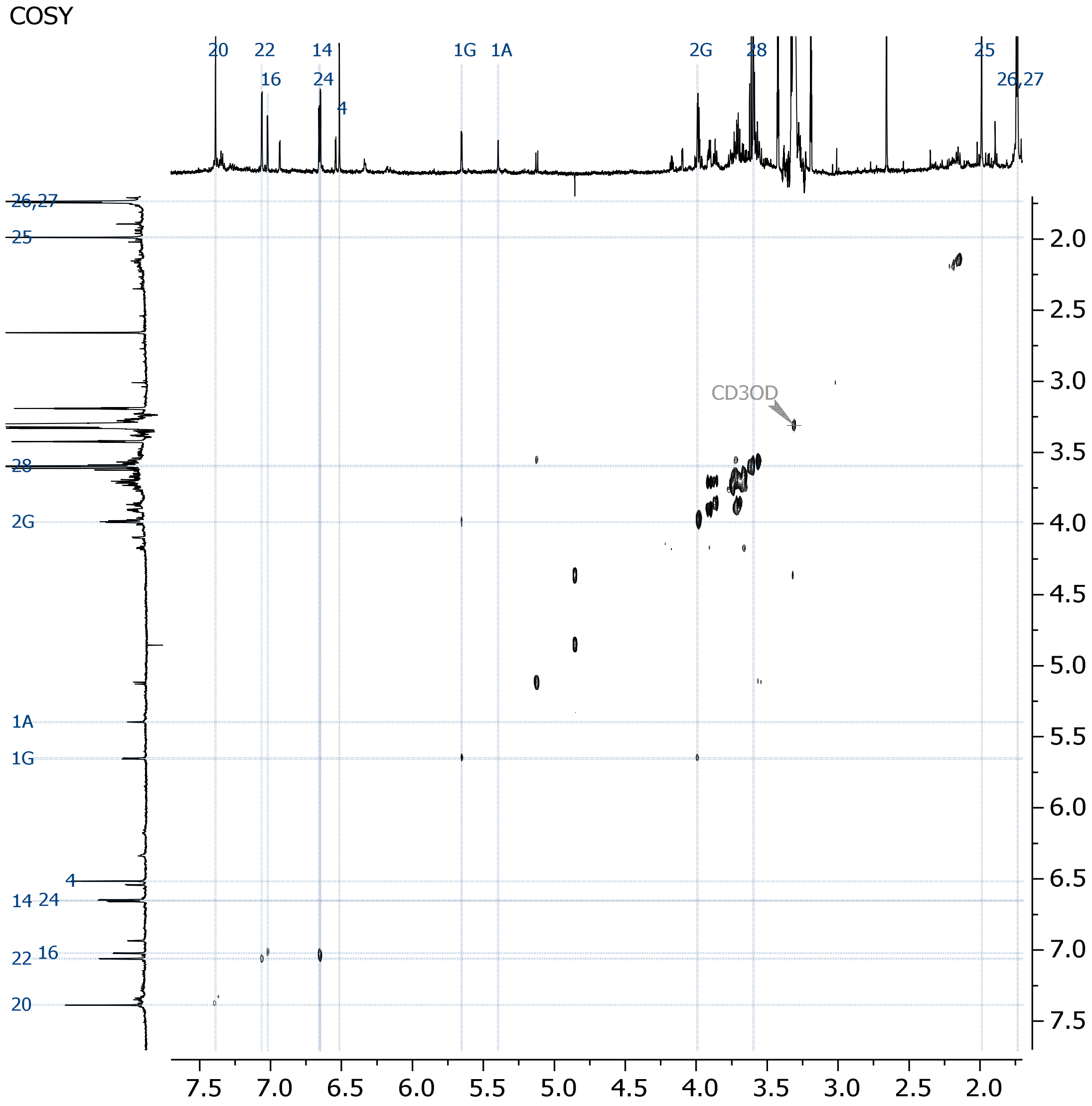

Figure S50 ^{1}H-^{1}H COSY spectrum (CD_3OD, 298K) of fasamycin glycoside **5a**

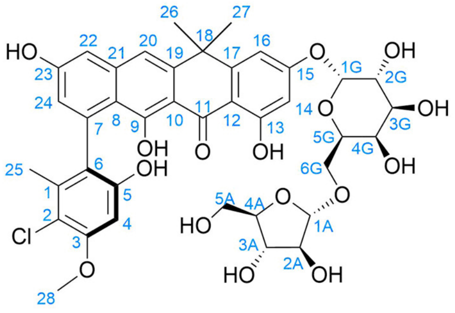

5a

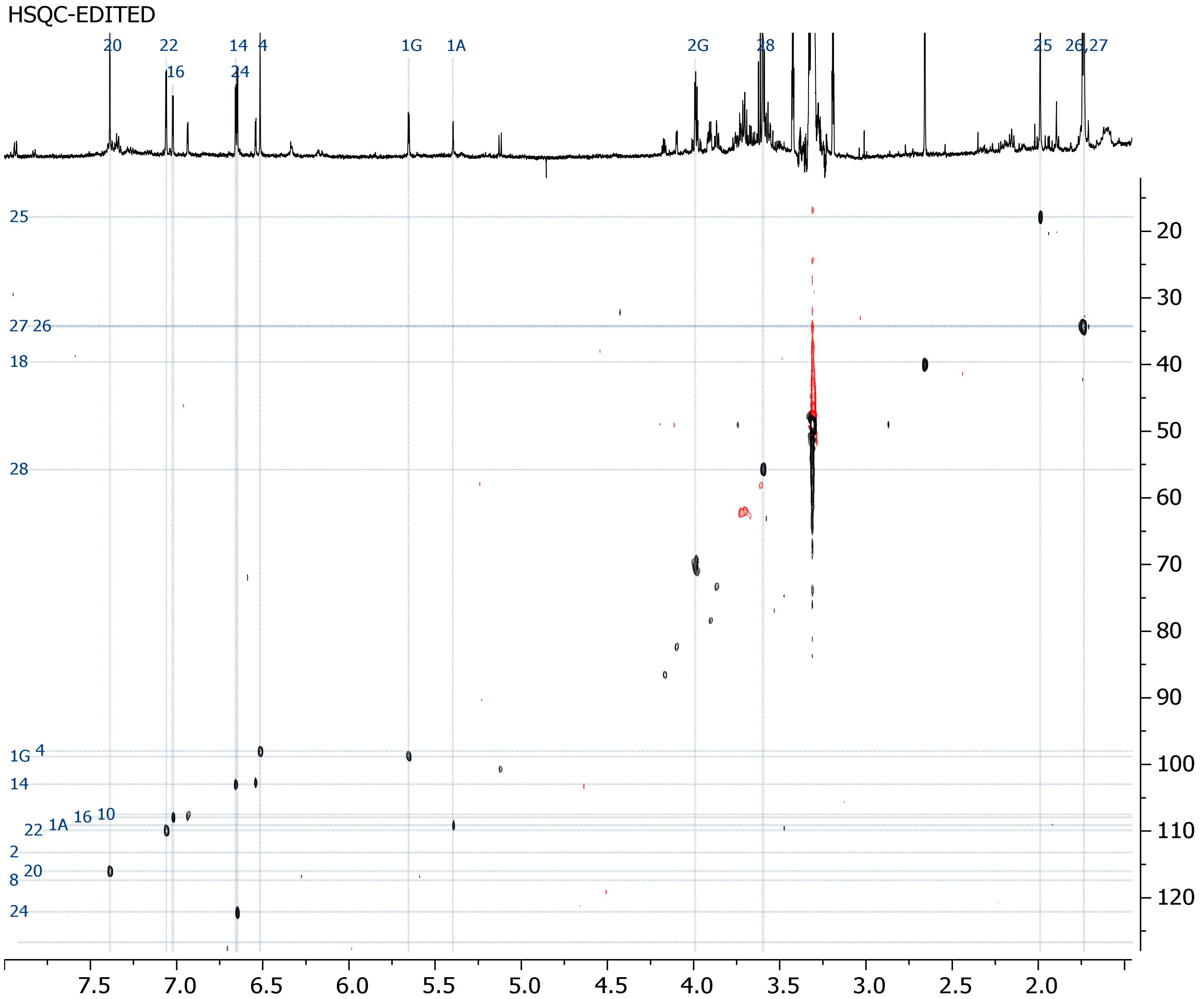

Figure S51 ^{1}H-^{13}C HSQC-edited spectrum (CD_3OD, 298K) of fasamycin glycoside **5a**

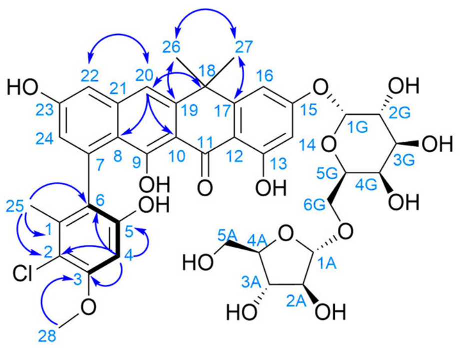

5a

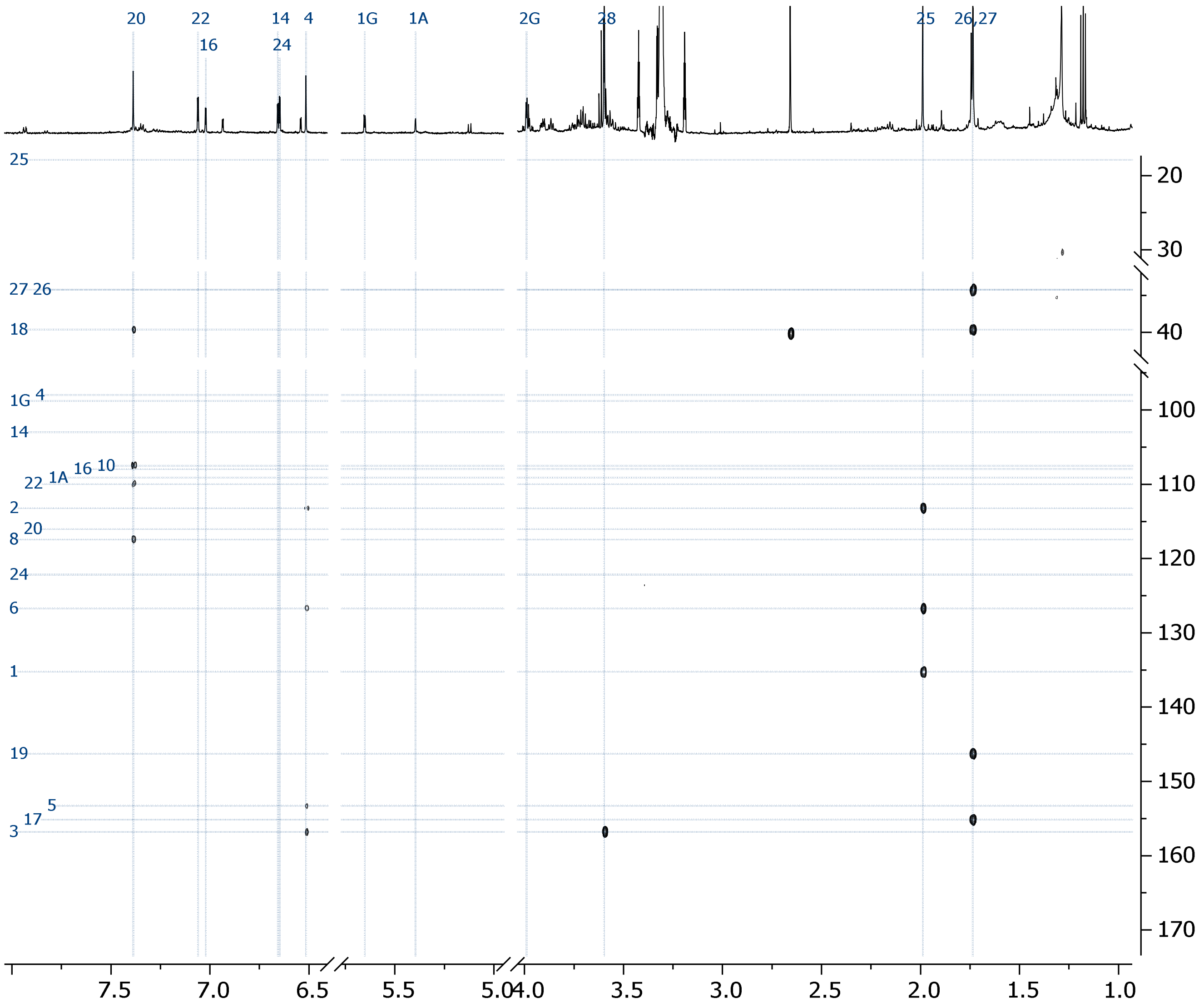

Figure S52 ^{1}H-^{13}C HMBC spectrum (CD_3OD, 298K) of fasamycin glycoside **5a**

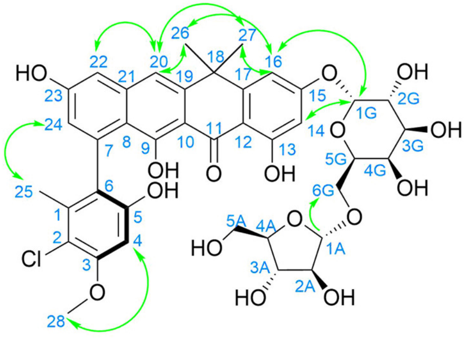

5a

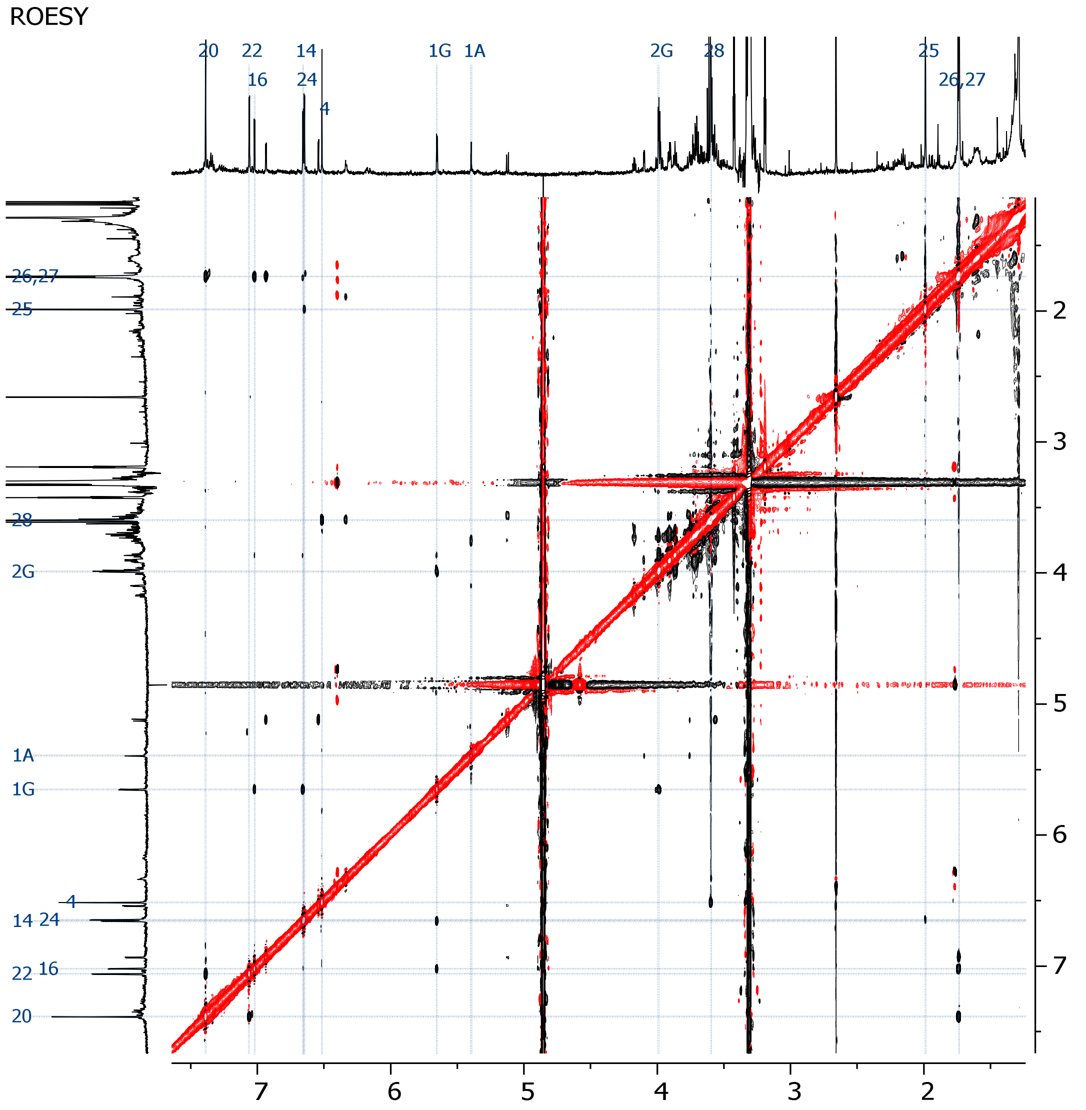

Figure S53 ^{1}H-^{1}H ROESY spectrum (CD_3OD, 298K) of fasamycin glycoside **5a**

Table S7 Resonances assignment in ^{1}H and ^{13}C NMR spectra of compound 5a

No	δ_H*	δ_C**	HSQC-EDITED	HMBC	ROESY
**1**	-	135.2	-	-	-
**2**	-	113.2	-	-	-
**3**	-	156.8	-	-	-
**4**	6.52	98	4	2, 3, 5, 6	28
**5**	-	153.3	-	-	-
**6**	-	126.7	-	-	-
**8**	-	117.4	-	-	-
**9**	-		-	-	-
**10**	-	107.5	-	-	-
**11**	-		-	-	-
**12**	-		-	-	-
**13**	-		-	-	-
**14**	6.66	103	14	-	1G
**15**	-	0	-	-	-
**16**	7.02	107.9	16	-	1G, 26, 27
**17**	-	155.2	-	-	-
**18**	-	39.6	-	-	-
**19**	-	146.3	-	-	-
**20**	7.39	116	20	8, 10, 18, 22	22, 26, 27
**21**	-		-	-	-
**22**	7.06	109.9	22	-	20
**23**	-		-	-	-
**24**	6.65	122.1	24	-	25
**25**	1.99	17.9	25	1, 2,6	24
**26**	1.74	34.3	26	17, 18,19,27	16, 20
**27**	1.74	34.3	27	17, 18,19,26	16, 20
**28**	3.6	55.8	28	3	4
**1A**	5.4	109.1	1A	-	6G
**1G**	5.65	98.8	1G	-	14, 16

*Positions of H2A – H5A and H2G – H6G were not determined due to overlaps and low signal intensities.

**Chemical shift values were measured using HSQC and HMBC spectra.

Signals

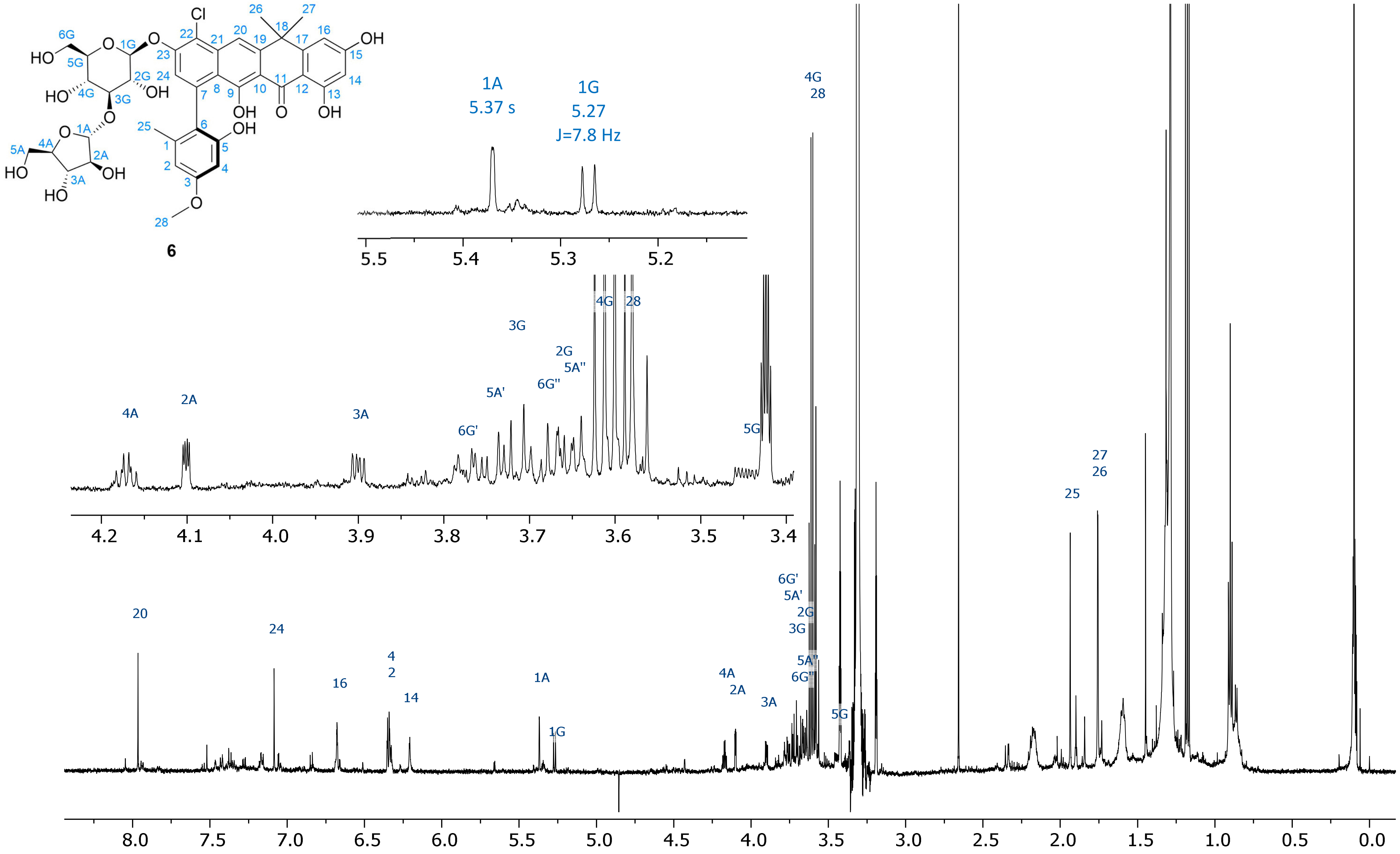

Figure S54 ^{1}H NMR spectrum (CD_3OD, 600 MHz, 298K) of fasamycin glycoside **6**

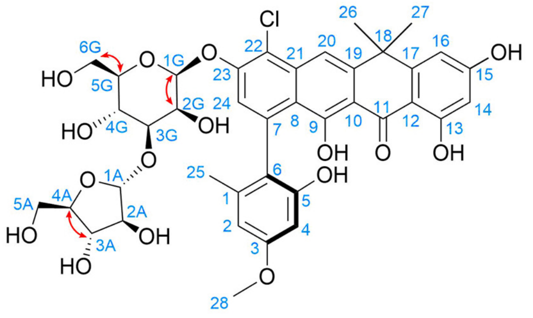

6

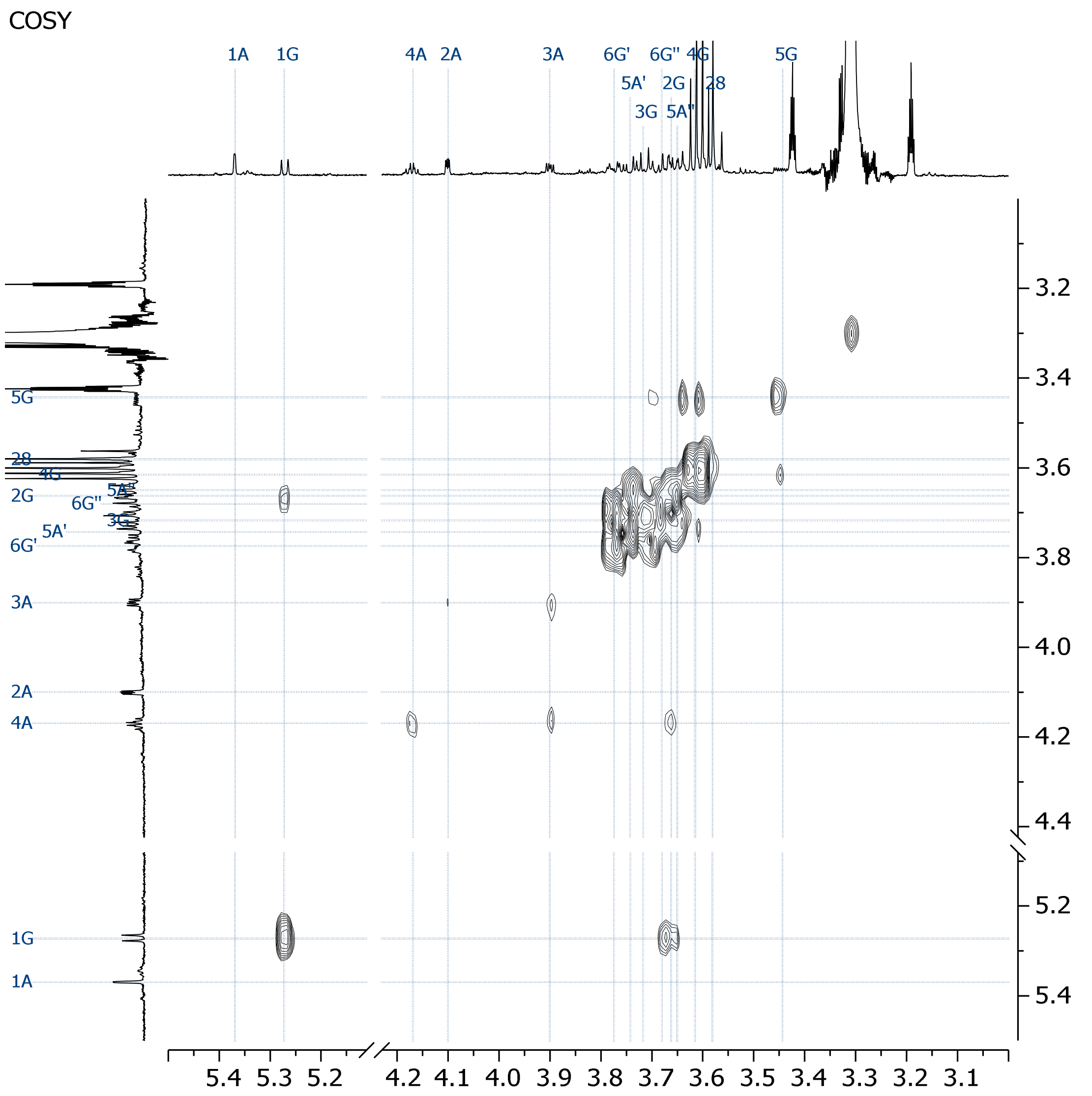

Figure S55 ^1H-^1H COSY spectrum (CD_3OD, 298K) of fasamycin glycoside **6** zoomed in carbohydrate signals region

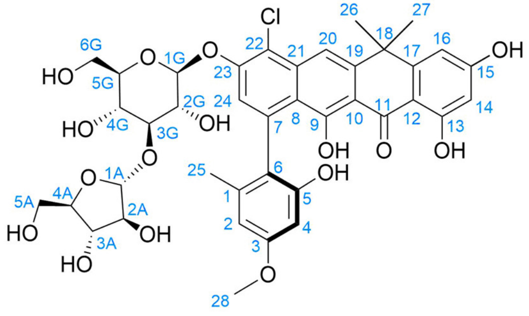

6

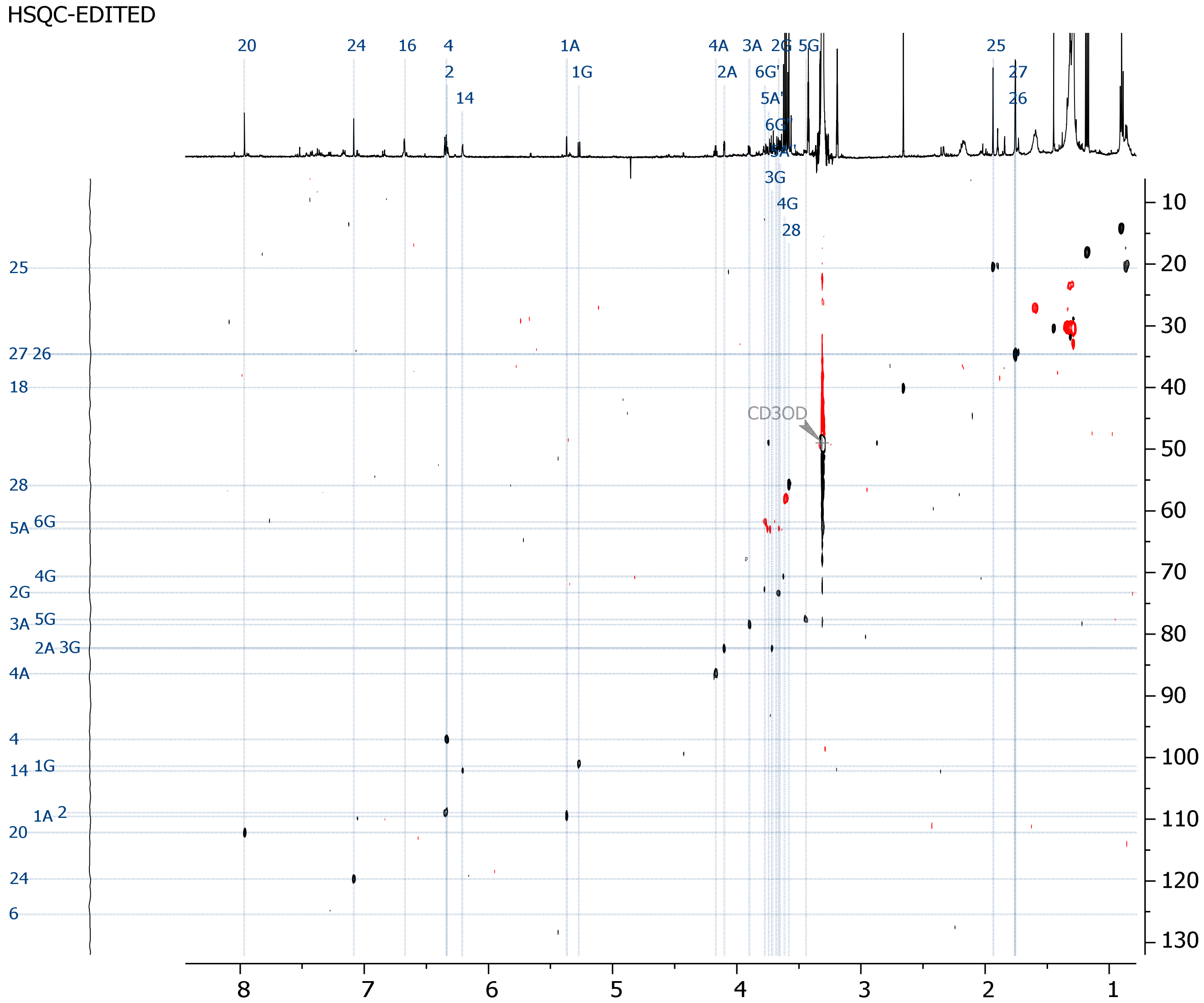

Figure S56 ^{1}H-^{13}C HSQC-edited spectrum (CD_3OD, 298K) of fasamycin glycoside **6**

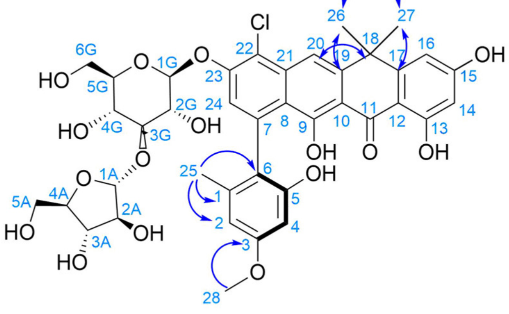

6

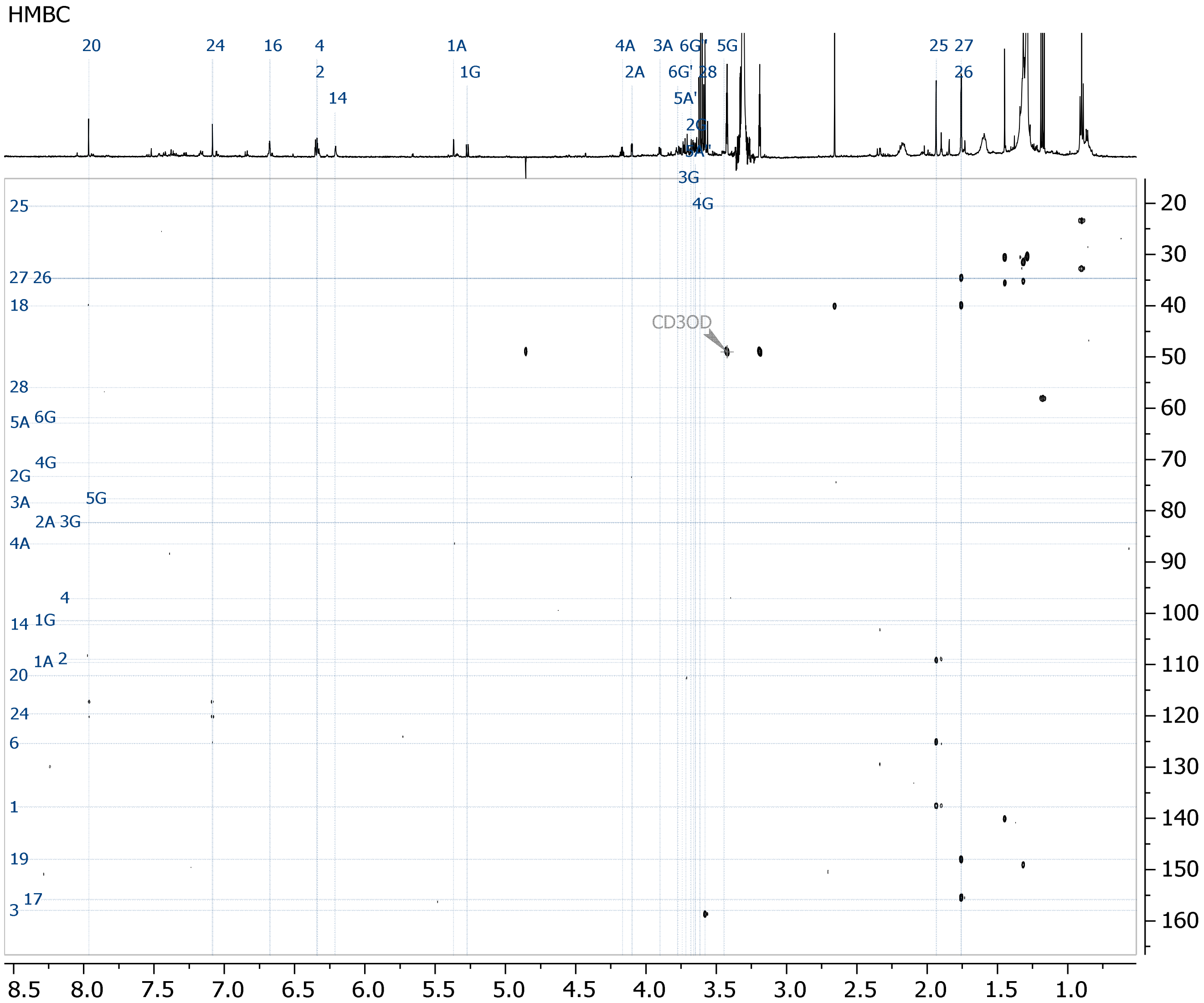

Figure S57 ^{1}H-^{13}C HMBC spectrum (CD_3OD, 298K) of fasamycin glycoside **6**

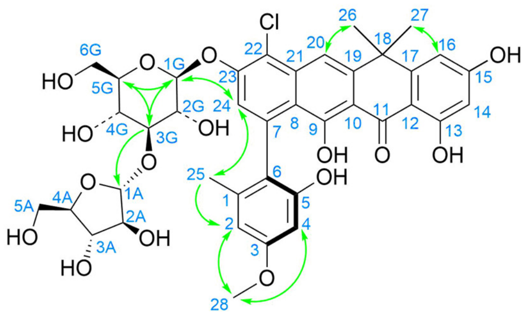

6

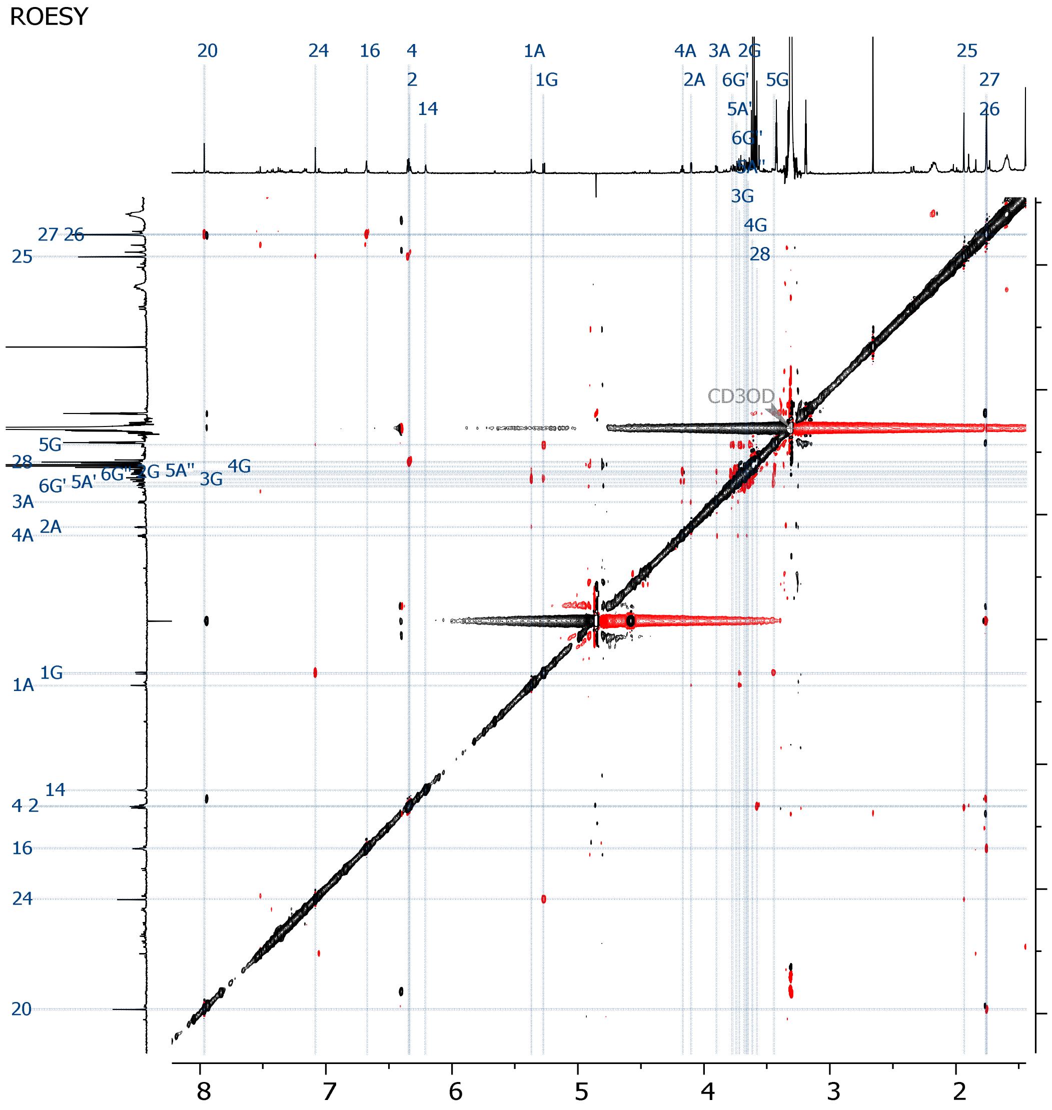

Figure S58 ^{1}H-^{1}H ROESY spectrum (CD_3OD, 298K) of fasamycin glycoside **6**

Table S8 Resonances assignment in ^{1}H and ^{13}C NMR spectra of compound 6

No	δ_H	δ_C*	COSY	HSQC-EDITED	HMBC	ROESY
**1**	-	137.8	-	-	25	-
**2**	6.34	109.0	-	2	-	25, 28
**3**	-	158.0	-	-	28	-
**4**	6.34	97.5	-	4	-	28
**5**	-	n.d.	-	-	-	-
**6**	-	125.4	-	-	25	-
**7**	-	n.d.	-	-	-	-
**8**	-	n.d.	-	-	-	-
**9**	-	n.d.	-	-	-	-
**10**	-	n.d.	-	-	-	-
**11**	-	n.d.	-	-	-	-
**12**	-	n.d.	-	-	-	-
**13**	-	n.d.	-	-	-	-
**14**	6.21	102.2	16	14	-	-
**15**	-	n.d.	-	-	-	-
**16**	6.67	n.d.	14	-	-	27
**17**	-	155.9	-	-	26	-
**18**	-	40.0	-	-	25, 27	-
**19**	-	148.0	-	-	26	-
**20**	7.96	112.2	-	20	-	26
**21**	-	n.d.	-	-	-	-
**22**	-	n.d.	-	-	-	-
**23**	-	n.d.	-	-	-	-
**24**	7.08	119.7	-	24	-	25, 1G
**25**	1.94	20.6	-	25	1, 2, 6, 18	2, 24
**26**	1.76	34.6	-	26	17, 19	20
**27**	1.76	34.6	-	27	18	16
**28**	3.58	55.9	-	28	3	2, 4
**1A**	5.37	109.6	-	1A	-	3G
**2A**	4.10	82.3	-	2A	-	3A
**3A**	3.90	78.4	-	3A	-	2A, 4A
**4A**	4.17	86.5	-	4A	-	3A, 5''A, 5'A
**5'A**	3.74	62.9	-	5A	-	4A
**5''A**	3.65	62.9	-	5A	-	4A
**1G**	5.27	101.4	-	1G	-	24, 5G, 3G
**2G**	3.66	73.3	-	2G	-	-
**3G**	3.72	82.2	-	3G	-	5G, 1G, 1A
**4G**	3.62	70.7	-	4G	-	-
**5G**	3.44	77.6	-	5G	-	1G, 3G, 6''G, 6'G
**6'G**	3.77	61.8	-	6G	-	5G
**6''G**	3.68	61.8	-	6G	-	5G

*Chemical shift values were measured using HSQC and HMBC spectra.

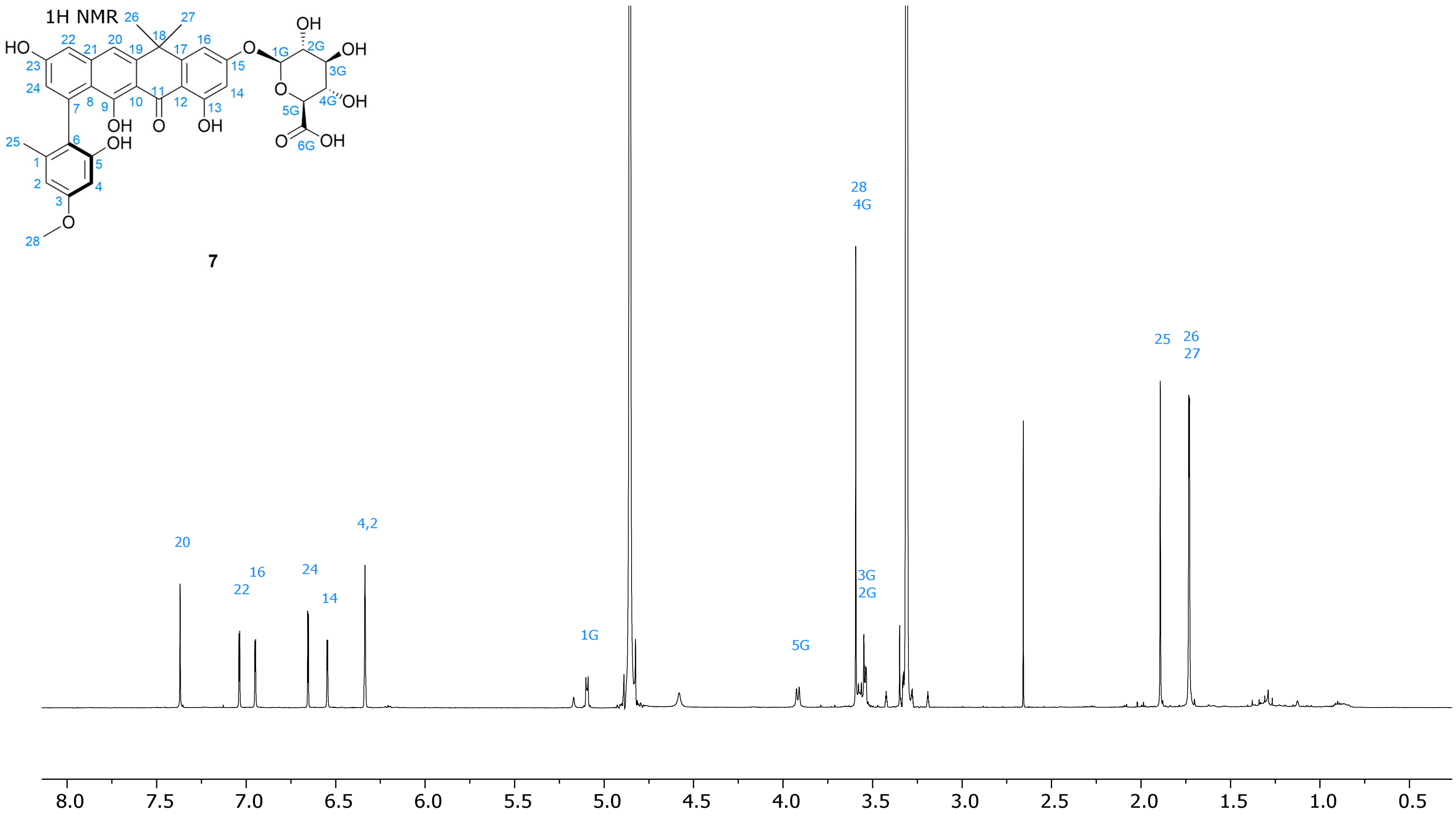

Figure S59 ^{1}H NMR spectrum (CD_3OD, 600 MHz, 298K) of fasamycin glycoside **7**

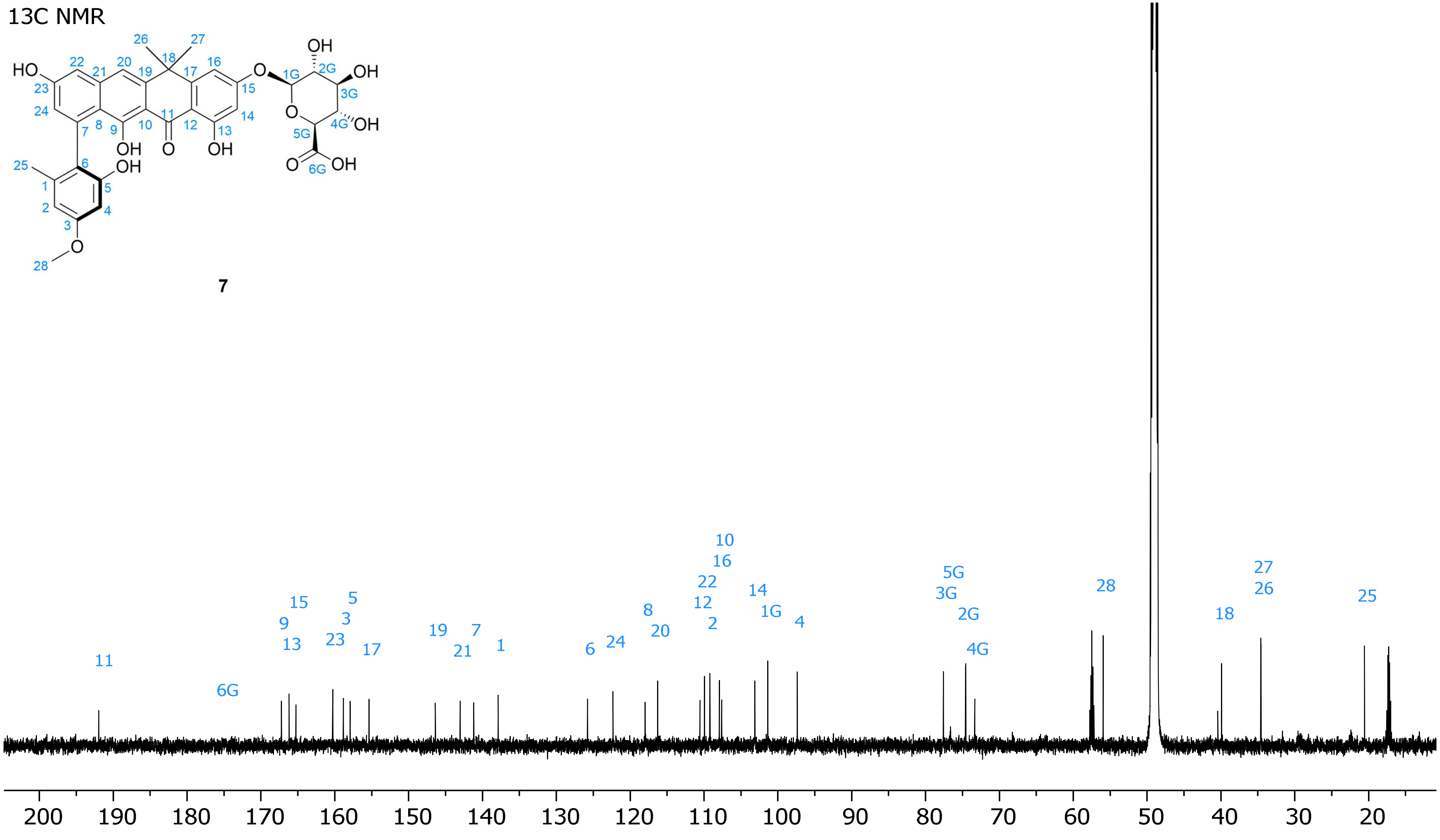

Figure S60 ^{13}C NMR spectrum (CD_3OD, 150 MHz, 298K) of fasamycin glycoside **7**

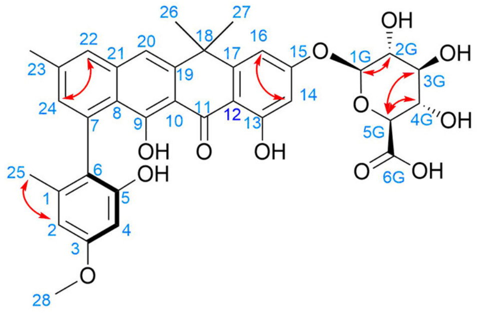

7

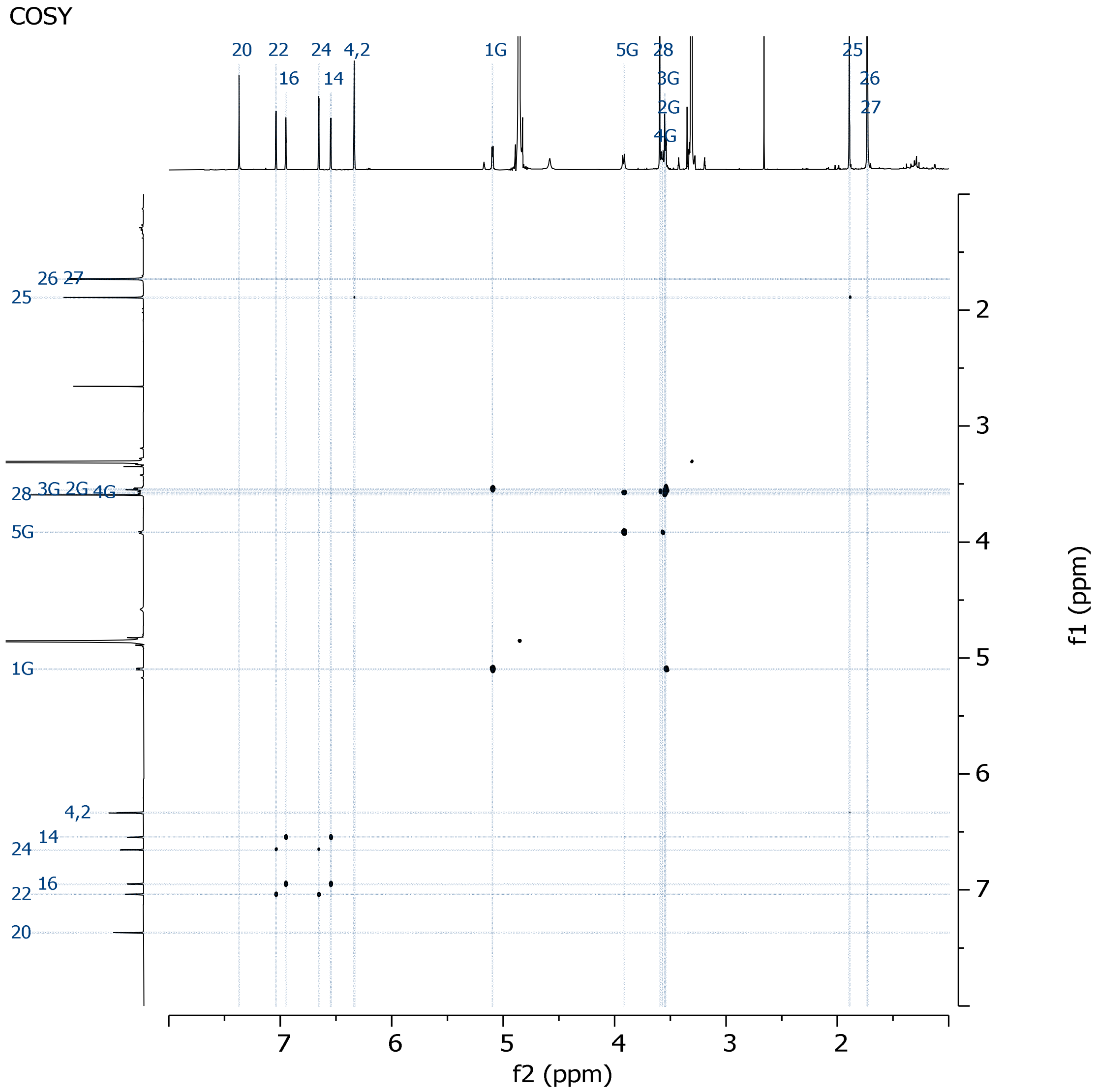

Figure S61 ^1H-^1H COSY spectrum (CD_3OD, 298K) of fasamycin glycoside **7**

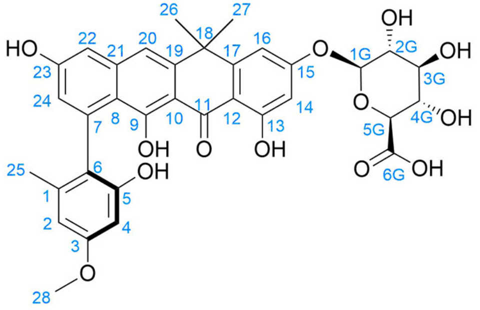

7

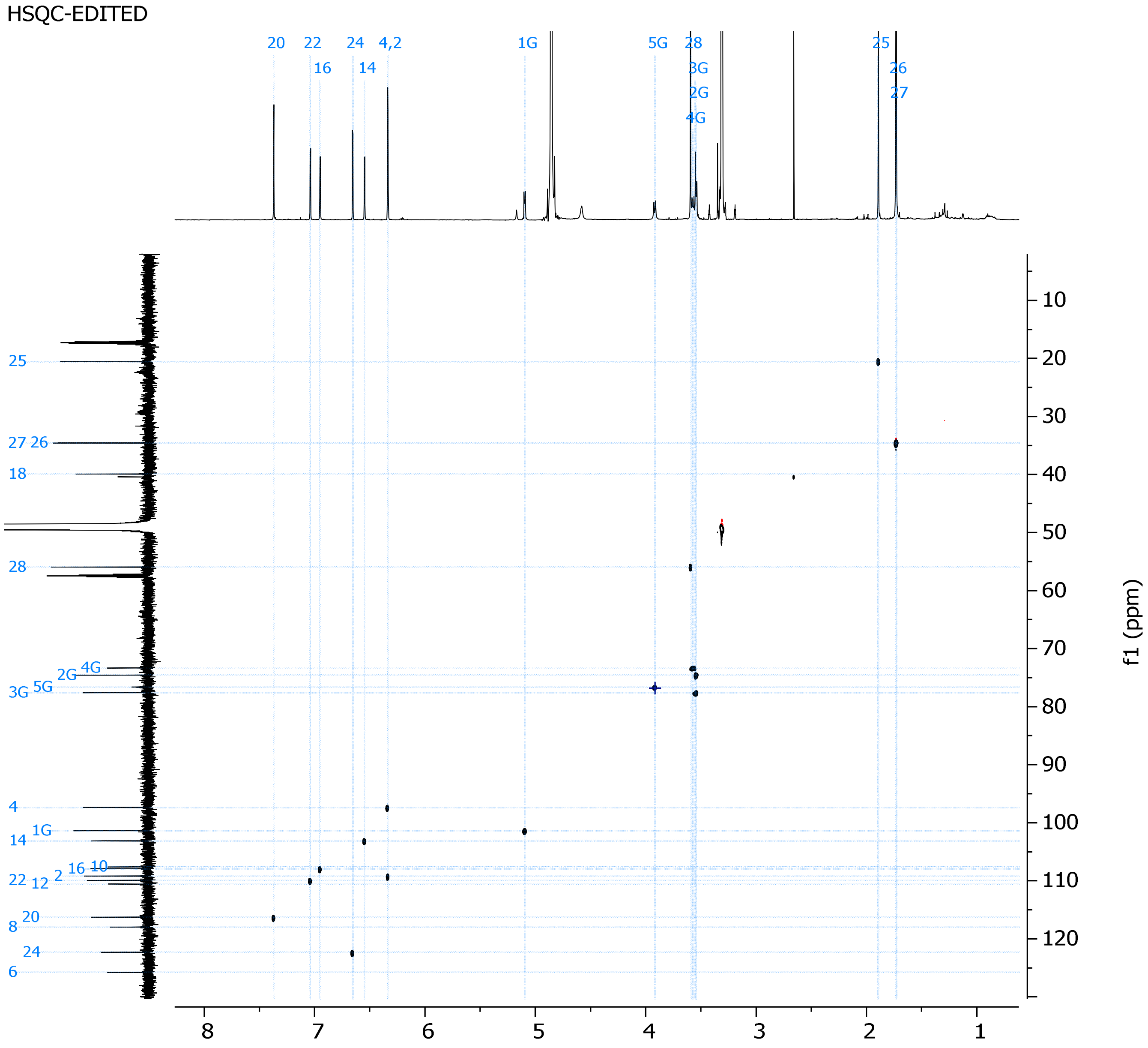

Figure S62 1H-13C HSQC-edited spectrum (CD_3OD, 298K) of fasamycin glycoside **7**

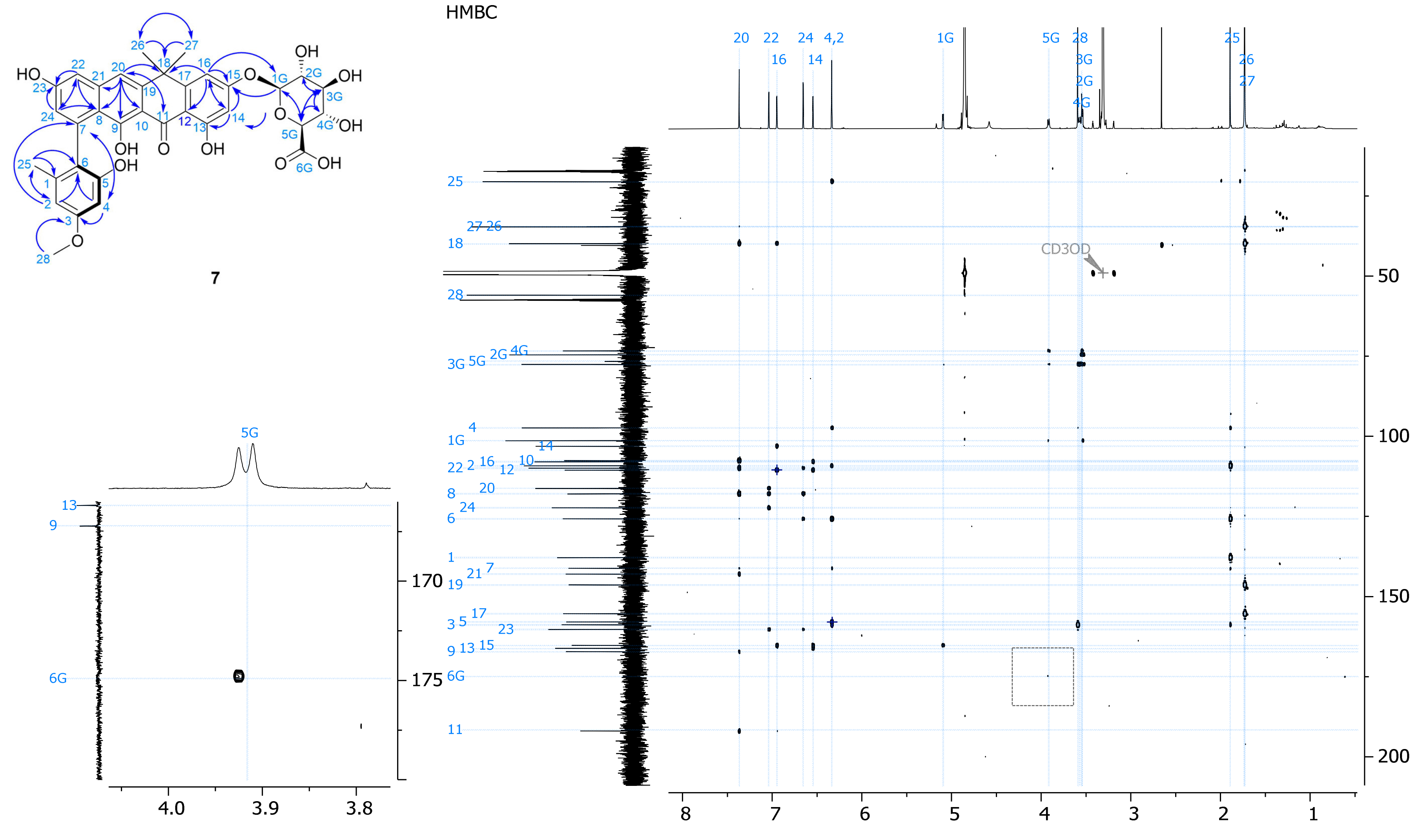

Figure S63 ^{1}H-^{13}C HMBC spectrum (CD_3OD, 298K) of fasamycin glycoside **7**

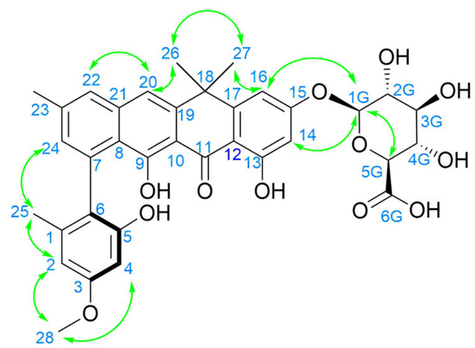

7

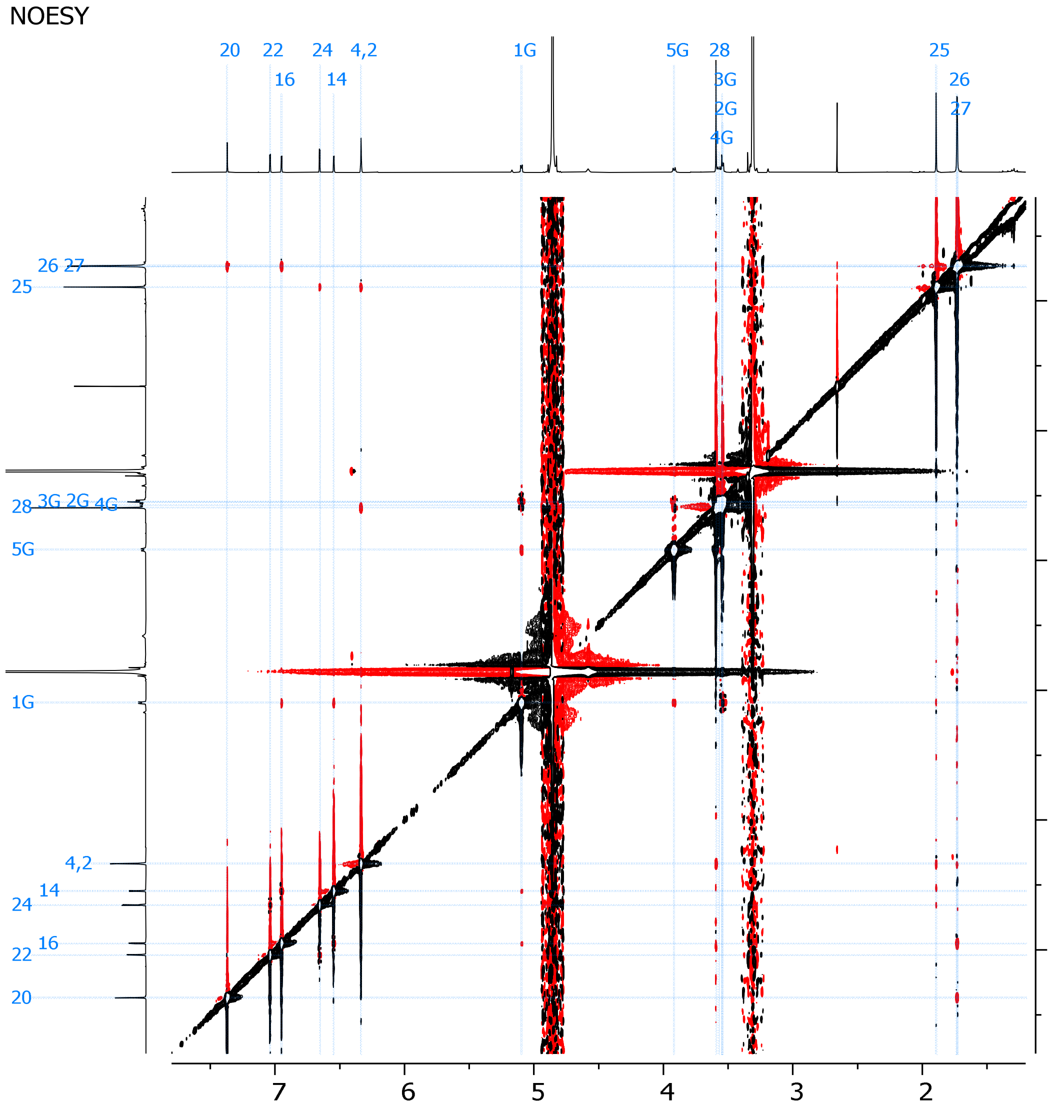

Figure S64 ^{1}H-^{1}H NOESY spectrum (CD_3OD, 298K) of fasamycin glycoside **7**

Table S9 Resonances assignment in ^{1}H and ^{13}C NMR spectra of compound 7

No	δ_H	δ_C	HSQC-EDITED	COSY	HMBC	NOESY
**1**	-	137.9	-	-	-	-
**2**	6.33	109.2	2	25	6, 7, 25	25, 28
**3**	-	158.8	-	-	-	-
**4**	6.33	97.4	4	-	3, 6, 7	25, 28
**5**	-	157.9	-	-	-	-
**6**	-	125.8	-	-	-	-
**7**	-	141.2	-	-	-	-
**8**	-	118	-	-	-	-
**9**	-	167.2	-	-	-	-
**10**	-	107.6	-	-	-	-
**11**	-	191.6	-	-	-	-
**12**	-	110.6	-	-	-	-
**13**	-	166.2	-	-	-	-
**14**	6.55	103.1	-	16	12, 13, 15, 16	1G
**15**	-	165.3	-	-	-	-
**16**	6.95	107.9	16	14	-	1G, 26, 27
**17**	-	155.4	-	-	-	-
**18**	-	39.9	-	-	-	-
**19**	-	146.4	-	-	-	-
**20**	7.37	116.3	-	-	8, 9, 10, 11, 18, 21, 22	22, 26, 27
**21**	-	143	-	-	-	-
**22**	7.04	109.9	22	24	8, 20, 23, 24	20
**23**	-	160.3	-	-	-	-
**24**	6.65	122.3	24	22	6, 8, 22, 23	25
**25**	1.89	20.6	25	2	1, 2, 3, 4, 6	2,24
**26**	1.73	34.6	26	-	17, 18,19, 27	16,20
**27**	1.73	34.6	27	-	17, 18,19, 26	16,20
**28**	3.59	56	28	-	3,4	2,4
**1G**	5.1	101.4	1G	2G	15	5G, 14, 16
**2G**	3.54	74.6	2G	1G	3G	-
**3G**	3.55	77.6	3G	5G	4G, 2G, 5G	-
**4G**	3.57	73.3	4G	6G	3G, 5G	-
**5G**	3.92	76.6	5G	4G, 3G	4G, 3G, 1G	1G
**6G***	-	174.9	6G	-	-	-

*Chemical shift δ_C was determined using HMBC spectra.

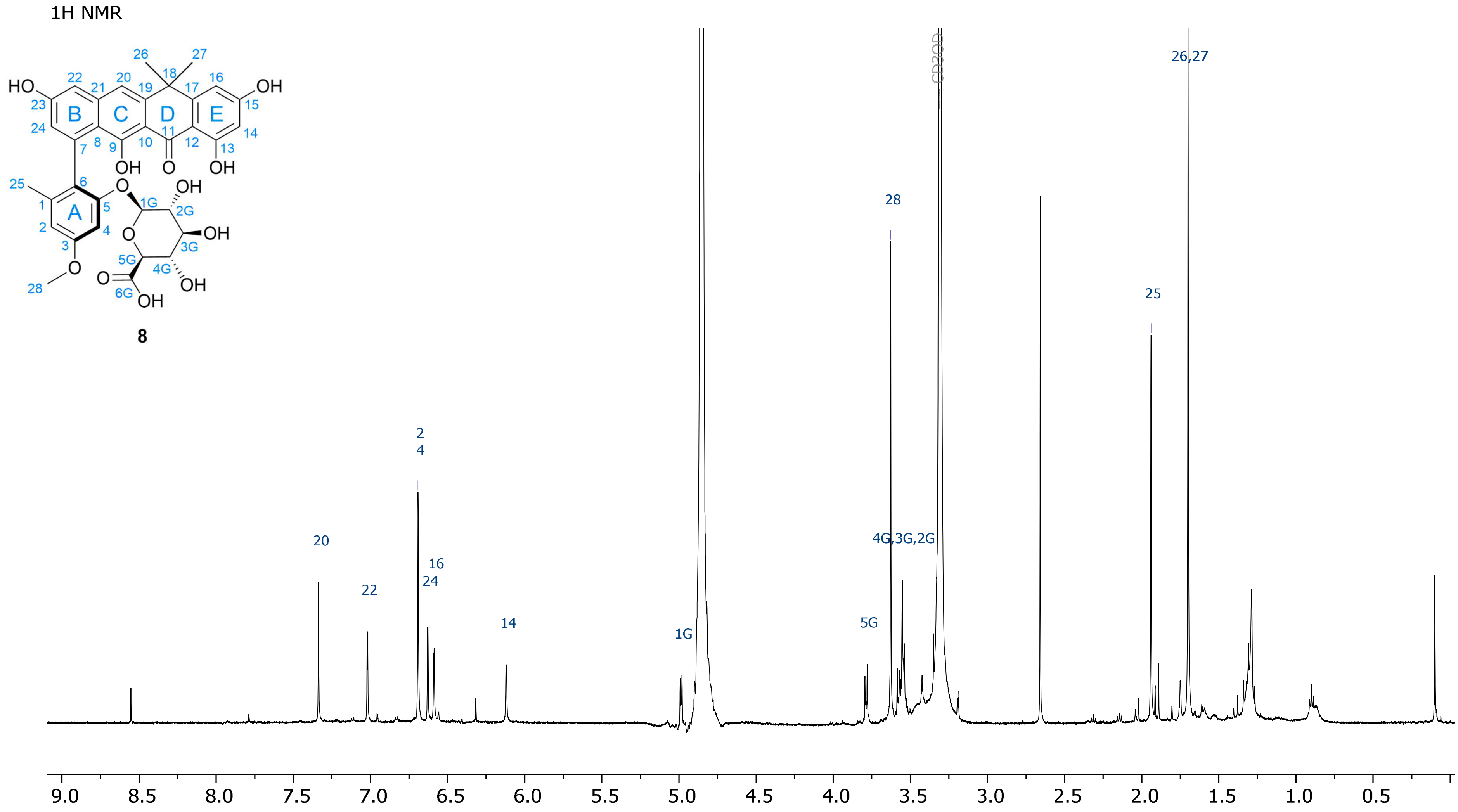

Figure S65 ^{1}H NMR spectrum (CD_3OD, 600 MHz, 298K) of fasamycin glycoside **8**

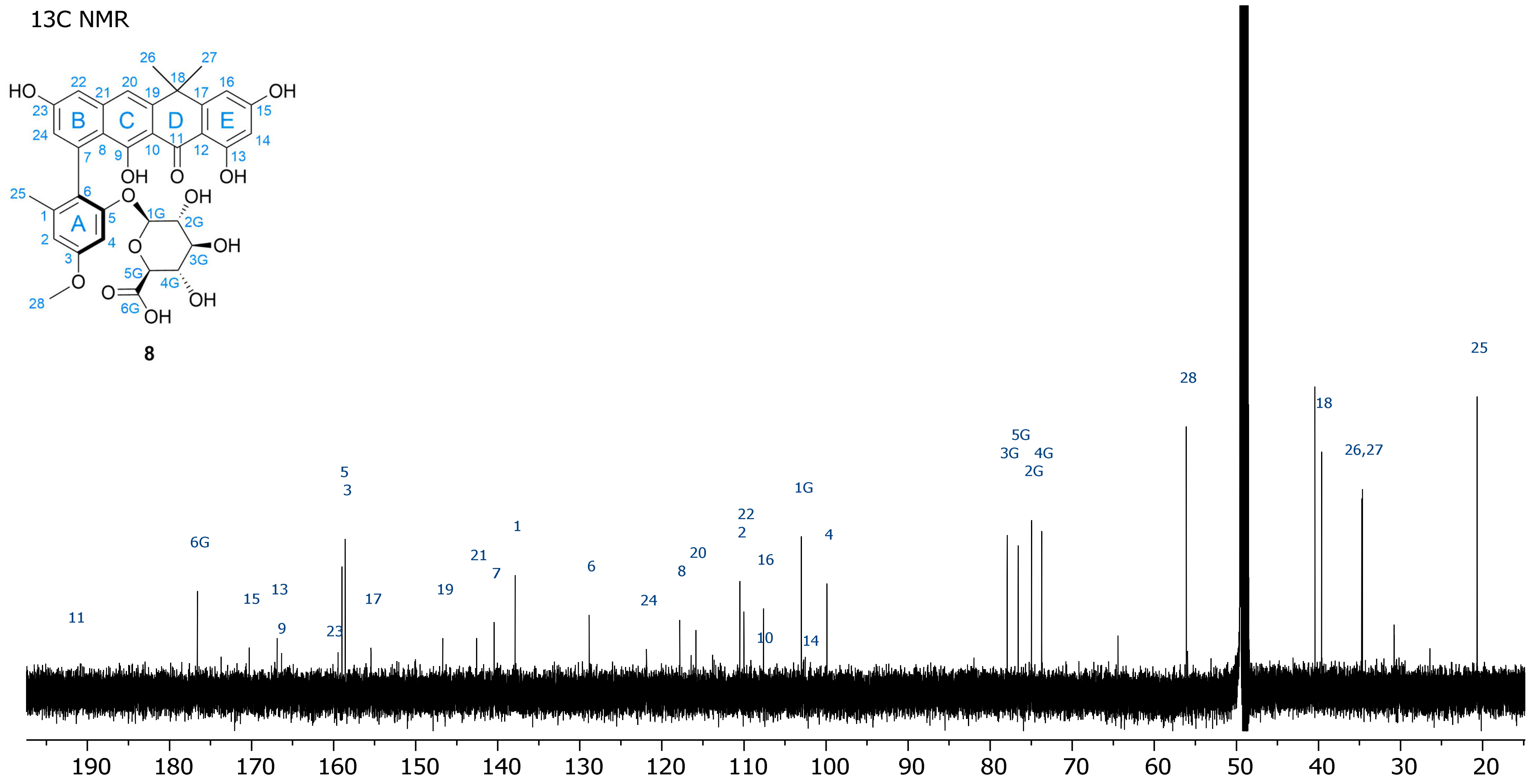

Figure S66 ^{13}C NMR spectrum (CD_3OD, 150 MHz, 298K) of fasamycin glycoside **8**

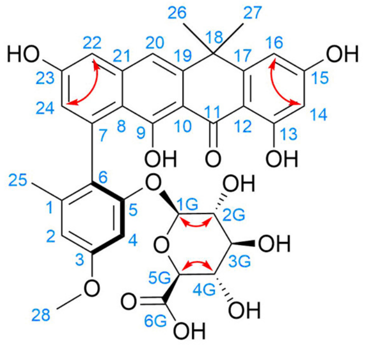

8

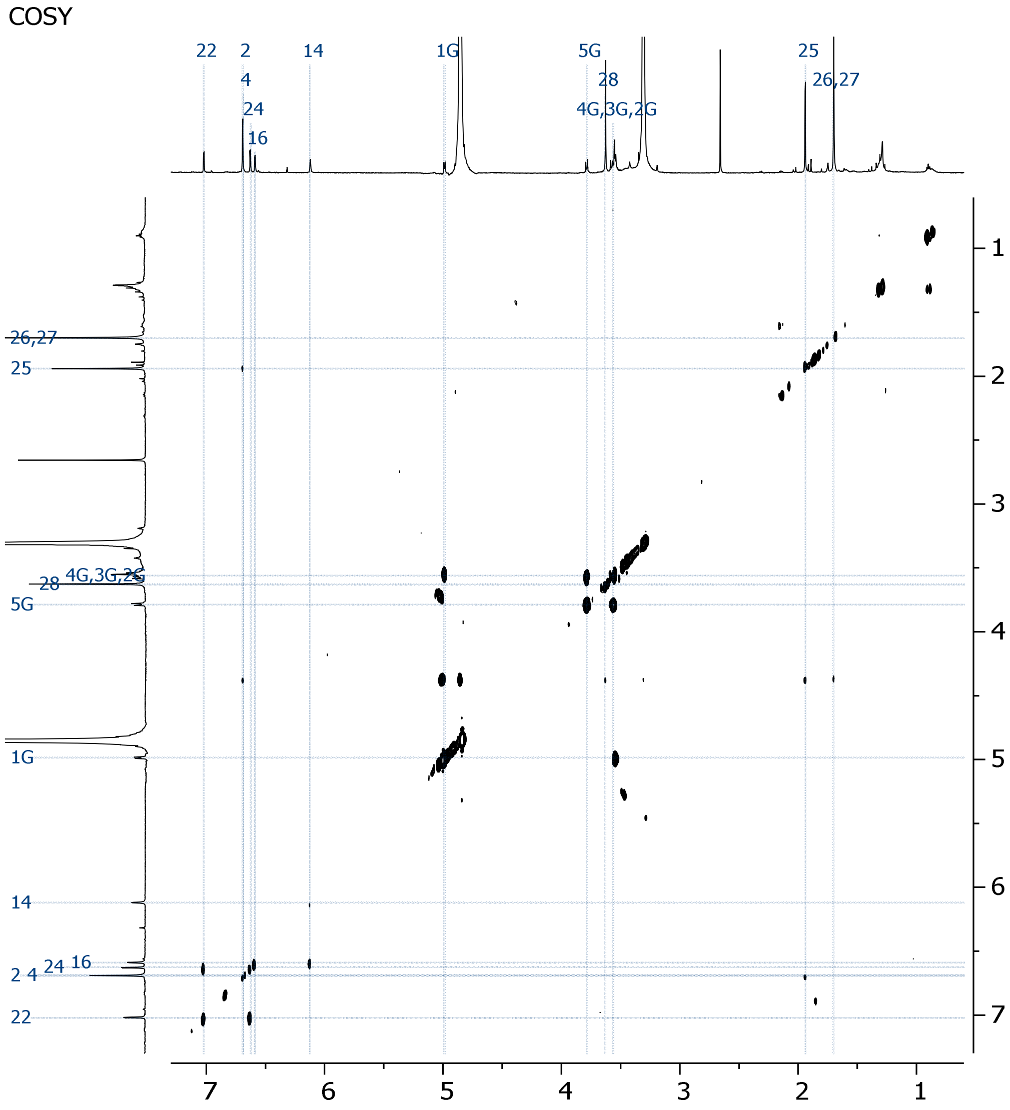

Figure S67 ^1H-^1H COSY spectrum (CD_3OD, 298K) of fasamycin glycoside **8**

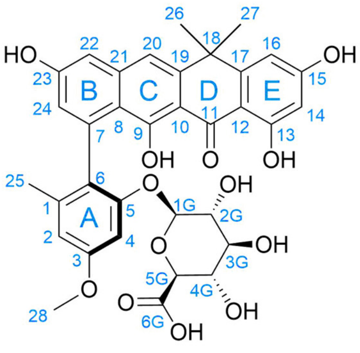

8

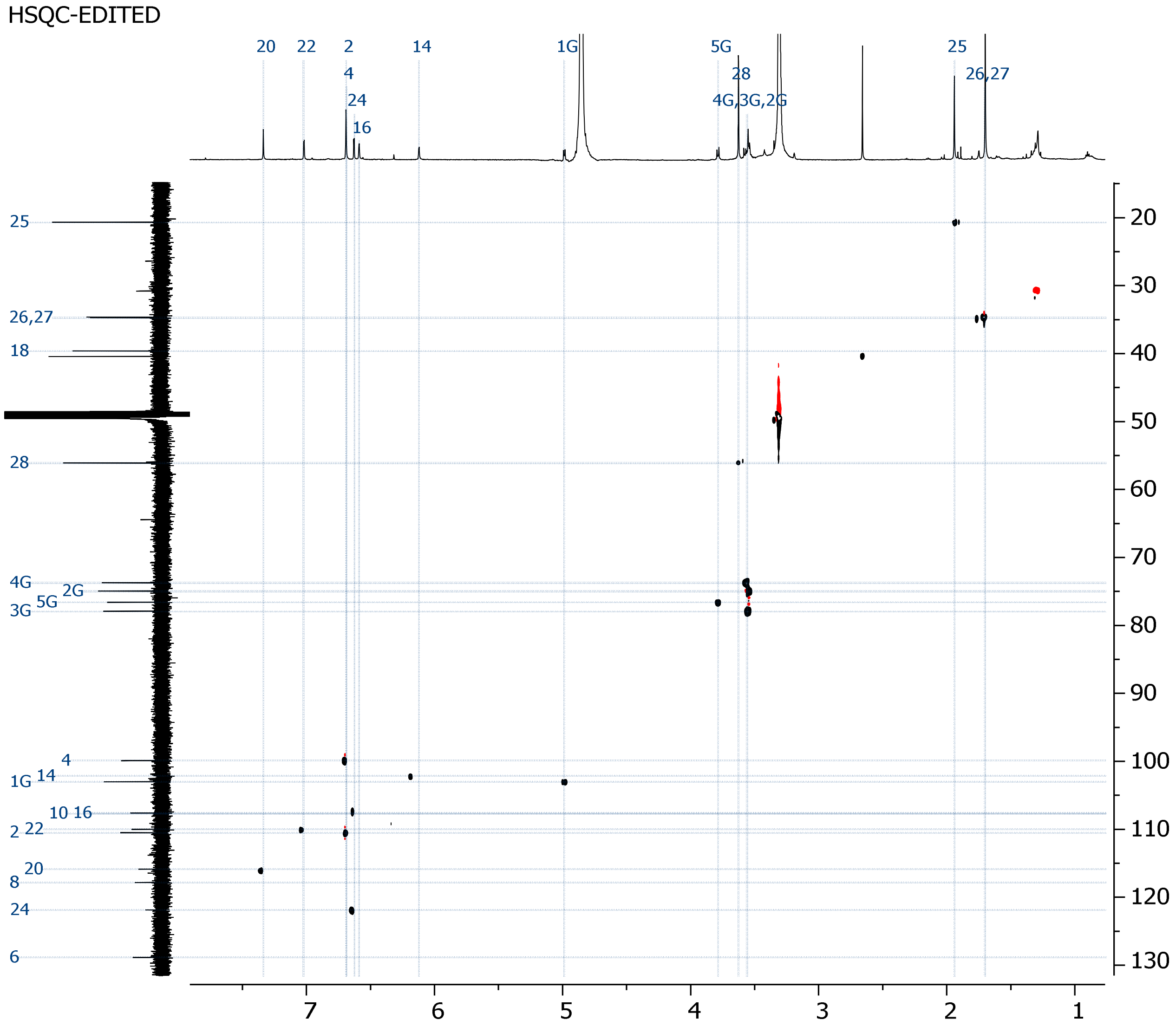

Figure S68 ^{1}H-^{13}C HSQC-edited spectrum (CD_3OD, 298K) of fasamycin glycoside **8**

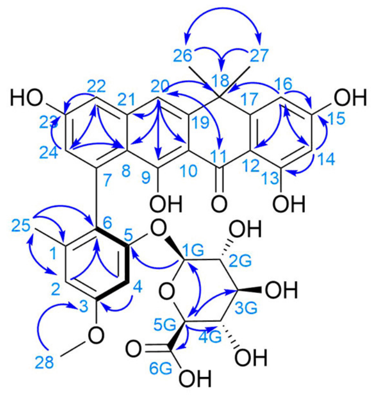

8

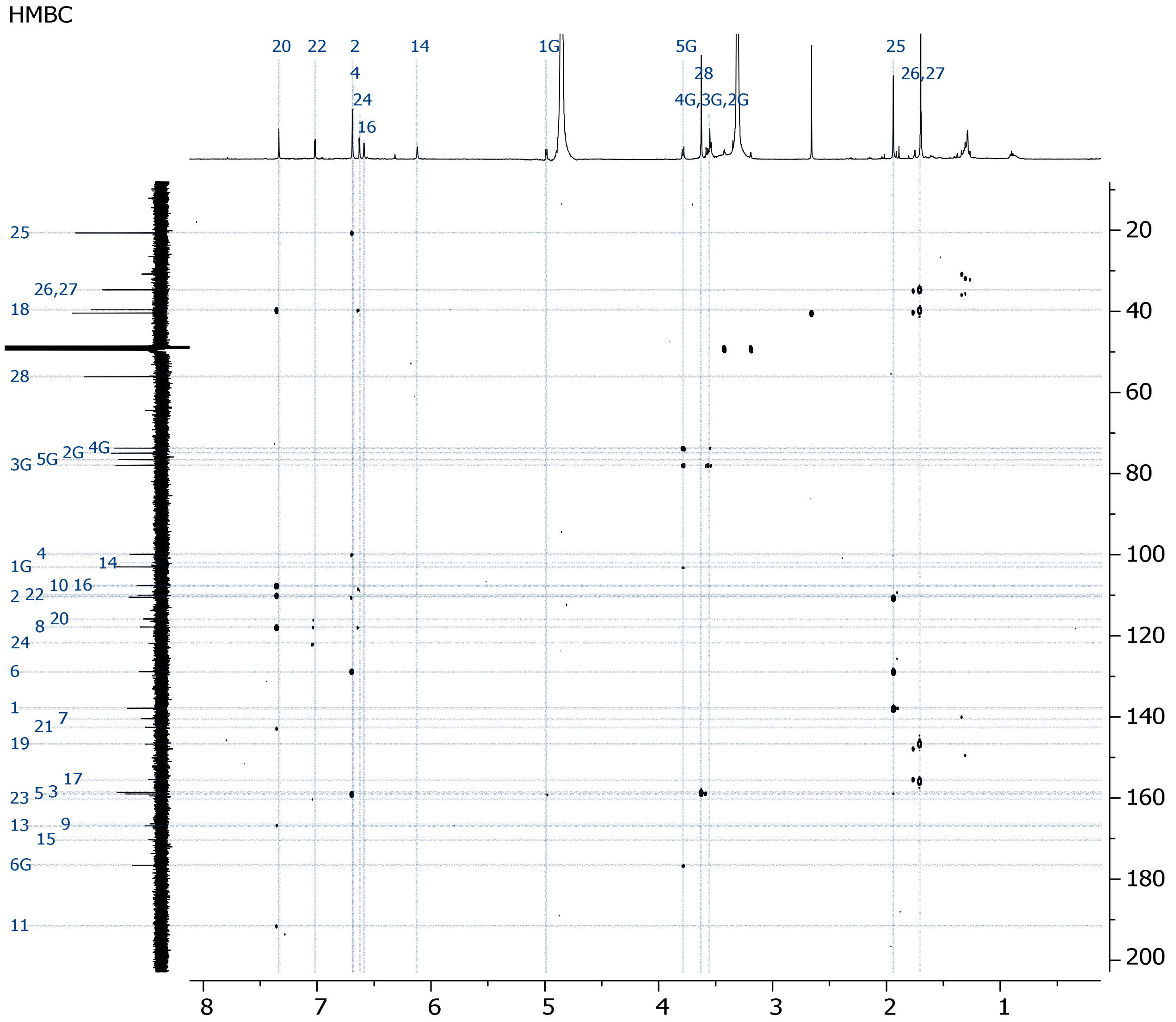

Figure S69 ^{1}H-^{13}C HMBC spectrum (CD_3OD, 298K) of fasamycin glycoside **8**

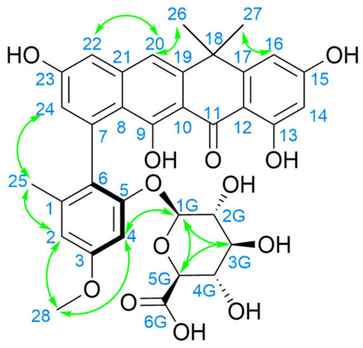

8

ROESY

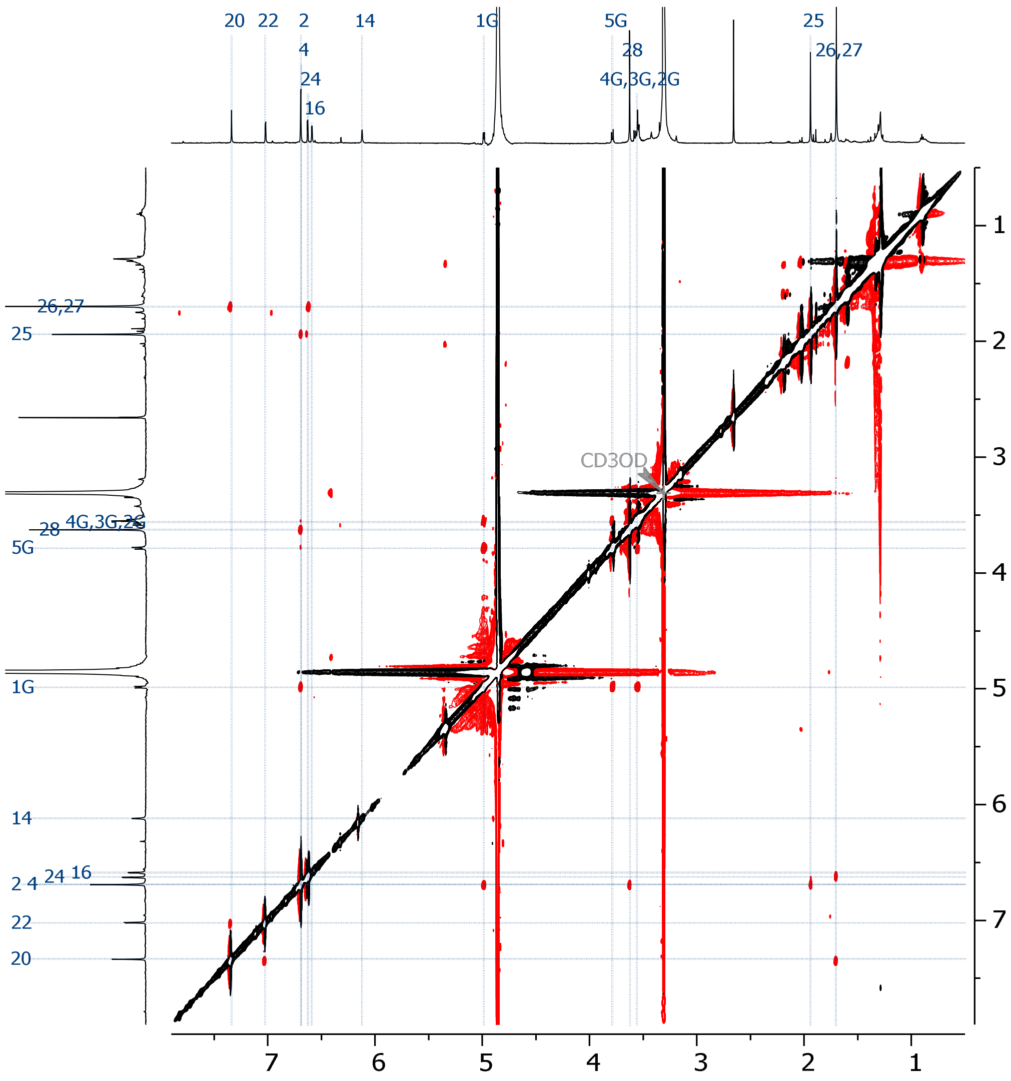

Figure S70 ^{1}H-^{1}H ROESY spectrum (CD_3OD, 298K) of fasamycin glycoside **8**

Table S10 Resonances assignment in ^{1}H and ^{13}C NMR spectra of compound 8

No	δ_H	δ_C	HSQC-EDITED	HMBC	COSY	ROESY
**1**	-	137.9	-	-	-	-
**2**	6.69	110.5	-	3, 4, 6, 25	-	25, 28
**3**	-	158.6	-	-	-	-
**4**	6.69	99.9	4	2, 3, 5, 6	-	1G, 28
**5**	-	159	-	-	-	-
**6**	-	128.9	-	-	-	-
**7**	-	140.5	-	-	-	-
**8**	-	117.8	-	-	-	-
**9**	-	166.6	-	-	-	-
**10**	-	107.7	-	-	-	-
**11**	-	191.6	-	-	-	-
**12**	n.d	n.d	-	-	-	-
**13**	-	166.9	-	-	-	-
**14**	6.12	102.1	14	-	16	-
**15**	-	170.3	-	-	-	-
**16**	6.59	107.6	16	18	14	27
**17**	-	155.4	-	-	-	-
**18**	-	39.6	-	-	-	-
**19**	-	146.7	-	-	-	-
**20**	7.34	115.9	20	8, 9, 10, 11, 18, 21, 22	-	22, 26
**21**	-	142.6	-	-	-	-
**22**	7.02	110	22	8, 20, 24	24	20
**23**	-	160.1	-	-	-	-
**24**	6.62	121.9	24	8, 22	22	25
**25**	1.94	20.7	-	1, 2, 6	-	2, 24
**26**	1.7	34.7	26	17, 19, 27	-	20
**27**	1.7	34.7	27	17, 19, 26	-	16
**28**	3.63	56.1	28	3, 5	-	2, 4
**1G**	4.99	103	1G	5	2G	4, 3G, 5G
**2G**	3.56	74.9	2G	-	2G	-
**3G**	3.56	77.9	3G	4G	-	1G, 5G
**4G**	3.56	73.7	4G	3G	5G	-
**5G**	3.79	76.6	5G	1G, 4G, 6G	4G	3G, 1G
**6G**	-	176.6	-	-	-	-

7. Bioactivity of glycosylated fasamycin congeners

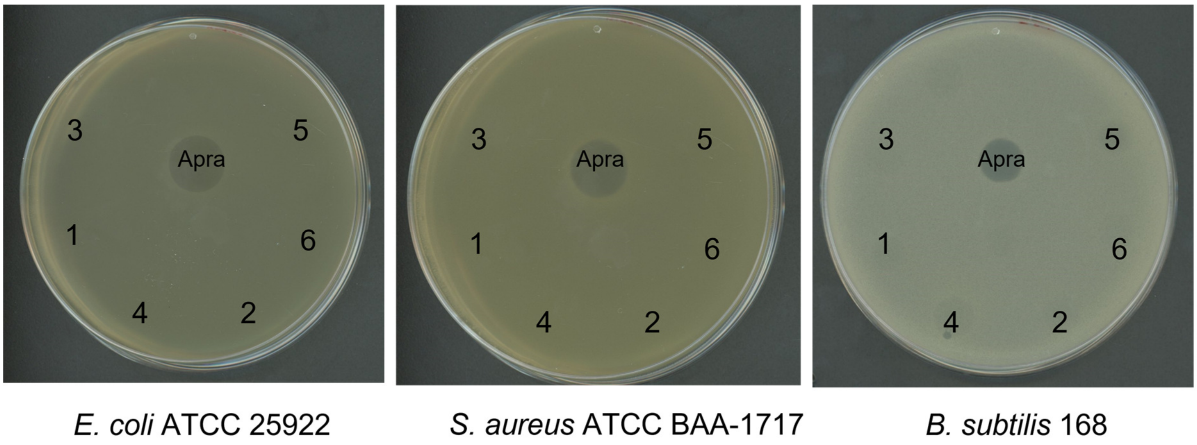

Figure S71 Representative examples of spot on lawn bioassay plates to determine bioactivity of samples 1-6. E. coli ATCC 25922 and S. aureus ATCC BA-1717 were grown to confluence in soft nutrient agar and B. subtilis 168 was grown in LB agar medium containing no salt. Samples 1 (420 µg/mL), 2 (440 µg/mL), 3 (120 µg/mL), 4 (400 µg/mL), 5 (250 µg/mL), 6 (300 µg/mL) and apramycin (150 µg/mL) in methanol were spotted directly onto the agar and the plates were grown overnight at 37 ˚C. The positive control, apramycin (Apra), showed a zone of inhibition whereas none of samples 1-6 showed any activity against the strains tested.
